# Investigating Common Pathogenic Mechanisms between *Homo sapiens* and Different Strains of *Candida albicans* for Drug Design: Systems Biology Approach via Two-Sided NGS Data Identification

**DOI:** 10.3390/toxins11020119

**Published:** 2019-02-15

**Authors:** Shan-Ju Yeh, Chun-Chieh Yeh, Chung-Yu Lan, Bor-Sen Chen

**Affiliations:** 1 Laboratory of Control and Systems Biology, Department of Electrical Engineering, National Tsing Hua University, Hsinchu 30013, Taiwan; m793281@gmail.com (S.-J.Y.); al30428@gmail.com (C.-C.Y.); 2 Institute of Molecular and Cellular Biology, National Tsing Hua University, Hsinchu 30013, Taiwan; cylan@life.nthu.edu.tw; 3 Department of Life Science, National Tsing Hua University, Hsinchu 30013, Taiwan; 4 Department of Electrical Engineering, Yuan Ze University, Chungli 32003, Taiwan

**Keywords:** *C. albicans*, host–pathogen genetic and epigenetic network, pathogenic mechanism, network marker, multiple-molecule drug

## Abstract

*Candida albicans* (*C. albicans*) is the most prevalent fungal species. Although it is a healthy microbiota, genetic and epigenetic alterations in host and pathogen, and microenvironment changes would lead to thrush, vaginal yeast infection, and even hematogenously disseminated infection. Despite the fact that cytotoxicity is well-characterized, few studies discuss the genome-wide genetic and epigenetic molecular mechanisms between host and *C. albicans*. The aim of this study is to identify drug targets and design a multiple-molecule drug to prevent the infection from *C. albicans*. To investigate the common and specific pathogenic mechanisms in human oral epithelial OKF6/TERT-2 cells during the *C. albicans* infection in different strains, systems modeling and big databases mining were used to construct candidate host–pathogen genetic and epigenetic interspecies network (GEIN). System identification and system order detection are applied on two-sided next generation sequencing (NGS) data to build real host–pathogen cross-talk GEINs. Core host–pathogen cross-talk networks (HPCNs) are extracted by principal network projection (PNP) method. By comparing with core HPCNs in different strains of *C. albicans*, common pathogenic mechanisms were investigated and several drug targets were suggested as follows: orf19.5034 (YBP1) with the ability of anti-ROS; orf19.939 (NAM7), orf19.2087 (SAS2), orf19.1093 (FLO8) and orf19.1854 (HHF22) with high correlation to the hyphae growth and pathogen protein interaction; orf19.5585 (SAP5), orf19.5542 (SAP6) and orf19.4519 (SUV3) with the cause of biofilm formation. Eventually, five corresponding compounds—Tunicamycin, Terbinafine, Cerulenin, Tetracycline and Tetrandrine—with three known drugs could be considered as a potential multiple-molecule drug for therapeutic treatment of *C. albicans*.

## 1. Introduction

Candida albicans (*C. albicans*) is considered as a prevalent fungus. Nonetheless, it will become an opportunistic pathogenic fungus based on the host’s condition [1]. This pathogen is a Gram-positive fungus but adapts to anaerobic and aerobic condition. *C. albicans* exists in the oral, vaginal mucosa and gastrointestinal tract of many organisms [1], especially in oral epithelial cell. The relevant diseases include pseudomembranous candidiasis and denture-associated erythematous candidiasis. The former pseudomembranous candidiasis is also known as thrush mainly infecting infants and immunocompromised patients. The latter denture-associated erythematous candidiasis takes place among elderly due to poor cleaning of oral prosthetic devices in the hospital. Additionally, acceptance of cytotoxic chemotherapy will lead to severe immunosuppression and promote mucosal damage which makes *C. albicans* overgrow in the oral epithelial cell to cause oral candidiasis. Meanwhile, a delicate balance clearly exists between the potentially damaging effects of Candida virulence factors and the nature of the immune response elicited by the host [2]. Furthermore, under the limitation of resource in the infected host, there is a competition between *C. albicans* and hosts striving for resource to support their essential functions.

SC5314 and WO-1 are two common strains of *C. albicans* employed in laboratory for clinical research. Compared to SC5314, WO-1 in the white cell transforms to opaque cell with high frequency [3]. In addition, the sequence of *C. albicans* SC5314 is transcribed by previous studies so that *C. albicans* SC5314 is frequently used as a wild-type control derived from common laboratory [4]. Although previous studies did not indicate why *C. albicans* would be separated into different strains, strains SC5314 and WO1 are estimated to be separated from each other by a divergence time of one million years [5]. Both strains of *C. albicans* might exist in human body experiencing constant evolution to adapt for host microenvironment. The OKF6/TERT-2 cell line applied to be a model of the human oral epithelial cell is acquired from human oral keratinocytes. Previous studies usually used TR146 cell to do experiment and employed it for pathogen infection [6]. However, we could not regard TR146 as human oral keratinocytes or true model. Along with the advancement of biological technology, OKF6/TERT-2 cell line is a 3D system which resembles the commercially available system based on the cell line TR146 [7]. The cell line is made up of a multiple layer epithelial structure which is similar to the cells in native oral mucosa. Therefore, it is a better representative of the normal submucosa and true human mucosa.

In the immune system, epithelial cells become the first defense line to antagonize bacterial infection. Nonetheless, under *C. albicans* infection condition, this monolayer of cell surface can be destructed by the pathogen’s hyphae or cell surface proteins, allowing *C. albicans* to enter oral mucosa and motivate oral mucosal immune cells such as macrophages, neutrophils and dendritic cells. Moreover, cell surface proteins of *C. albicans* can degrade host cell surface protein to enter the cell so that the whole *C. albicans* will invade the host cell. *C. albicans* infection often arises after the disturbance of normal oral microbiome following immunocompromised patients including the HIV-infected patients or the broad-spectrum antibiotic treatment. After the decrease of immune system or the interference of the oral microbiota, *C. albicans* can form colonization on oral epithelial cells by hyphal growth, grow hyphae to penetrate cell and yield pathogenic factors to degrade the barrier.

The major pathogenic factor of *C. albicans* is distinguished from two parts. One part is cell wall proteins orf19.1816 (ALS3) and orf19.1321 (HWP1). Previous studies indicate that orf19.1816 will induce endocytosis by binding host cell receptors such as ERBB2, HSP90B1, CDH1 and CDH2 so that it will be considered as an infection initiation [8,9,10,11]. Moreover, orf19.1321 (HWP1) is related to cell adhesion and biofilm formation [12]. Another part includes pathogenic factors released such as orf19.5714 (SAP1), orf19.3708 (SAP2), orf19.6001 (SAP3), orf19.5716 (SAP4), orf19.5585 (SAP5) and orf19.5542 (SAP6). These pathogenic factors indicate to induce inflammatory response and degradation of host cell surface proteins. These pathogenic factors will recruit neutrophils and macrophages for eliminating pathogen and induce a critical inflammatory response. Moreover, not only pathogenic factors but also hyphae growth of *C. albicans* trigger inflammatory response. The morphological transition of *C. albicans* can change yeast to a filamentous form, namely hyphae. In the past, the hyphal of *C. albicans* is discovered and regarded as a virulence factor by previous studies [13]. Further, *C. albicans* will find nutrient sources or metal ion by hyphae growth so that hyphal will penetrate host cell actively to induce an inflammatory response in the host cell. Additionally, *C. albicans* is polymorphic pathogen such as yeast type, pseudohyphae, true hyphae and biofilm [14]. The yeast cell is also differentiated between white cell and opaque cell. Because of polymorphic type of *C. albicans*, some transcription factors (TFs) of *C. albicans* must modulate these patterns. Currently, two TFs of *C. albicans* are orf19.610 (EFG1) and orf19.4433 (CPH1), respectively. Orf19.610 is the most important TF by the indication of previous studies and can regulate morphogenesis such as hyphae, biofilm and white cell [15]. Moreover, Orf19.610 even modulates white cell to opaque cell. Therefore, *C. albicans* WO-1 may be modulated by this TF. On the other hand, orf19.4433 contributes to the pheromone-stimulated biofilm and galactose metabolism. Due to pheromone-stimulated biofilm, *C. albicans* will produce next generation [14].

Even though *C. albicans* is investigated by numerous studies, the cross-talk of inter-species mechanism is less discovered apart from the mentioned above. Mostly, inter-species mechanism is between *C. albicans* and other animals such as zebrafish [16]. Nonetheless, the importance of host–pathogen interactions (HPIs) was recently highlighted in a previous study [16]. Dynamic network modeling, protein interaction databases, and dual transcriptome data from zebrafish and *C. albicans* during infection were used to infer infection-activated host–pathogen dynamic interaction networks [16]. Although the study could supply PPI (protein–protein interaction) model of zebrafish and *C. albicans* to understand innate and adaptive networks, zebrafish is still different from human because of physiological phenomena. Additionally, the cultivated human cell technique was still less mature in the past such as TR146. Not only cell culture technology but also pathogen control is a difficult problem. Therefore, it is hard to simulate the true human model. Due to *C. albicans* WO-1 is susceptible to microenvironment, we rarely use it in the laboratory. Along with the gradual progression of cultivated cell techniques, it is still not easy to look into inter-species infection. Here, the host-pathogen genome-wide genetic and epigenetic interspecies networks (GEINs) established by systems biology approach via big database mining and two-sided next generation sequencing (NGS) data identification could offer us a systematic viewpoint of host cellular functions with pathogen infection. It is noted that we know few about PPIN (protein–protein interaction network) and GRN (gene regulation network) in pathogen–pathogen intra-species and host–pathogen inter-species. With the concept of sequence homology to *Saccharomyces cerevisiae* (*S. cerevisiae*), we infer putative pathogen–pathogen candidate intra-species PPIN and GRN, and putative host–pathogen candidate inter-species PPIN and GRN for *C. albicans* SC5314 and WO-1, respectively. After combining candidate host-host, host–pathogen and pathogen–pathogen PPIN and GRN together, we could have candidate host–pathogen genetic and epigenetic interspecies network (GEIN). Applying system identification and system order detection methods to candidate host–pathogen GEINs, we could obtain real host–pathogen GEINs between human and *C. albicans* SC5314 as well as human and *C. albicans* WO-1. By further analyzing core cross-talk pathways extracted from real host–pathogen GEINs, we could understand common pathogenic mechanisms comprehensively for drug design of *C. albicans* infection.

It is well known that microRNAs (miRNAs) and long non-coding RNAs (lncRNAs) would modulate gene expression. miRNAs are short non-coding RNA molecules (21–23 nucleotides). By binding with mRNA-induced silencing complex, host cell genes will be silenced to decrease cellular progression. Surprisingly, recent studies suggest that host cell employs microRNA silencing to repress *C. albicans* gene [17], indicating that miRNAs play an important role in pathogen and host gene modulation. Moreover, lncRNAs are essential for the epigenetic regulation of host cell in recent years. In controlling various cellular responses, lncRNAs participate gene regulation in a similar manner but with a more complicated process than miRNAs. However, recent researches do not suggest that lncRNAs modulate fungus gene for controlling pathogen responses. Furthermore, we also consider other epigenetic modifications such as histone modification and DNA methylation which give cell responses to pathogen invasion and host defense. These activities mentioned above will alter the cross-talk behavior between pathogen infection and host cell adaption.

In order to investigate the common and specific host–pathogen cross-talk mechanisms and epigenetic activities contributing to infection progression during different strains of *C. albicans*, we then recognized real host–pathogen cross-talk GEINs between human oral epithelial cells (OKF6/TERT-2 cell) and *C. albicans* SC5314 as well as *C. albicans* WO-1. Next, we extracted the core host–pathogen cross-talk networks (HPCNs) from real host–pathogen cross-talk GEINs by principal network projection (PNP) method. We then projected core HPCNs in respect of KEGG pathways to discover the core pathways involved in common and specific cellular responses of host and pathogen between different strains of *C. albicans* during the infection process. Additionally, we discussed the offensive and defensive mechanisms between host and different strains *C. albicans* to identify important biomarkers for the pathogenesis of *C. albicans* infection. In this study, we indicated that orf19.5034 (YBP1) has the anti-ROS ability, and orf19.939 (NAM7), orf19.2087 (SAS2), orf19.1093 (FLO8) and orf19.1854 (HHF22) play an important common role in hyphae growth and pathogenesis in the infection of *C. albicans* SC5314 and *C. albicans* WO-1. Moreover, orf19.5585 (SAP5), orf19.5542 (SAP6) and orf19.4519 (SUV3) will cause biofilm formation. Additionally, orf19.7247 (RIM101) coordinates other pathogen proteins for the degradation of host cell protein CDH1. Further, previous studies indicated that orf19.1816 (ALS3), orf19.610 (EFG1), orf19.1321 (HWP1), orf19.4433 (CPH1) and orf19.723 (BCR1) are considered as important roles to endocytosis, morphological transformation, which could also be verified by these studies [12,14,15,18].

Eventually, based on our results, we proposed pathogen proteins mentioned above as potential drug targets for drug discovery in the infection of *C. albicans* because of their crucial roles in the common pathogenic mechanisms between different strains of *C. albicans*. Therefore, multi-molecules drugs such as Terbinafine, Cerulenin, Tunicamycin, Tetrandrine and Tetracycline are introduced to target multiple potential drug targets for the therapeutic treatment of different strains of *C. albicans*.

## 2. Results

### 2.1. The Identified GEINs under the Infection of C. albicans SC5314 and C. albicans WO-1

The real GEINs of *C. albicans* SC5314 infection in OKF6/TERT-2 cells of two replicates are shown in Figure S3A,B in Supplementary Materials, respectively, by applying network visualizing software Cytoscape [19]. Similarly, the real GEINs of *C. albicans* WO-1 infection in OKF6/TERT-2 cells of two replicates are shown in Figure S3C,D in Supplementary Materials, respectively. The number of identified nodes and edges are shown in Table 1 and Table 2, respectively. For two replicates of *C. albicans* SC5314, the number of nodes of replicate 1 is higher than replicate 2; and the identified edges in Table 2 show significant differences between two replicates. By contrast, for two replicates of *C. albicans* WO-1, the number of nodes of replicate 1 is similar to replicate 2; and the identified edges in Table 2 show little differences between two replicates. Compared to *C. albicans* WO-1, *C. albicans* SC5314 easily produces the individual difference in infection progression. Overall, *C. albicans* WO-1 is more stable than *C. albicans* SC5314 in infection progression.

To further find gene functions in OKF6/TERT-2 cells during the infection of two strains of *C. albicans* according to their functional groups, we exhibited the specific host cellular functions and analyzed the functional abundance of related pathways of the conserved host target-genes among 2 replicates on the basis of gene ontology (GO) terms by applying the DAVID analysis [20]. In Table 3, the infection progression of *C. albicans* SC5314 was characterized by the redistribution of epithelial cell barrier, cell shape and cell adhesion so that they can activate oxidation response to inflammation response and metal-binding, which could act as an important character in the struggle for nutrients and metal material between host and pathogen. While considering the infection progression of *C. albicans* WO-1, it is similar to *C. albicans* SC5314. However, in addition to finding host gene functions, pathogen gene functions were also found. Based on Candida Genome Database, specific pathogen functions with a plenty of conserved pathogen target-genes among 2 replicates are found by applying Gene Ontology Term Finder. In Table 4, gene functions of *C. albicans* SC5314 were characterized by epithelial cell barrier so that *C. albicans* SC5314 can make morphological transition due to structural molecule activity and molecular function regulation. Besides, *C. albicans* SC5314 can invade oral epithelial cell via the induced endocytosis and active penetration which are applied by hydrolase activity, protein binding and structural molecule activity. While considering gene functions of *C. albicans* WO-1, it is the same as *C. albicans* SC5314.

Since the real GEINs are very complicated, it is still hard to investigate common and specific infection pathogenic mechanism from these networks directly. We thereby used PNP approach (see Section 1.5 in Supplementary Materials) to find the corresponding core HPCNs in Figures S4 and S5 (see Supplementary Materials) from real GEINs of OKF6/TERT-2 cells during the infection of *C. albicans* WO-1 and SC5314, respectively. By this approach, we can further investigate common and specific pathogenic mechanisms of the *C. albicans* infection in respect of KEGG pathways.

### 2.2. The Core Host-pathogen Cross-talk Networks (HPCNs) during the Infection of C. albicans SC5314 and C. albicans WO-1

Employing PNP approach to real GEINs in Figure S3A,B in Supplementary Materials, we could evaluate the projection value of each node by the Equation (40) in Supplementary Materials for the building of core HPCNs. Host proteins with top 5000 projection values and pathogen protein with top 1500 projection values based on intra-species ranking in all two replicates and their connected genes/miRNAs/lncRNAs were selected as core HPCN of two replicates. Since the recognized real GEINs in Figure S3A,B in Supplementary Materials, respectively, are part of two replicates, which are biological replicates from the same cell line. The recognized differential regulations and interactions can be considered as the adaptability of cells while confronting stimulus and stress at different replicates. For more intact information, the combinations of these interactions and regulations in two replicates are viewed as real GEIN as shown in Figure S3E. Next, we extracted core nodes from the real GEIN in Figure S3E by PNP approach (see Section 1.5 in the Supplementary Materials) to construct core HPCN as shown in Figure S4 during the infection of *C. albicans* SC5314. Likewise, core HPCN of *C. albicans* WO-1 which is shown in Figure S5 was constructed by the same procedure.

Comparing Figures S4 and S5 in Supplementary Materials, the number of core proteins in the core HPCN during the infection of *C. albicans* WO-1 is higher than that in the core HPCN during the infection of *C. albicans* SC5314. The *C. albicans* SC5314 infection exists a number of individual differences in host cells. These differences lead to various projection values causing relatively diverse signaling pathways in two replicates. In contrast, the *C. albicans* WO-1 infection holds consistent high projection values causing similar signaling pathways in two replicates. Moreover, only the pathogen proteins with top 1500 projection values and host proteins with top 5000 projection values were selected as core network nodes of core HPCN in the infection of *C. albicans* SC5314. The same criteria apply to choose core network nodes of core HPCN in the infection of *C. albicans* WO-1.

**Figure 1:**
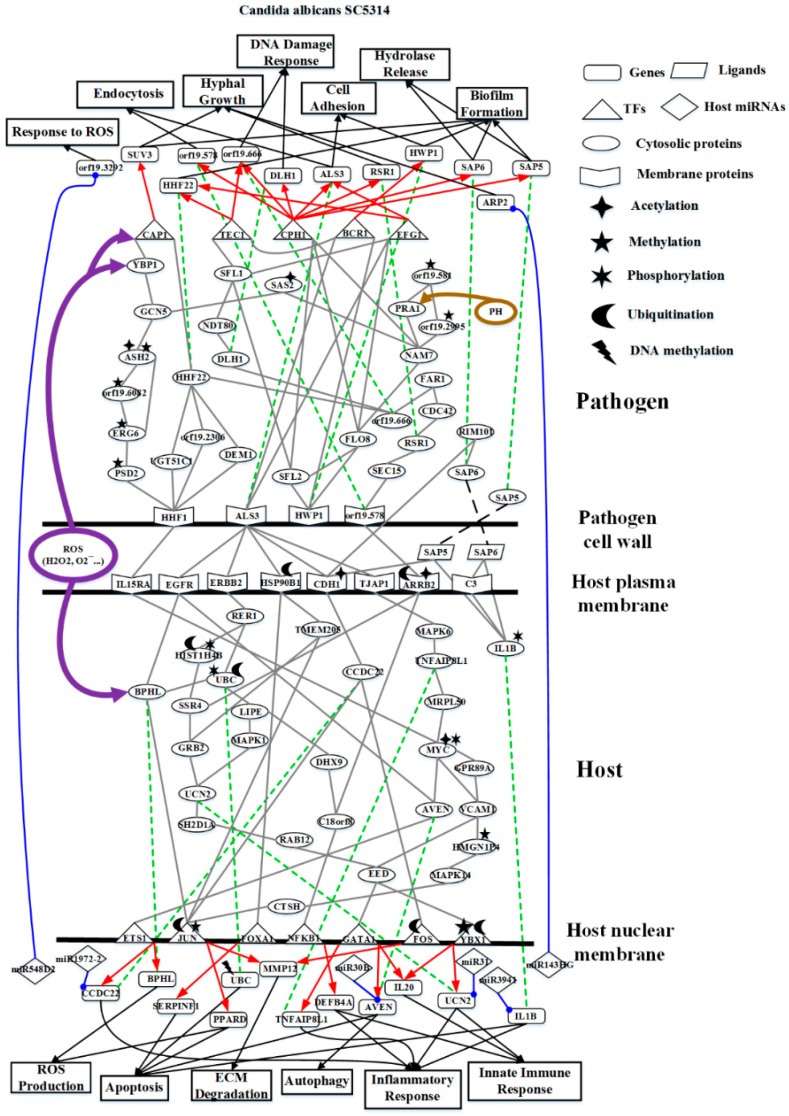
The core cross-talk pathways extracted and rearranged based on KEGG pathways from the core HPCN in Figure S4 during the *C. albicans* SC5314 infection. The upper layer is the pathogen core pathways and the lower layer signifies the host core pathways during the *C. albicans* SC5314 infection. The grey lines represent the protein–protein interaction; the red arrow lines are transcriptional regulation; the green dot lines denote the protein translation; the black dash lines indicate the protein secretion; the blue lines with circle endpoint represent miRNA repression and the circles with purple frame and arrow lines represent the production activity and response of reactive oxygen species (ROS). The pathogenic factor orf19.1816 (ALS3) of *C. albicans* SC5314 triggers to induce endocytosis. The TFs orf19.1623 (CAP1) and orf19.5034 (YBP1) are pathogenic factor of *C. albicans* SC5314 to react via ROS of host production. Moreover, OKF6/TERT-2 cells apply autophagy and immune response to recruiting immune cells such as macrophages and neutrophils to eliminate *C. albicans* SC5314. Finally, the endoplasmic reticulum stress reflects on the accumulated cellular stress and host cell extrusion so that host cells will produce severe inflammatory response and cause apoptosis process. In addition, pathogenic factors orf19.5585 (SAP5) and orf19.5542 (SAP6) also generate inflammation response and apoptosis process of the host cell.

**Figure 2:**
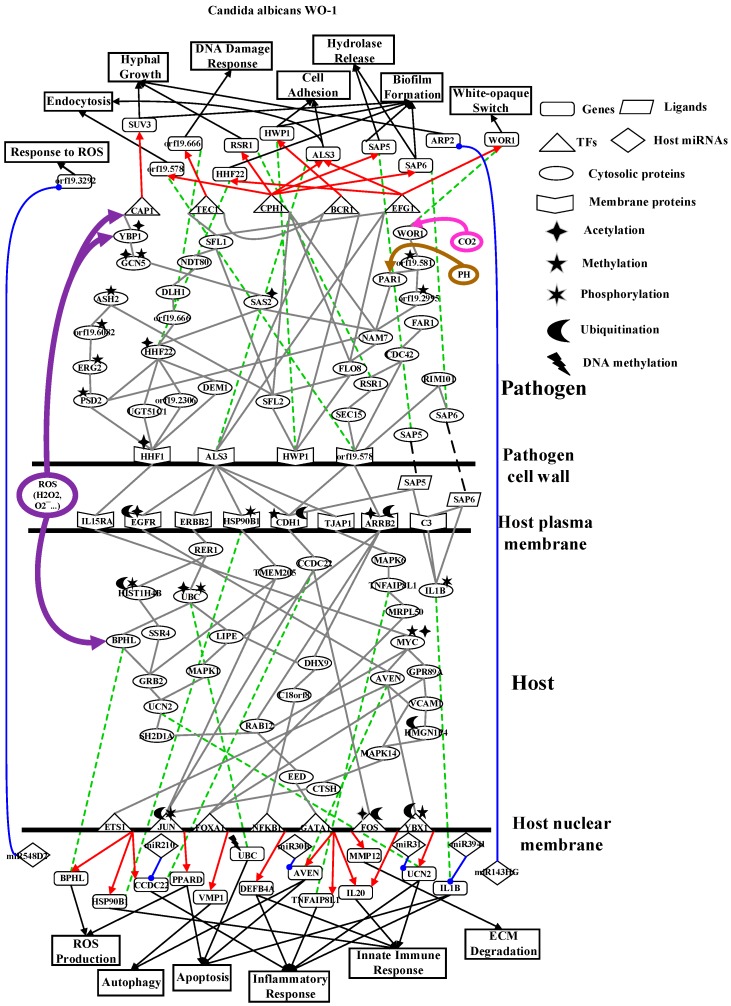
The core cross-talk pathways extracted and rearranged based on KEGG pathways from the core HPCN in Figure S5 during the *C. albicans* WO-1 infection. The upper layer is the pathogen core pathways and the lower layer signifies the host core pathways during the *C. albicans* WO-1 infection. The grey lines represent the protein–protein interaction; the red arrow lines are transcriptional regulation; the green dot lines denote the protein translation; the black dash lines indicate the protein secretion; the blue lines with circle endpoint represent miRNA repression and the circles with purple frame and arrow lines represent the production activity and response of ROS. The pathogenic factor CAWG_02005 (ALS3) of *C. albicans* WO-1 triggers to induce endocytosis. The TFs CAWG_02548 (CAP1) and CAWG_00057 (YBP1) pathogenic factor of *C. albicans* WO-1 will react via ROS of host production. Moreover, OKF6/TERT-2 cells apply autophagy and immune response to recruiting immune cells such as macrophages and neutrophils to eliminate *C. albicans* WO-1. Finally, the endoplasmic reticulum stress reflects on the accumulated cellular stress and host cell extrusion so that host cells will produce severe inflammatory response and cause apoptosis process. In addition, pathogenic factors CAWG_05098 (SAP6) and CAWG_05066 (SAP5) also lead to inflammation response and apoptosis process of the host cell.

To further investigate common and specific progression genetic and epigenetic mechanisms between *C. albicans* SC5314 and *C. albicans* WO-1 from Figures S4 and S5 in Supplementary Materials, based on the projection value of each element in the core HPCNs, we then construct core host-pathogen signaling pathways for two strains of *C. albicans*. For core host signaling pathways, we select core membrane proteins including core receptors, core TFs, core proteins, and their regulatory miRNAs and lncRNAs. On the other hand, we construct core pathogen signaling pathways by selecting cell wall core proteins, signal transmission core pathogen proteins and core pathogen TFs. In order to make signal transduction pathways complete, we not only have to consider elements mentioned above but also the role of epigenetic modifications such as acetylation, methylation, ubiquitination and phosphorylation. These epigenetic modifications decided by basal levels $\chi_{i}^{H}$ and $\chi_{j}^{P}$ in the host and pathogen PPIN in Equations (1) and (2) shown in Supplementary Materials, respectively. Here, basal levels denote unknown interactions which have not been mentioned in stochastic dynamic interactive equations. When a PPI basal level exceeds a threshold, the core proteins with an overtaking threshold of basal level in infection progression were speculated that these core proteins may be affected by epigenetic modification such as acetylation, methylation, ubiquitination and phosphorylation. Moreover, the genes with an overtaking threshold of basal level in infection progression were speculated that these core genes may be influenced by DNA methylation. The core cross-talk pathways of each strain in infection progression are described in the following and shown in Figure 1 and Figure 2.

### 2.3. Analysis of Core Interspecies Pathways to Investigate Host/Pathogen Cross-Talk Common and Specific Pathogenic Progression Mechanisms during C. albicans SC5314 Infection

As shown in Figure 1, in the infection progression of *C. albicans* SC5314, orf19.1816 (ALS3) plays a significant role in cell adhesion and endocytosis induction by interacting with EGFR, ERBB2 (HER2), CDH1 (E-cadherin), HSP90B1 and TJAP1 (TJP4) [8,9,10,11]. However, orf19.1321 (HWP1) also has influences on the cell adhesion and maintenance of hyphal cell wall [12]. Orf19.610 (EFG1) and orf19.4433 (CPH1) are important TFs for regulating biofilm, hyphal growth and virulence [12,14,15]. Through pathogen cell surface proteins, orf19.1816 (ALS3) interacts with orf19.610 (EFG1) and orf19.723 (BCR1) to modulate TF orf19.610 (EFG1) and orf19.723 (BCR1), respectively [18,21]. The TF orf19.610 (EFG1) which is also mediated by orf19.454 (SFL1) positively regulates cell adhesion and endocytosis-related gene *orf19.1816* (ALS3) and biofilm-related gene *orf19.1854* (HHF12). The TF orf19.723 (BCR1), which is also triggered by the influence of TF orf19.5908 (TEC1), negatively regulates cell adhesion and biofilm-related gene *orf19.1321* (HWP1) (*p*-value < 1 × 10^−16^). On the other hand, one of pathogen cell surface proteins, orf19.1321 (HWP1) triggers TFs orf19.5908 (TEC1), orf19.610 (EFG1), orf19.723 (BCR1) and orf19.4433 (CPH1) by signaling proteins orf19.3969 (SFL2), orf19.454 (SFL1) and orf19.1093 (FLO8) [22,23]. The TF orf19.4433 (CPH1) positively regulates gene *orf19.3760* (DLH1) (*p*-value < 1.5 × 10^−6^), endocytosis-related gene *orf19.578*, and both genes *orf19.5542* (SAP6) and *orf19.5585* (SAP5) which are related to hydrolytic activity and biofilm formation. Furthermore, the TF orf19.4433 (CPH1) negatively regulates DNA damage-related gene *orf19.666* which is also positively regulated by TF orf19.5908 (TEC1)*,* cell adhesion and endocytosis-related gene *orf19.1816* (ALS3) and hyphae growth-related gene *orf19.2614* (RSR1).

The other two pathogen cell surface proteins are orf19.578 and orf19.1059 (HHF1). Orf19.578 binds to ARRB2 so that it can induce endocytosis and exocytosis. However, after receiving the upstream signal, orf19.578 can activate TFs orf19.1623 (CAP1) and orf19.5908 (TEC1) through signaling proteins orf19.1418 (SEC15), orf19.2614 (RSR1), orf19.390 (CDC42), orf19.7105 (FAR1), orf19.666, orf19.3760 (DLH1), orf19.2119 (NDT80), orf19.454 (SFL1), and orf19.1854 (HHF22). The TF orf19.1623 (CAP1) positively regulates hyphae growth and biofilm-related gene *orf19.4519* (SUV3). The TF orf19.5908 (TEC1) positively regulates gene *orf19.666* and negatively modulates *orf19.1854* (HHF22), respectively. Finally, some of *C. albicans* SC5314 can go into the host cell by the induced endocytosis, and the others of *C. albicans* SC5314 form colony morphology by orf19.1059 binding to IL15RA. After receiving signal, orf19.1059 (HHF1) can trigger TFs orf19.1623 (CAP1) and orf19.5908 (TEC1) via signaling proteins orf19.3954 (PSD2), which is affected by orf19.1631 (ERG6)-induced methylation, orf19.1631 (ERG6), which is affected by orf19.3964 (ASH2)-induced methylation, orf19.6082, which is influenced by orf19.169 (CHO2)-induced methylation, orf19.3964 (ASH2), which is influenced by orf19.1631-induced methylation and orf19.705-induced acetylation, orf19.705 (GCN5), orf19.5034 (YBP1), orf19.2616 (UGT51C1), orf19.2306, orf19.4392 (DEM1), orf19.1854 (HHF22), orf19.666, orf19.3760 (DLH1), orf19.2119 (NDT80) and orf19.454 (SFL1) [24,25]. In addition, orf19.2087 (SAS2), which is affected by orf19.705-induced acetylation, triggers TF orf19.1623 (CAP1) indirectly and binds to orf19.939 (NAM7) interacting with orf19.1093 (FLO8). Therefore, orf19.939 (NAM7), which interacts with the above mentioned proteins orf19.1093 (FLO8) and orf19.2087 (SAS2), can activate downstream proteins such as orf19.2995, which is affected by the orf19.3295-induced methylation, orf19.3111 (PRA1), which results in hyphae growth by neutral and alkaline pH, and orf19.581, which activates the above mentioned signals orf19.2995 and orf19.3111 (PRA1) and simultaneously is induced with methylation itself by orf19.2575. When hyphae initiate growth, *C. albicans* SC5314 can release protease such as orf19.5585 (SAP5) and orf19.5542 (SAP6) by hyphae. Moreover, the protease orf19.5585 (SAP5) cooperates with pathogen protein orf19.7247 (RIM101) to degrade CDH1. Accordingly, some proteins of *C. albicans* 5314 proteins can go into the host cell easily and initiate the degradation of membrane proteins which are involved in extracellular matrix (ECM).

In response to pathogen infection, the receptors such as EGFR, ERBB2, CDH1, HSP90B1, and TJAP1 at the host membrane interact with pathogen cell wall proteins to start inducing endocytosis. First, receptor EGFR activates downstream proteins BPHL and AVEN to modulate TFs, JUN (also known as c-Jun), which is affected by the USP12PX-induced ubiquitination and PRMT1-induced methylation, and ETS1, respectively (see Figure 1) [26]. The TF ETS1 (also known as ETS-1) positively regulates gene *BPHL* involved in ROS production and inflammatory-related gene *CCDC22* (CCDC22: *p*-value < 7 × 10^−3^). Another TF JUN positively regulates ECM degradation through gene *MMP12* and negatively regulates ROS production through apoptosis-induced gene *PPARD*, respectively. Secondly, receptor ERBB2 triggers TFs NFKB1 (subunit of NF-κB), YBX1 (also known as YBX-1)and GATA1 by signaling proteins, RER1, HIST1H4B which is influenced by NEURL4-induced ubiquitination and PLA2G12B-induced phosphorylation, UBC affected by OTUB1-induced ubiquitination and INPP4B-induced phosphorylation and interacted with BPHL, SSR4, LIPE, MAPK1 (also known as p38, ERK, PRKM1), GRB2, UCN2, SH2D1A, RAB12, DHX9(also known as DDX9), C18orf8 and EED which modulates both YBX1 and GATA1 [27]. TF NFKB1 could negatively regulate the resistance to gene *DEFB4A* having defense in response to Gram-positive bacterium [28,29]. Both TFs YBX1 and GATA1 positively regulate gene *IL20* (YBX1: *p*-value < 1 × 10^−6^, GATA1: *p*-value < 1 × 10^−3^) which is involved in initiating innate immune response. However, TF YBX1 with SHMT1-induced methylation and USP24-induced ubiquitination also negatively regulates the innate immune and inflammation-related gene *UCN2* (*p*-value < 8 × 10^−6^). In addition, TF GATA1 simultaneously positively regulates gene *AVEN* which can eliminate fungus by autophagy and induce apoptosis and inflammation-related gene *TNFAIP8L1* [30]. Thirdly, receptor HSP90B1, which is affected by USP11-induced ubiquitination, binds to TF FOXA1 and TMEM205 which modulates TF JUN and plays an important role in chemotherapeutic agent [31]. The TF FOXA1 then negatively regulates to induce apoptosis-related gene *SERPINF1* [32]. Fourthly, receptor CDH1, which is affected by the NAT10-induced acetylation and binds to protease orf19.5585 (SAP5), triggers TFs JUN and FOS (also known as c-Fos) through signaling protein CCDC22 [33]. The TF FOS affected by the UBFD1-induced ubiquitination also negatively regulates gene *MMP12* as mentioned above [34]. Finally, the receptor TJAP1 modulates TFs ETS1, YBX1, GATA1 and JUN by signaling proteins, MAPK6 (also known as ERK3), TNFAIP8L1, MRPL50, MYC (also known as C-Myc), which is affected by the NAALADL1-induced acetylation and PDPR-induced phosphorylation and activates downstream proteins VCAM1 and AVEN, GPR89A, UCN2, VCAM1, HMGN1P4 which is affected by the KDM4A-induced methylation, MAPK14 (also known as p38, PRKM14), CTSH and AVEN (see Figure 1). Besides, the receptor ARRB2, which is affected by HDAC1-induced acetylation and HERC5-induced ubiquitination, binds to orf19.578 so that it can induce endocytosis and activate downstream proteins SSR4 and C18orf18 to modulate TF NFKB1 [35]. On the other hand, receptor IL15RA receives orf19.1059 (HHF1) of cell colony signal to interact with MYC. Eventually, from the released signals by *C. albicans* SC5314, the receptor C3 (also known as C3a and C3b) binds to orf19.5542 (SAP6) to activate downstream protein IL1B (also known as IL-1β) which also is affected by the PPP2CA-induced phosphorylation and interacted with orf19.5585 (SAP5) [12,36,37]. When IL1B was stimulated by these signals mentioned above, it can evoke apoptosis and inflammatory response [38].

Apart from the epigenetic regulations in core pathways mentioned above, we also discovered epigenetic regulations of miRNAs, including miR-30B, miR31, miR3941, miR143HG, miR1972-2 and miR548D2. MiR-30B inhibits *AVEN* during *C. albicans* SC5314 infection to reduce autophagy and apoptosis [39]. *UCN2* and *CCDC22* are silenced by miR31 and miR1972-2 to decrease inflammatory response, respectively. In addition to inflammatory response, *UCN2* also decreases innate immune response by miR31. However, IL1B was stimulated to initiate apoptosis and inflammatory response, and miR3941 also inhibits *IL1B* to reduce tissue necrosis. Moreover, we found miR548D2 inhibits *orf19.3292* to strength the effect of ROS which was first produced by host cell. Besides, orf19.7292 is silenced by miR143HG to reduce hyphae growth of pathogen. Finally, there is one gene *UBC* with an overtaking threshold basal level, indicating that this might have been resulting in DNA methylation via infection progression [40].

### 2.4. Analysis of Core Interspecies Pathways to Investigate Host/Pathogen Cross-talk Common and Specific Pathogenic Mechanisms during C. albicans WO-1 Infection

As shown in the core cross-talk pathways of Figure 2, our results indicate most interactions and regulations are the same as Figure 1. Accordingly, we only discuss different interactions and regulations in *C. albicans* WO-1 infection from *C. albicans* SC5314 infection. For pathogen interactions, the CAWG_01529-induced (orf19.2575) methylation of CAWG_00472 (orf19.581) simultaneously influences upstream proteins CAWG_3130 (PRA1) and CAWG_01080 (orf19.2995) and downstream protein CAWG_00418 (WOR1). Nonetheless, CAWG_00418 (WOR1) is subjected to not only protein CAWG_04472 (orf19.581) but also microenvironment filled with CO_2_ so that CAWG_00418 (WOR1) can transform white cell to opaque cell. Furthermore, CAWG_02083 (EFG1) is also the main regulation of white-to-opaque switch of gene *CAWG_00418* (WOR1) so that yeast cell of *C. albicans* WO-1 can transform white cell to opaque cell [41]. Other pathogen interactions signify that CAWG_04469 (orf19.578) can not trigger any TFs by interaction between CAWG_05375 (FAR1) and CAWG_00299 (orf19.666) but interact with CAWG_00581 (CDC42) directly. Moreover, CAWG_04844 (ASH2) does not bind to CAWG_01970 (GCN5) due to reducing epigenetic modification of CAWG_01970 (GCN5). Finally, because of the acetylation of CAWG_00969 (HHF1), CAWG_04836 (PSD2) could directly regulate CAWG_02542-induced methylation and be indirectly mediated by acetylation to increase interactions with CAWG_04444 (NAM7). On the other hand, in the pathogen regulations, it is noted that CAWG_00682 (CPH1) does not regulate DNA damage-related gene *CAWG_00299* (orf19.666); TF CAWG_02766 (TEC1) does not regulate DNA damage-related gene *CAWG_01979* (orf19.666). Instead, the TF CAWG_02766 (TEC1) modulates cellular functions such as hyphae growth, biofilm formation and white cell pheromone response. Due to the regulatory function of TF CAWG_02766 (TEC1), our result may imply that *C. albicans* WO-1 could transform opaque cell in the infection progression to reduce the regulation of CAWG_01979 (HHF22).

On the host side, similarly, we only discuss different interactions and regulations in *C. albicans* WO-1 infection from Figure 2. For host interactions, MRPL50 via *C. albicans* WO-1 infection directly triggers TF FOXA1 but not protein MYC (also knowns C-Myc) in *C. albicans* SC5314 infection. Moreover, EED does not simultaneously trigger two TFs GATA1 and YBX1 from our result. Instead, TF YBX1 is activated by protein AVEN. Due to different epigenetic modifications of MYC, MYC cannot trigger protein VCAM1 but further increase downstream protein interactions. For example, AVEN triggers MAPK14 (also known as p38, PRKM14) via VCAM1. Furthermore, receptor EGFR with NAALADL2-induced acetylation and OTUD3-induced ubiquitination activates less proteins such as only AVEN. Compared to *C. albicans* SC5314 infection, EGFR triggers two proteins BPHL and AVEN. Therefore, the activity of EGFR is limited to epigenetic modification in our result. Moreover, BPHL binds to GRB2 under *C. albicans* WO-1 infection via UBC. We can infer that UBC affected by NAALADL2-induced acetylation and PPP4R4-induced phosphorylation activates different interactions. By contrast, the receptor HSP90B1 cannot transmit stimulation signals to TF GATA1 due to GAPDHP24-induced phosphorylation. Therefore, we can infer that phosphorylation will produce inhibition. Finally, the HGSNAT-induced acetylation and USP41-induced ubiquitination of receptor ARRB2 activate downstream proteins such as LIPE, DHX9 and RAB12. Conversely, in *C. albicans* SC5314 infection, ARRB2 with the same epigenetic modification triggers different proteins such as SSR4 and C18orf8. It is noted that different epigenetic proteins could cause regulations of TF to strengthen the corresponding cellular response. Subsequently, when HSP90B1 with GAPDHP24-induced phosphorylation further triggers the regulation of TF ETS1, HSP90B1 with ubiquitination will not activate TF ETS1 in the infection of *C. albicans* SC5314. We may speculate that ubiquitination modification may degrade a receptor to reduce its regulation of TF. In addition, CCDC22 receives the upstream signal from receptor CDH1 influenced by MTAP-induced methylation and USP47-induced ubiquitination and results in repression of different miRNAs such as mir210. In conclusion, distinct epigenetic modifications will lead to different host cellular responses from the exhibition of our results. The details will be discussed in the next section.

## 3. Discussion

From Figure 1 and Figure 2, we identified the host–pathogen core cross-talk pathways during the infection of *C. albicans* SC5314 and *C. albicans* WO-1 by systems biology methods (refer to Supplementary Materials), respectively. Overall, there is a little difference between pathogenic mechanisms of two strains of *C. albicans* such as white-to-opaque switch. One study indicated *C. albicans* WO-1 could change white cell to opaque cell at high frequency compared to *C. albicans* SC5314 [42]. We found even exist this phenomenon, there are common pathogenic mechanisms between *C. albicans* SC5314 and WO-1. In addition, *C. albicans* usually maintain in white cell in normal microenvironment. Perhaps, it is the difficult environment forcing *C. albicans* to switch in opaque cell. Previous study suggests that opaque cells keep their own cell type in certain conditions including carbon dioxide, anaerobic growth and acidic (pH < 7) making them proceed sexual reproduction [43].

In other words, we also could imply that *C. albicans* WO-1 may transform opaque cell to white cell immediately due to external microenvironment. Moreover, the *MTLa* locus of *C. albicans* WO-1 is absent in all other strains of *C. albicans*. These would lead to mutation and genetic diversity occurring easily to *C. albicans* SC5314 [40].

According to Table 1 and Table 2, *C. albicans* WO-1 is more stable than *C. albicans* SC5314. Hence, pathogen proteins within top 1500 projection values and host proteins with top 5000 projection values in PNP projection method (see Equation (40) in Supplementary Material) can be analyzed based on KEGG pathways to discover common pathogenic mechanism. In Figure 1 and Figure 2, we extracted the specific core cross-talk pathways to investigate *C. albicans* infection mechanism within different strains, respectively. Discussing from outside to inside of the host cell, we infer that *C. albicans* becomes harmful from harmless on the oral epithelial cell. In the following subsections, we will extract three figures from Figure 1 and Figure 2 to discuss the common and specific pathogenic mechanism of two strains of *C. albicans*. At last, based on their common pathogenic mechanism, we could propose common drugs to treat the infection of different strains of *C. albicans*.

### 3.1. Defensive Mechanism of OKF6/TERT-2 Cell and the Offensive Mechanism of Different Strains of C. albicans at Host Cell Surface

**Figure 3:**
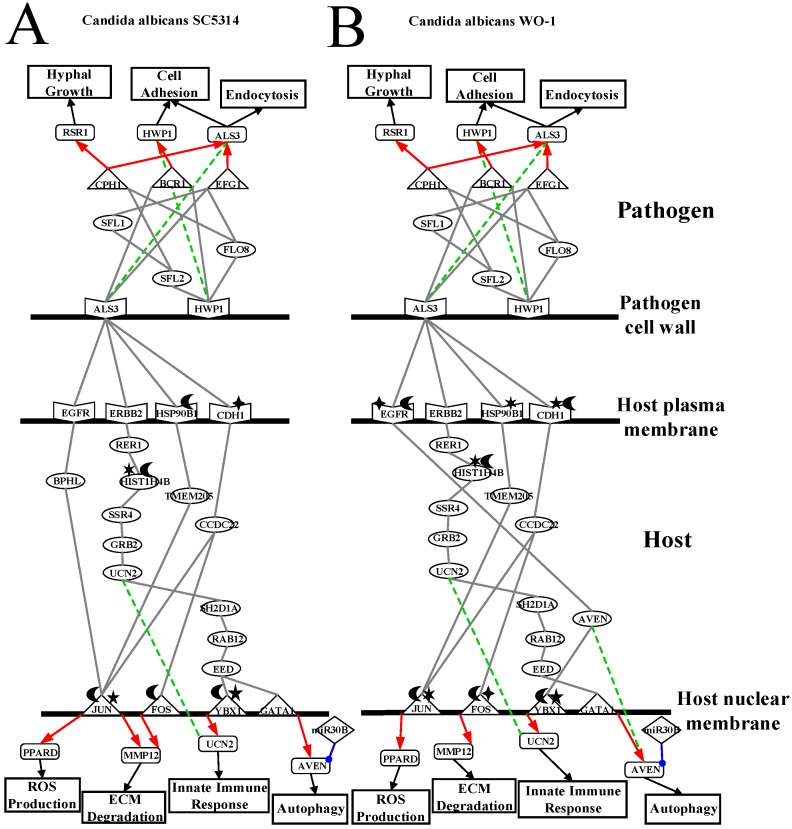
The specific and common host defense mechanism in the infection of different strains of *C. albicans* are extracted from Figure 1 and Figure 2. (**A**) OKF6/TERT-2 cells take a defense strategy against *C. albicans* SC5314 at the beginning of infection. (**B**) OKF6/TERT-2 cells take a defense strategy against with *C. albicans* WO-1 at the beginning of infection. The red arrow lines represent transcriptional regulation; the grey solid lines signify the protein–protein interaction; the green dot lines indicate the protein translation; the blue lines with circle endpoint represent miRNA repression. In (**A**), when *C. albicans* SC5314 infects OKF6/TERT-2 cells, host cells will produce immune response, autophagy and ROS production to defend against pathogen. Moreover, *C. albicans* SC5314 begins to grow hyphae and adhere to host cell surface proteins so that it increases invasion for starving nutrient source. The induction of endocytosis is also beneficial to *C. albicans* SC5314 invasion. Similarly, in (**B**), when *C. albicans* WO-1 infects OKF6/TERT-2 cells, host cells will produce immune response, autophagy and ROS production to defend against pathogen. Moreover, *C. albicans* WO-1 begins to grow hyphae and adhere to host cell surface proteins so that it increases invasion for starving nutrient source. The induction of endocytosis is also beneficial to *C. albicans* WO-1 invasion.

As shown in Figure 3A, *C. albicans* SC5314 is commensal on human oral epithelial cell. However, pathogen cell surface protein orf19.1816 (ALS3) binds to receptors CDH1 (also known as E-cadherin), ERBB2 (also known as HER2), EGFR and HSP90B1 so that these receptors will be degraded and induced in endocytosis. In the infection progresses, *C. albicans* SC5314 will invade a host cell through endocytosis and begin invasion. Receptor CDH1 with the NAT10-induced acetylation activates downstream TFs JUN (also known as c-Jun) and FOS (also known as c-Fos) through signaling protein CCDC22, which is involved in trafficking between the trans-Golgi network and vesicles in the cell periphery. Therefore, TF FOS affected by UBFD1-induced ubiquitination negatively regulates gene *MMP12*, which is positively regulated by TF JUN to result in the degradation of extracellular matrix (ECM). TF JUN affected by USP12PX-induced ubiquitination and PRMT1-induced methylation positively regulates gene *PPARD* so that host cells will produce ROSs to eliminate *C. albicans* SC5314. Moreover, HSP90B1 with the USP11-induced ubiquitination binds to proteins TMEM205, which is related to chemotherapeutic agent, to trigger TF JUN. Previous studies indicate that after accepting chemotherapeutic agent, *C. albicans* will invade host cell and recover itself in immune-compromised patients [44]. Next, receptor EGFR activates TF Jun via BPHL to regulate biological oxidations and hydrolase activity. Finally, the receptor ERBB2 triggers TFs YBX1 and GATA1 through a sequence of signaling protein RER1 to regulate ER protein, HIST1H4B, which is affected by the NEURL4-induced ubiquitination and PLA2G12B-induced phosphorylation-involved histone binding, SSR4, GRB2 influencing cell death, UCN2, SH2D1A related to immune regulatory interactions, RAB12 involved in autophagy modulation, and EED. TF YBX1 with the SHMT1-induced methylation and USP24-induced ubiquitination negatively regulates innate immune-related gene *UCN2*. TF GATA1 positively regulates gene *AVEN* which can eliminate *C. albicans* SC5314 by autophagy. However, because pathogen is not considered as a danger signal, miR-30B represses gene *AVEN* to reduce autophagy function. Then, because gene *MMP12* regulation results in ECM degradation such as collagen, vitronectin, laminin and fibronectin, *C. albicans* SC5314 starves to nutrient or carbon sources.

*C. albicans* SC5314 begins to grow hyphae and invade epithelial cells. Orf19.1816 (ALS3) triggers TFs orf19.610 (EFG1) and orf19.723 (BCR1) by directly binding to these TFs. The TF orf19.610 positively regulates gene *orf19.1816* so that *C. albicans* can invade host cell and strengthen cell adhesion to anchor host cell surface. The TF orf19.723 (BCR1) can negatively regulate gene *orf19.1321* (HWP1) which is related to biofilm forming and cell adhesion. In addition, pathogen receptor orf19.1321 can activate downstream pathogen proteins orf19.3969 (SFL2) to regulate *C. albicans* morphogenesis through signaling protein orf19.454 (SFL1) for triggering TF orf19.610 (EFG1), and orf19.1093 (FLO8) to the hyphal formation through regulating TFs orf19.610 (EFG1), orf19.723 (BCR1) and orf19.4433 (CPH1). The TF orf19.4433 (CPH1) also negatively regulates the hyphae growth-related gene *orf19.2614* (RSR1). According to previous studies, yeast cell of *C. albicans* damages macrophages via hyphae growth to reduce the destruction of *C. albicans* [45].

As shown in Figure 3B, *C. albicans* WO-1 is commensal on human oral epithelial cell. However, pathogen cell surface protein CAWG_2005 (ALS3) binds to receptors CDH1 (also known as E-cadherin), ERBB2 (also known as HER2), HSP90B1 and EGFR so that these receptors will be degraded and induced in endocytosis. As infection progresses, *C. albicans* WO-1 will invade a host cell through endocytosis and begin invasion. Compared to *C. albicans* SC5314, the receptor CDH1 with the MTAP-induced methylation and USP47-induced ubiquitination activates to downstream TFs JUN (also known as c-Jun) and FOS (also known as c-Fos) through CCDC22, which is involved in trafficking between the trans-Golgi network and vesicles in the cell periphery. Therefore, TF FOS with the NAA16-induced acetylation and USP31-induced ubiquitination positively regulates gene *MMP12* to result in the degradation of extracellular matrix (ECM). TF JUN with the PPP3CA-induced phosphorylation and USP34-induced methylation positively regulates gene *PPARD* to produce ROSs to eliminate *C. albicans* WO-1. Moreover, receptor HSP90B1 affected by the GAPDHP24-induced phosphorylation binds to proteins TMEM205, which is related to chemotherapeutic agent, to trigger TF JUN. Previous studies indicate that after accepting chemotherapeutic, *C. albicans* WO-1 will invade host cell again and recover itself in immune-compromised patients [44]. In addition, the receptor ERBB2 triggers TF GATA1 through a sequence of signaling proteins RER1 to regulate ER protein, HIST1H4B which is influenced by the UBE2J2-induced ubiquitination and SSH2-induced phosphorylation-involved histone binding, SSR4, GRB2 which could influence cell death, UCN2, SH2D1A which is related to immune regulatory interactions, RAB12 involved in autophagy modulation, and EED. Finally, comparing to *C. albicans* SC5314, the receptor EGFR with the NAALADL2-induced acetylation and OTUD3-induced ubiquitination then activates downstream protein AVEN to modulate TF YBX1. The TF YBX1, which is influenced by the OTUD3-induced ubiquitination and TPMTP2-induced methylation, positively regulates innate immune-related gene *UCN2*. TF GATA1 negatively regulates gene *AVEN* to eliminate *C. albicans* WO-1 by autophagy. However, MiR-30B represses gene *AVEN* to reduce autophagy function. Then, due to gene *MMP12* being regulated to result in ECM degradation such as collagen, vitronectin, laminin and fibronectin, *C. albicans* WO-1 starves to nutrient or carbon sources. 

*C. albicans* WO-1 begins to grow hyphae and invade host cell. CAWG_2005 (ALS3) triggers TF CAWG_02083 (EFG1) and CAWG_01948 (BCR1) by directly binding to these TFs. The TF CAWG_02083 (EFG1) positively regulates gene *CAWG_2005* (ALS3) so that *C. albicans* can invade host cell and strengthen cell adhesion to anchor cell. The TF CAWG_01948 (BCR1) can positively regulate gene *CAWG_03451* (HWP1) which is related to biofilm forming and cell adhesion. In addition, pathogen receptor CAWG_03451 (HWP1) can activate downstream pathogen protein CAWG_04849 (SFL2), which could regulate *C. albicans* morphogenesis through signaling protein CAWG_01914 (SFL1) for triggering TF CAWG_02083 (EFG1); the pathogen receptor HWP1 can also trigger signaling protein CAWG_04944 (FLO8), which could influence on hyphal formation to modulate TF CAWG_02083 (EFG1), CAWG_01948 (BCR1) and CAWG_00682 (CPH1). The TF CAWG_00682 (CPH1) also positively regulates hyphae growth-related gene *CAWG_01560* (RSR1). According to previous researches, yeast cell of *C. albicans* could damage macrophages via hyphae growth to reduce destruction of *C. albicans* [45].

In conclusion, *C. albicans* SC5314 and WO-1 infect host cell at the beginning, then immune system of host cell can defense it. Although hyphae of *C. albicans* can destruct macrophage, host cells combine other cellular mechanisms to resist it such as autophagy, ROS production and immune response. At this moment, *C. albicans* SC5314 and WO-1 could be considered as a commensal pathogen of adhesion stage at the host cell. Furthermore, in different strains, gene expressions of *MMP12* and *PPARD* under the infection of *C. albicans* SC5314 are higher than *C. albicans* WO-1. By contrast, gene expression of *AVEN* under the infection of *C. albicans* SC5314 is lower than *C. albicans* WO-1. From experiment data, gene *PPARD* has a significant change of expression in the infection progression of both strains. As a result, the degradation intensity of *C. albicans* SC5314 is stronger than that of *C. albicans* WO-1. Relatively, ROS production in the infection of *C. albicans* SC5314 is also more powerful than *C. albicans* WO-1. Hence, *C. albicans* WO-1 is easily eliminated by regulating the expressing of host gene *AVEN*. In addition, the expression levels of CDH1 and ERBB2 under the infection of *C. albicans* SC5314 are higher than *C. albicans* WO-1. In this situation, the infection of *C. albicans* SC5314 has induced a relatively strong endocytosis and epigenetic modification. For the infection of *C. albicans* WO-1, it will bring about more protein folding of host cell by higher expression of HSP90B1 with phosphorylation. Namely, compared to *C. albicans* SC5314, host cell with infection of *C. albicans* WO-1 leads to a more misfolded protein formation.

### 3.2. OKF6/TERT-2 Cell Confronts Different Strains of C. albicans by Strong ROS and Microenvironment Response

**Figure 4:**
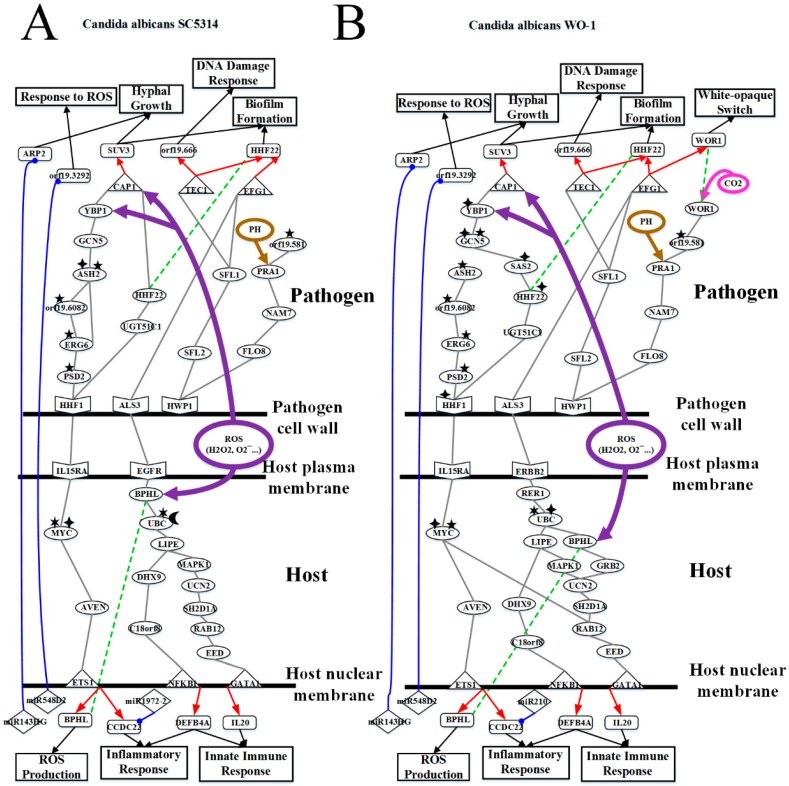
The continuous ROS and stress production as defense mechanism in host cells, and the corresponding anti-ROS and offensive mechanism of *C. albicans*. (**A**) OKF6/TERT-2 cells antagonize in *C. albicans* SC5314 invasion. (**B**) OKF6/TERT-2 cells antagonize in *C. albicans* WO-1 invasion. The red arrow lines represent transcriptional regulation; the grey solid lines signify the protein–protein interaction; the green dot lines indicate the protein translation; the blue lines with circle endpoint represent miRNA repression. The circles with purple frame and arrow lines represent the production activity and response of ROS. In (**A**), because more *C. albicans* SC5314 invade gradually, host cell continues ROS production and increases stress on *C. albicans* SC5314. Therefore, *C. albicans* SC5314 needs to execute DNA damage response and resist ROS. Next, *C. albicans* SC5314 performs hyphae growth function to form biofilm continually. Due to hyphal growth, host cells are oppressed to cause inflammation response and cellular stress. At this time, OKF6/TERT-2 cells stay at an unbalance status. In (**B**), because more *C. albicans* WO-1 invades gradually, host cell continues ROS production and increases stress. Therefore, *C. albicans* WO-1 need to execute DNA damage response and resist ROS. Next, *C. albicans* WO-1 performs hyphae growth function to form biofilm continually. Due to hyphal growth, host cells are oppressed to cause inflammation response and cellular stress. At this time, OKF6/TERT-2 cells stay at an unbalance status. Eventually, *C. albicans* WO-1 senses carbon dioxide to transform white cell.

As shown in Figure 4A, due to the degradation of extracellular matrix, *C. albicans* proceeds to invasion stage, and host cell membrane begins to be destructed by the hyphal of *C. albicans* SC5314. More and more ROSs are generated by host cell. In Figure 4A, pathogen cell surface protein orf19.1816 (ALS3) binds to the host cell receptor EGFR so that the receptor EGFR will be degraded and induced in the ROS production of the host cell. After receiving the corresponding signal, the receptor EGFR triggers TFs NFKB1 (subunit of NF-κB) and GATA1 to regulate biological oxidations and hydrolase activity through a sequence of signaling proteins BPHL, UBC with the OTUB1-induced ubiquitination and INPP4B-induced phosphorylation, LIPE involved in lipid metabolism and bound to DHX9 and MAPK1 (also known as p38, ERK, PRKM1), DHX9 which could mediate TLR4 signal and NF-kB activation, MAPK1 which participates with EGFR-related signaling pathway to regulate cell survival and differentiation, UCN2 mentioned above, SH2D1A referred to previous section, RAB12 mentioned above, EED and C18orf18. The TF GATA1 positively regulates an immune-related gene *IL20* to recruit more macrophage and pro-inflammatory cytokine. Another TF NFKB1 negatively regulates fungus infection-related gene *DEFB4A*. Since *C. albicans* is a Gram-positive fungus, gene *DEFB4A* negatively regulated by TF NFKB1 results in resisting pathogen invasion and inflammation. In addition, some of *C. albicans* SC5314 still stay at cell surface because they would like to form colony morphology by hyphal growth and yeast cell. However, pathogen membrane protein orf19.1059 (HHF1), which is related to colony morphology, interacts with the host cell receptor IL15RA. After the receptor receives the signaling, IL15RA binds to MYC. MYC with NAALADL1-induced acetylation and PDPR-induced phosphorylation activates downstream signaling protein AVEN for triggering the TF ETS1. The TF ETS1 positively regulates genes *BPHL* mentioned previously and *CCDC22* involved in the activation of pro-inflammatory NF-κB signaling. Therefore, genes *CCDC22* and *DEFB4A* will generate inflammatory in response to the regulation of TFs ETS1 and NFKB1, respectively. It is noted that inflammatory response in the invasion phase will not be over reaction due to the repression of miR1979-2 on gene *CCDC22*.

Afterwards, pathogen starts to antagonize the immune system of the host cell. After orf19.1059 (HHF1) interacting with the host cell receptor IL15RA, it will trigger TF orf19.1623 (CAP1) through a sequence of downstream proteins, orf19.3954 (PSD2) with the orf19.1631-induced methylation, orf19.1631 (ERG6) affected by orf19.3964-induced methylation and bound to orf19.3964 directly, orf19.6082 influenced by orf19.169-induced (CHO2) methylation, orf19.3964 (ASH2) with the orf19.1631-induced methylation and orf19.705-induced acetylation, orf19.705 (GCN5) and orf19.5034 (YBP1) stabilizing TF orf19.1623 (CAP1) and reacting to oxidative stress. Another orf19.1059-related (HHF1) pathway binds to orf19.1854 (HHF22) through interacting with orf19.2616 (UGT51C1) to modulate TF orf19.1623 (CAP1). Orf19.1631 (ERG6) protein is related to drug resistance such as azole-resistance and induction of fluconazole by own methyltransferase. The pathogen TF orf19.1623 (CAP1) positively regulates hyphae growth and biofilm-related gene *orf19.4519* (SUV3) to confront with ROS producing by host cell because *C.albican* SC5314 could form colony at host cell surface. Moreover, orf19.1816 (ALS3) interacts with the TF orf19.610 (EFG1) so that orf19.610 can positively regulate biofilm and drug resistance-related of gene *orf19.1854* (HHF22). Finally, pathogen receptor orf19.1321 (HWP1) activates TFs orf19.5908 (TEC1) through a sequence of signaling proteins, orf19.3969 (SFL2) and orf19.454 (SFL1) which also triggers TF orf19.610 (EFG1). The TF orf19.5908 (TEC1) positively regulates gene *orf19.666* and negatively regulates *orf19.1854* (HHF22), respectively. Due to host defense mechanisms and the microenvironment, orf19.666 will induce DNA damage response which *C. albicans* SC5314 will have DNA repair by itself. However, orf19.1321 (HWP1) can execute some cellular functions without activating TFs. Affected by the microenvironment, pathogen receptor orf19.1321 (HWP1) binds to orf19.939 (NAM7) via orf19.1093 (FLO8) to stimulate orf19.3111 (PRA1) resulting in hyphae growth directly by neutral and alkaline pH. Previous study suggests that hyphal can grow and elongate in neutral and alkaline pH condition [43]. Therefore, orf19.3111 (PRA1) will promote the elongation of hyphae to form the biofilm. Moreover, orf19.3111 (PRA1) is not only influenced by microenvironment but also activated by orf19.581 with orf19.2575-induced methylation to increase hyphal growth. Since orf19.3292 is a peptide-methionine (R)-S-oxide reductase, it will combine with thioredoxin to decrease ROS concentration. It gives rise to the host miRNA548D2 inhibiting *orf19.3292* to strengthen ROS and eliminate pathogen. Moreover, orf19.7292 (ARP2) is composed of Arp2/3 complex required for virulence, hyphal growth and cell wall/cytoskeleton organization. Facing this situation, host miRNA143HG silences gene *orf19.7292* (ARP2) to reduce hyphae growth and further prevent the forming of biofilm. Eventually, both host defense mechanisms and host-miRNAs withstand the invasion of *C. albicans* SC5314.

As shown in Figure 4B, due to the degradation of extracellular matrix, *C. albicans* proceeds to the invasion stage, and the host cell membrane begins to be destructed by the hyphal of *C. albicans* WO-1. More and more ROSs are generated by the host cell. In Figure 4B, pathogen cell surface protein CAWG_2005 (ALS3) binds no longer to EGFR but to host cell receptor ERBB2 making receptor ERBB2 degrade and induce in ROS production. After receiving the corresponding signal, the receptor ERBB2 triggers TFs NFKB1 (subunit of NF-κB) and GATA1 through a sequence of signaling proteins, RER1, UBC with the NAALADL2-induced acetylation and PPP4R4-induced phosphorylation, BPHL regulating biological oxidations and hydrolase activity and the binding to GRB2, LIPE involving in lipid metabolism and binding to DHX9 and MAPK1 (also known as p38, ERK, PRKM1), DHX9 mediating TLR4 signal and NF-kB activation, MAPK1 participating in the EGFR-related signaling pathway and regulation of cell survival and differentiation, UCN2 accepting signals from GRB2 and MAPK1, SH2D1A referring to the previous section, RAB12 having been mentioned above, EED and C18orf18. Compared to *C. albicans* SC5314, receptor ERBB2 instead of receptor EGFR activates BPHL-related pathway to strengthen ROS production via UBC adjusted by epigenetic modifications.

The TF GATA1 positively regulates the immune-related gene *IL20* to recruit more macrophage and pro-inflammatory cytokine. Another TF NFKB1 negatively regulates fungus infections-related gene *DEFB4A*. Since *C. albicans* are Gram-positive fungus, gene *DEFB4A* negatively regulated by TF NFKB1 can result in resisting pathogen invasion and inflammation finally. In addition, some of *C. albicans* WO-1 still stay cell surface because they would like to form colony morphology by hyphal growth. However, pathogen membrane protein CAWG_00969 (HHF1), which is related to colony morphology, interacts with the host cell receptor IL15RA. After the receptor receives the signaling, IL15RA binds to signaling protein MYC with the ELP6-induced acetylation and MBD5-induced methylation to activate downstream protein AVEN and interact with RAB12 for triggering TFs ETS1 and GATA1. TF ETS1 positively regulates gene *BPHL* which has mentioned previously and negatively regulates *CCDC22*, involved in the activation of pro-inflammatory NF-κB signaling. Therefore, genes *CCDC22* and *DEFB4A* will generate inflammatory response through the regulation of TFs ETS1 and NFKB1, respectively. Nonetheless, the inflammatory response in this phase will not be over reaction due to the repression of miR210 on gene *CCDC22*. Compared to *C. albicans* SC5314, MYC regulated by methylation instead of phosphorylation can activate RAB12 for triggering downstream TFs.

Afterwards, pathogen begins to antagonize the immune system of host cell. After CAWG_00969 (HHF1) with CAWG_03659-induced (NAT4) acetylation interacting with the receptor IL15RA, it will trigger TF CAWG_02548 (CAP1) through downstream signaling proteins CAWG_01562 (UGT51C1), CAWG_01979 (HHF22) with CAWG_03824-induced acetylation, CAWG_03824 (SAS2) with CAWG_1970-induced acetylation, CAWG_01970 (GCN5) with CAWG_03824-acetylation and CAWG_01059 (SPP1) methylation, and CAWG_00057 (YBP1) which is influenced by CAWG_01970-induced acetylation to stabilize TF CAWG_02548 (CAP1) and react to oxidative stress so that it will grow hyphae. Compared to *C. albicans* SC5314, another CAWG_00969-related (HHF1) pathway cannot trigger TF CAWG_02548. In addition, both CAWG_01970 (GCN5) and CAWG_00057 (YBP1) are influenced by acetylation to strengthen the modulation of TF CAWG_02548 (CAP1) to induce hyphae growth or elongation. Next, our results indicate that CAWG_00969 (HHF1) through the following signaling proteins, CAWG_04836 (PSD2) with CAWG_02542-induced methylation, CAWG_02542 (ERG6) with CAWG_05150-induced methylation (SWD3), CAWG_01344 (orf19.6082) with CAWG_01594-induced methylation (CHO2), and CAWG_04844 (ASH2) with CAWG_01059-induced methylation (SPP1), are not enough to interact with downstream protein CAWG_1970 (GCN5). We can infer that CAWG_04844 (ASH2) may not receive enough epigenetic modifications or other protein activations but protein CAWG_02542 (ERG6) is still related to drug resistance by own methyltransferase and cannot influence drug resistance of endurance. In addition, the influence of ROS will be decreased by the repression of CAWG_04844 (ASH2). The TF CAWG_02548 (CAP1) negatively regulates hyphae growth and biofilm-related gene *CAWG_04191* (SUV3) to confront with ROS production by host cell because *C.albican* WO-1 could form colony at cell surface. Moreover, CAWG_02005 (ALS3) interacts with TF CAWG_02083 (EFG1) so that CAWG_02083 (EFG1) can positively regulate the biofilm and drug resistance-related gene *CAWG_01979* (HHF22), and the main activation of white-to-opaque switch of gene *CAWG_00418* (WOR1). Finally, pathogen receptor CAWG_03451 (HWP1) activates TFs CAWG_02766 (TEC1) through signaling proteins, CAWG_04849 (SFL2) and CAWG_01914 (SFL1) which also triggers TF CAWG_2083 (EFG1). The TF CAWG_02766 (TEC1) positively regulates gene *CAWG_00299* (orf19.666). Due to host defense mechanisms and the microenvironment, *CAWG_00299* (orf19.666) will generate DNA damage response so that *C. albican* WO-1 will progress DNA repair by itself. However, CAWG_03451 (HWP1) can execute some cellular functions without activating TFs. Affected by the microenvironment, pathogen receptor CAWG_03451 (HWP1) binds to CAWG_04444 (NAM7) via interacting with CAWG_04944 (FLO8) to stimulate CAWG_03130 (PRA1) resulting in hyphae growth directly by pH. Previous study suggests that hyphal can grow and elongate in neutral and alkaline pH condition [43]. Therefore, CAWG_03130 (PRA1) will promote elongation hyphae to form biofilm. Moreover, CAWG_04472 (orf19.581) with the CAWG_01529-induced methylation simultaneously increases expression level of CAWG_00418 (WOR1) and CAWG_03130 (PRA1) for triggering hyphal growth and white-to-opaque switch. Nonetheless, CAWG_00418 (WOR1) will not only be influenced by CO_2_-induced or acidic microenvironment but also be activated by protein CAWG_04944 (FLO8) indirectly which is also related to CO_2_ inducing white-opaque switch and virulence. Since *CAWG_01270* (*orf19.3292*) is a peptide-methionine (R)-S-oxide reductase, it will be combined with thioredoxin to decrease ROS concentration. It gives rise to the host miRNA548D2 inhibiting *orf19.3292* to strengthen ROS and eliminate pathogen. Moreover, orf19.7292 (ARP2) is composed of Arp2/3 complex required for virulence, hyphal growth and cell wall/cytoskeleton organization. Facing this situation, host miRNA143HG silences gene *CAWG_02173* (ARP2) to reduce hyphae growth and further prevent the forming of biofilm. Eventually, both host defense mechanisms and host-miRNAs withstand *C. albicans* WO-1 invasion.

In conclusion, in the invasion phase, the common pathogenic mechanism of *C. albicans* SC5314 and *C. albicans* WO-1 is hyphae growth and elongation to further form biofilm. In addition, *C. albican* SC5314 and *C. albicans* WO-1 can resist ROS and immune cells such as macrophages and neutrophils. However, host cell stays at an unstable condition between acidic and alkaline pH. Because of lipid metabolism and ROS response, lipid depletion causes CO_2_ production. Additionally, overreaction of ROS also produces CO_2_ and hydrogen ion generating acidic substance. With these materials, host cell becomes acidic gradually. Under this microenvironment, *C. albicans* WO-1 exploits these materials to switch white cell. However, acidic substance production represses hyphal growth rather than to activate. Overall, host cell has a need to be balance between acidic and neutral pH. Along with more and more hyphae to oppress the host cell and penetrate cytoplasm, host cell generates inflammatory response gradually to recruit more cytokines to eliminate *C. albican*. At this moment, *C. albicans* still would be eliminated because of immature biofilm. Moreover, according to the experimental data, gene expressions of *BPHL* and *DEFB4A* of *C. albicans* SC5314 are higher than *C. albicans* WO-1. By contrast, gene expression of *IL20* of *C. albicans* SC5314 is lower than *C. albicans* WO-1. In addition, gene *IL20* has a significant change of expression in the initial infection. Therefore, the innate immune response also plays an important role in this stage so that *C. albicans* has to be perished. However, under *C. albicans* SC5314, host cell is easier to resist pathogen by the host gene expression. On the contrary, *C. albicans* WO-1 is difficult to be antagonized so that it will invade host cell rapidly. Additionally, the expression of IL15RA in the *C. albicans* WO-1 condition is higher than *C. albicans* SC5314 from the experimental data. It suggests that *C. albicans* WO-1 simply binds to IL15RA so that *C. albicans* WO-1 quickly forms cell colony. As a result, *C. albicans* WO-1 is not easier to be wiped out. Although host cell is anxious to rub out *C. albicans*, it will stay at an unbalance state such as pH changing and CO_2_ production. Hence, pathogen protein orf19.3111 (PRA1) will induce hyphal growth even in different strains. From the experimented data, orf19.3111 (PRA1) has a significant change of expression in the infection.

### 3.3. Released Pathogenic Factor and Accumulated Cellular Response Result in Apoptosis and Inflammatory Response Further Leading to Necrosis 

**Figure 5:**
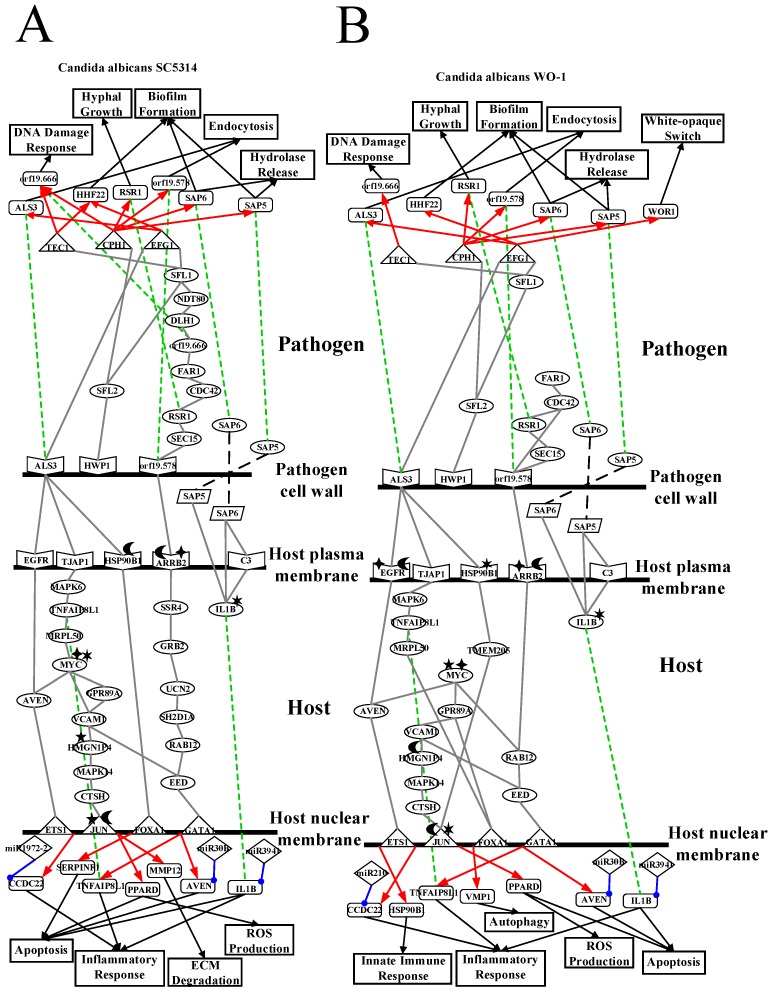
*C. albicans* could release pathogenic factor and the accumulated cellular stress in host cell could result in apoptosis and inflammatory response leading to necrosis. The red arrow lines represent transcriptional regulation; the grey solid lines signify the protein–protein interaction; the green dot lines indicate the protein translation; the blue lines with circle endpoint represent miRNA repression. In (**A**), *C. albicans* SC5314 brings about numerous invasions, and host cells generate more and more inflammatory response via hyphal elongation. Following this, hyphae generate oppression on host cell so that host cell triggers apoptosis function of host cell. *C. albicans* SC5314 releases pathogenic factor to further involve in inflammatory response and apoptosis. Due to ER stress on the whole host cell, it influences the risk of the survival of host cell. Nonetheless, *C. albicans* SC5314 will form biofilm easily and enforce sexual reproduction so that it will output more yeast cells in the host cell. Next, yeast cells can colonize or invade other host cells. In (**B**), *C. albicans* WO-1 brings about numerous invasions, and host cells generate more and more inflammatory response via hyphal elongation. Following this, hyphae generate oppression on host cell so that host cell triggers apoptosis function of the host cell. *C. albicans* releases pathogenic factor to further involve in inflammatory response and apoptosis. Due to ER stress on the whole host cell, it influences the risk of the survival of host cell. Nonetheless, *C. albicans* WO-1 will form biofilm easily and enforce sexual reproduction so that it will output more yeast cells in the host cell. Next, yeast cells can colonize or invade other host cells. Finally, the whole microenvironment can produce more another cell type such as opaque cell because the whole host cell stays at anaerobic or acidic condition via the last response. Therefore, *C. albicans* WO-1 yields more opaque cells.

As shown in Figure 5A, due to numerous invasions of *C. albicans* SC5314 and over ROS production, cellular stress becomes larger gradually. Because of overfull ROS, host cell leads to producing a more inflammatory response. From pathogen cell surface protein orf19.1816 (ALS3) binding to these host receptors EGFR, TJAP1 and HSP90B1, these receptors will finally cause apoptosis and inflammatory response. Firstly, the receptor EGFR triggers TF ETS1 via signaling protein AVEN which is modulated by MYC with the NAALADL1-induced acetylation and PDPR-induced phosphorylation. TF ETS1 positively regulates gene *CCDC22*. Secondly, the receptor TJAP1 (also knowns as TJP4) activates TFs ETS1, JUN and GATA1 through a sequence of signaling proteins, MAPK6 (also known as ERK3), TNFAIP8L1, MRPL50, which is related to the maintenance of cell organelle, MYC, which is affected by the NAALADL1-induced acetylation and PDPR-induced phosphorylation and can activate downstream three proteins such as VCAM1, AVEN and GPR89A interacting with VCAM1 and reducing intracellular pH, HMGN1P4 with the KDM4A-induced methylation, MAPK14, CTSH, and EED. The TF JUN with the USP12PX-induced ubiquitination and PRMT1-induced methylation negatively regulates gene *PPARD* to influence ROS production and apoptosis and gene *MMP12* to influence ECM degradation. The TF GATA1 positively regulates the inflammation-related gene *TNFAIP8L1* and apoptosis-related gene *AVEN*, respectively. Eventually, the receptor HSP90B1 with USP11-induced ubiquitination interacts with TF FOXA1 directly. The TF FOXA1 negatively regulates apoptosis-related gene *SERPINF1*. In addition, another pathway also activates TF GATA1 because of ARRB2. When the pathogen membrane protein orf19.578 binds to the receptor ARRB2, which is affected by the HDAC1-induced acetylation and HERC5-induced ubiquitination, ARRB2 then activates TF GATA1 via the signaling pathway including SSR4, GRB2 which could induce cell death, UCN2, SH2D1A, RAB12 and VCAM1-activating EED. Not only these receptors but also complement system of receptor C3 (also known as C3a and C3b) will induce inflammatory response. Since *C. albicans* SC5314 releases proteases orf19.5542 (SAP6) and orf19.5585 (SAP5), C3 will be degraded by orf19.5542 (SAP6) and trigger IL1B (also known as IL-1β). IL1B is affected by the PPP2CA-induced phosphorylation and bound to both orf19.5542 (SAP6) and orf19.5585 (SAP5) directly resulting in inflammatory response and cell apoptosis.

Afterwards, as a result of over inflammatory reaction and cellular stress, miRNAs have to repress these responses. miR1979-2 and miR3941 inhibit genes *CCDC22* and *IL1B*, respectively. Next, miR-30B represses *AVEN* to reduce apoptosis. Nevertheless, at this moment, *C. albicans* SC5314 invades continuously. Orf19.1816 (ALS3) binds to TF orf19.610 (EFG1) directly so that *C. albicans* SC5314 carries out cellular functions quickly such as biofilm formation and endocytosis. The TF orf19.610 (EFG1) positively regulates the endocytosis-related gene *orf19.1816* (ALS3) and biofilm-related gene *orf19.1854* (HHF22). Additionally, orf19.1321 (HWP1) interacts with orf19.3969 (SFL2) to modulate TF orf19.4433 (CPH1). Then, TF orf19.4433 (CPH1) positively regulates the endocytosis-related gene *orf19.578*, DNA damage-related gene *orf19.666*, hydrolytic activity and biofilm-related genes *orf19.5542* (SAP6) and *orf19.5585* (SAP5) but negatively regulates the hyphal growth-related gene *orf19.2614* (RSR1). Therefore, *C. albicans* SC5314 can invade or damage host cell considerably. Apart from this, a pathogen membrane protein orf19.578 triggers TF orf19.5908 (TEC1) through a sequence of downstream signaling proteins orf19.1418 (SEC15) involved in the hyphae branch growth, orf19.2614 (RSR1) featuring the same as orf19.1418 (SEC15), orf19.390 (CDC42) keeping the hyphal growth and Rho-type GTPase activity, orf19.7105 (FAR1), orf19.666, orf19.3760 (DLH1), orf19.2119 (NDT80) for the wild-type drug resistance, and orf19.454 (SFL1) receiving orf19.3969 (SFL2) to regulate morphogenesis function through the activation of TFs orf19.610 (EFG1) and orf19.5908 (TEC1). TF orf19.5908 (TEC1) negatively regulates genes *orf19.1854* (HHF22), which is involved in forming biofilm, and *orf19.666*. However, due to numerous invasions of *C. albicans* SC5314 and biofilm formation, *C. albicans* SC5314 executes DNA repair easily via orf19.666 regulated by two TFs to recover chromosome of *C. albicans*. 

Through these cellular functions especially biofilm formation, *C. albicans* SC5314 finally causes apoptosis and cell necrosis. By the hyphal of *C. albicans* SC5314, it will actively penetrate and destroy mitochondrial or nuclear. Following the process, hyphae will cross each other and then form biofilm. No matter where biofilm forms outside or inside the host cell membrane, biofilm will lead to cell necrosis and stress response because host cell covered by *C. albicans* SC5314 stays at the hypoxia and acidic condition. Therefore, *C. albicans* SC5314 prefers sexual reproduction and not be subject to drug control. Previous study mentioned that biofilm of *C. albicans* could antagonize drug so that it cannot be eliminated and yields drug resistance [46]. Similarly, ROS production will not eliminate *C. albicans* because previous study indicated that biofilm formation could against it easily [47]. Therefore, oxidative stress via generating ROS will be harmful to the host cell. Not only producing anti-drug pathogen but also switching white cell to opaque cell generate more yeast cell by forming biofilm. Consequently, the host cell accumulates much cell stress causing cell apoptosis. Moreover, due to destruction of the host cell, inflammatory response will cause necrosis by hyphae.

As shown in Figure 5B, under numerous invasions of *C. albicans* WO-1 and over ROS production, cellular stress becomes larger gradually. Because of the overfull ROS, host cell leads to producing a more inflammatory response. As the pathogen cell surface protein CAWG_02005 (ALS3) binds to host cell membrane receptors EGFR, TJAP1, HSP90B1 and ARRB2, these host receptors will finally cause apoptosis and inflammatory response. Firstly, receptor EGFR with the NAALADL2-induced acetylation and OTUD3-induced ubiquitination triggers TF ETS1 via signaling protein AVEN that is also modulated by MYC, which is influenced by the ELP6-induced acetylation and MBD5-induced methylation. TF ETS1 negatively regulates genes *CCDC22* and *HSP90B1*. By contrast, receptor HSP90B1 will bring about folding protein and immune response under the *C. albicans* WO-1 infection. Secondly, the receptor TJAP1 (also knowns as TJP4) activates TF FOXA1 through a sequence of signaling proteins, MAPK6 (also known as ERK3), TNFAIP8L1, and MRPL50 related to the maintenance of cell organelle. The TF FOXA1 positively regulates the autophagy-related gene *VMP1*. However, compared to *C. albicans* SC5314, autophagy function will increase cellular stress at this phage. Eventually, the receptor HSP90B1, which is affected by the GAPDHP24-induced phosphorylation, triggers TF JUN through the signaling protein TMEM205. The TF JUN with the PPP3CA-induced phosphorylation and USP34-induced methylation positively regulates the apoptosis-related gene *PPARD*. Finally, as CAWG_04469 (orf19.578) binds to the receptor ARRB2, ARRB2 with the HGSNAT-induced acetylation and USP41-induced ubiquitination can activate TF GATA1 through proteins RAB12 involved in autophagy modulation, and EED in the signal pathway. Compared to *C. albicans* SC5314, receptor ARRB2 triggers TF GATA1 with less signaling proteins since it will be influenced by stronger epigenetic modification. The TF GATA1 negatively regulates the apoptosis-related gene *AVEN* and inflammatory-related gene *TNFAIP8L1*, respectively. In addition, another pathway also activates TFs JUN, FOXA1 and GATA1. MYC with epigenetic modifications activates TFs JUN, FOXA1 and GATA1 through signaling proteins GPR89A activating TFs FOXA1 and connecting to VCAM1, which activates TF GATA1 via binding EED, HMGN1P4 with the USP25-induced ubiquitination, MAPK14 and CTSH. In Figure 5B, MYC with methylation activates to interact with RAB12 under the infection of *C. albicans* WO-1 which is different from *C. albicans* SC5314. Not only these receptors but also complement system of receptor C3 (also known as C3a and C3b) will induce an inflammatory response. Since *C. albicans* WO-1 releases proteases CAWG_05066 (SAP5) and CAWG_05098 (SAP6), C3 will be degraded by CAWG_05098 (SAP6) and triggers IL1B (also known as IL-1β). IL1B is influenced by the PPP1R15A-induced phosphorylation and bound to both CAWG_05066 (SAP5) and CAWG_05098 (SAP6) resulting in inflammatory response and cell apoptosis. 

Afterwards, as a result of over inflammatory reaction and cellular stress, miRNAs have to repress inflammatory response. The only difference about miRNAs in the infection of *C. albicans* WO-1 is characterized by mir210 silencing *CCDC22*. At this moment, host cell decreases apoptosis but *C. albicans* WO-1 invades continuously. CAWG_02005 (ALS3) binds to TF CAWG_02083 (EFG1) directly so that *C. albicans* WO-1 carries out cellular functions quickly such as biofilm formation and endocytosis. The TF CAWG_02083 (EFG1) positively regulates the endocytosis-related gene *CAWG_02005* (ALS3), biofilm-related gene *CAWG_01979* (HHF22), and white-to-opaque switch main gene *CAWG_00418* (WOR1). Additionally, receptor CAWG_03451 (HWP1) modulates TFs CAWG_00682 (CPH1), CAWG_02083 (EFG1) and CAWG_02766 (TEC1), which are mediated by proteins CAWG_04849 (SFL2) and CAWG_01914 (SFL1). Then, TF CAWG_00682 (CPH1) positively regulates the hyphal growth-related gene *CAWG_01560* (RSR1), hydrolytic activity and biofilm-related genes *CAWG_05098* (SAP6) and *CAWG_05066* (SAP5) but negatively regulates the endocytosis-related gene *CAWG_04469* (orf19.578). By contrast, TF CAWG_00682 (CPH1) does not regulate the DNA damage-related gene *CAWG_00299* (orf19.666) under the infection of *C. albicans* WO-1. We infer that the reason why CAWG_00682 (CPH1) being inactivated is indirectly caused by cell cycle proteins such as CAWG_05375 (FAR1) and CAWG_03794 (NDT80). Since these proteins may execute functions in the white cell, *C. albicans* WO-1 transforms opaque cells to further reduce protein expression. The TF CAWG_02766 (TEC1) positively regulates the DNA damage-related gene *CAWG_00299* (orf19.666) continuously. Compared to *C. albicans* SC5314, TF CAWG_02766 (TEC1) does not regulate gene *CAWG_1979* (HHF12). Perhaps, white cells of *C. albicans* WO-1 mostly transform to opaque cells so that TF CAWG_02766 (TEC1) could reduce the modulation of *CAWG_1979* (HHF12). Hence, *C. albicans* WO-1 can invade or damage host cell by different types of *C. albicans* largely. However, compared to *C. albicans* SC5314, CAWG_04469 (orf19.578) does not trigger any TF. It strengthens downstream proteins to execute hyphal growth function indirectly. By interacting with CAWG_04469 (orf19.578), CAWG_03378 (SEC15) enhances the expression of gene *CAWG_01560* (*RSR1*). CAWG_00581 (CDC42) via CAWG_04469 (orf19.578) and CAWG_05375 (FAR1) also increases the gene expression of *CAWG_01560* (*RSR1*) to elongate and grow hyphae. Based on these cellular functions especially biofilm formation, *C. albicans* WO-1 finally causes apoptosis and cell necrosis. By the hyphae form of *C. albicans* WO-1, it will actively penetrate and destroy mitochondria or nuclear of the host cell. Following infection, hyphae will cross-talk each other and then form biofilm. No matter whether biofilm forms outside or inside the host cell, biofilm will cause cell necrosis because host cell covered by *C. albicans* WO-1 stays at the hypoxia and acidic condition. According to the research in Reference [3], *C. albicans* WO-1 could transform white cell to opaque easily. Therefore, compared to *C. albicans* SC5314, *C. albicans* WO-1 prefers sexual reproduction and will not be an easy subject of drug control. A previous study mentioned that the biofilm of *C. albicans* can antagonize drug so that it does not be eliminated and yields drug resistance [46]. Likely, mature biofilm will against ROS so that *C. albicans* can survive [47]. Therefore, oxidative stress via generating ROS will be harmful to the host cell. Not only producing anti-drug pathogen but also switching white cell to opaque cell can generate different cell types simply. In addition, while host membrane is covered by the biofilm formation of *C. albicans*, more cell stress and inflammatory response emerges, caused by pathogenic factor and hyphae. Ultimately, the host cell accumulates much cell stress resulting in cell apoptosis. Moreover, due to damage to the host cell, inflammatory response will cause necrosis by hyphae.

To sum up, in the host cell damage phase, *C. albicans* SC5314 and *C. albicans* WO-1 all ultimately lead to cell apoptosis and necrosis. *C. albicans* SC5314 is still hard to switch from white cell to opaque cell. Nevertheless, *C. albicans* WO-1 switches white cell to opaque cell quickly. Promoted by the microenvironment continuously, *C. albicans* SC5314 still has opportunity to switch to opaque cell. Moreover, *C. albicans* WO-1 in the opaque cell can run sexual reproduction so that it will strengthen genetic diversity and own stability. The stability of *C. albicans* WO-1 in infection progression could be described in Table 1 and Table 2. Moreover, according to the experimental data, gene expressions of *AVEN* and *TNFAIP8L1* under the infection of *C. albicans* SC5314 are lower than *C. albicans* WO-1. By contrast, the receptor expression of EGFR under the infection of *C. albicans* SC5314 is higher than *C. albicans* WO-1. In addition, protein expressions of MAPK6 and MAPK14 in *C. albicans* SC5314 are higher than *C. albicans* WO-1. Therefore, the inflammatory response and apoptosis caused by *C. albicans* WO-1 are stronger than *C. albicans* SC5314. However, under the infection of *C. albicans* SC5314, host cell is easier to resist pathogen by combining with the expression of the above mentioned proteins. On the contrary, *C. albicans* WO-1 is hard to eliminate, such that host cell needs more immune or inflammatory reaction. Furthermore, different strains of *C. albicans* could induce inflammation by releasing the pathogenic factor. According to the experimental data, orf19.5542 (SAP6) has a significant change of expression in the infection progression at different strains. Pathogen protein orf19.5542 (SAP6) is along with the expression of receptor C3 to induce inflammatory response indirectly. It is noted that the expression level of receptor C3 affected by *C. albicans* WO-1 is higher than *C. albicans* SC5314. Finally, it can be considered that *C. albicans* WO-1 will generate a stronger inflammatory response and apoptosis.

**Figure 6:**
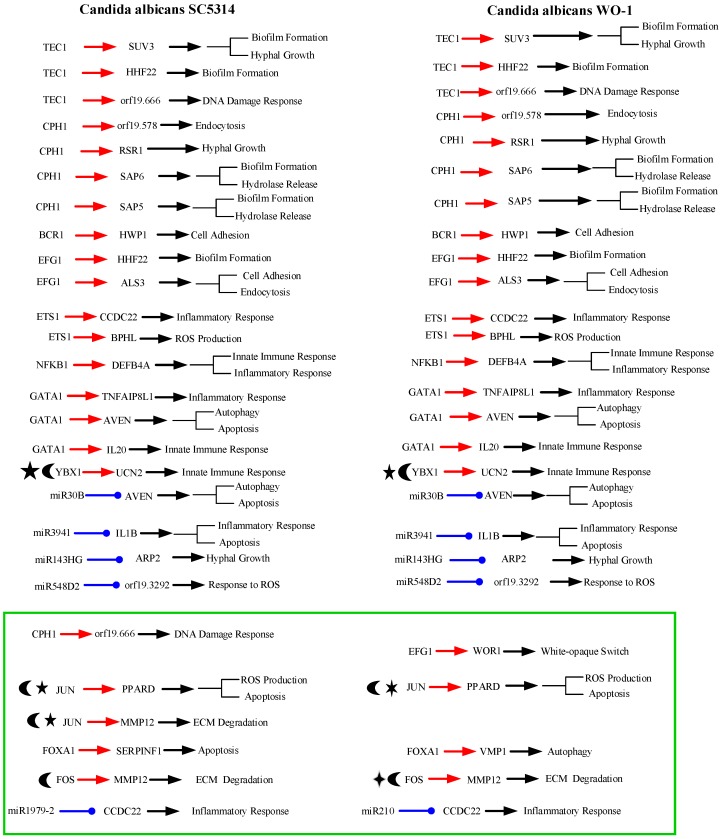
Summarizing the common and specific epigenetic and genetic pathogenic mechanisms in the infection of different strains of *C. albicans*. The figure summarizes the common and specific genetic and epigenetic pathogenic mechanisms in different strains of *C. albicans*. The green rectangular block denotes the differential regulations and functions between different strains of *C. albicans*.

Based on the discussion above, we summarize the differences in genetic and epigenetic pathogenic mechanisms during infection progression between two strains of *C. albicans* in Figure 6. Compared to *C. albicans* WO-1, *C. albicans* SC5314 holds the following differences for the infection mechanisms: (1) TF orf19.4433 (CPH1) regulates gene *orf19.666* to cause DNA damage response; (2) TF Jun with methylation and ubiquitination regulates gene *MMP12* to bring about ECM degradation; (3) TF FOS only with ubiquitination regulates gene *MMP12* to have ECM degradation; (4) TF FOXA1 regulates gene *SERPINF1* instead of VMP1 to result in apoptosis; (5) miR1979-2 rather than miR210 represses gene *CCDC22* to do inflammatory response. Compared to *C. albicans* SC5314, *C. albicans* WO-1 takes the following differences for the infection mechanisms: (1) TF CAWG_02083 (EFG1) regulates gene *CAWG_00418* (WOR1) to cause a white-opaque switch; (2) FOS with ubiquitination and methylation regulates gene *MMP12* to contribute to ECM degradation.

### 3.4. Prediction of Drug Target Proteins and the Multiple-Molecule Drug Design for the Infection of Different Strains of C. albicans

Recently, the major drugs employed to treat *C. albicans* infection include Amphotericin B, Fluconazole and Caspofungin [48]. However, *C. albicans* will generate drug resistance by forming biofilm. In addition, *C. albicans* was kept balance between host defense and fungus. As long as the balance was destroyed, *C. albicans* will invade host cell and finally produce biofilm. Following this, *C. albicans* will cause diseases such as thrush and denture-associated erythematous. It can be seen that the treatment of *C. albicans* has to prevent hyphae growth and biofilm production. Nonetheless, *C. albicans* easily leads to re-infection after accepting treatment and staying immunocompromised on chemotherapy and HIV-infected patients. Furthermore, current treatments and drugs such as Amphotericin B, Fluconazole and Caspofungin have side effects. Therefore, there is a need to find other drugs to reduce re-infection and side effects. In the meantime, since *C. albicans* WO-1 has high frequency in white-opaque switching, we have to find potential drug targets for the treatment of *C. albicans* WO-1.

According to our result, the infection of different strains of *C. albicans* with OKF6/TERT-2 cell could be used to investigate common pathogenic mechanism to predict drug targets for the design of multiple-molecule drug. We consider the important roles of orf19.1816 (CAWG_02005 in *C. albicans* WO-1 i.e., ALS3), orf19.610 (CAWG_02083 in *C. albicans* WO-1 i.e., EFG1), orf19.1321 (CAWG_03451 in *C. albicans* WO-1 i.e., HWP1), orf19.4433 (CAWG_00682 in *C. albicans* WO-1 i.e., CPH1), orf19.1623 (CAWG_02548 in *C. albicans* WO-1 i.e., CAP1) and orf19.723 (CAWG_01948 in *C. albicans* WO-1 i.e., BCR1). In these pathogen proteins, their functions include hyphae growth, endocytosis, and biofilm formation. Therefore, we can see that these pathogen TFs and pathogen cell surface proteins play a very important role in the pathogenic mechanism during the infection of *C. albicans*. Therefore, Amphotericin B, Fluconazole and Caspofungin mentioned above are still feasible drug treatments. Next, we will find other pathogen proteins as drug targets to discover new drugs according to their pathogen functions and roles in the pathogenic mechanism of *C. albicans* infection. Based on our results, the following proteins play important roles in the hyphal growth and biofilm formation: orf19.2614 (CAWG_01560 in *C. albicans* WO-1 i.e., RSR1), orf19.7292 (CAWG_02173 in *C. albicans* WO-1 i.e., ARP2), orf19.4519 (CAWG_04191 in *C. albicans* WO-1 i.e., SUV3), orf19.1854 (CAWG_01979 in *C. albicans* WO-1 i.e., HHF22), orf19.5542 (CAWG_05098 in *C. albicans* WO-1 i.e., SAP6) and orf19.5585 (CAWG_05066 in *C. albicans* WO-1 i.e., SAP5). Moreover, we also investigate other proteins involved in defense mechanism such as ROS response. Pathogen proteins about anti-ROS are orf19.1623 (CAP1), orf19.5034 (CAWG_00057 in *C. albicans* WO-1 i.e., YBP1) and orf19.3292 (CAWG_01270 in *C. albicans* WO-1). However, *C. albicans* in the infection progression exploits pathogen proteins such as orf19.7247 (CAWG_00020 in *C. albicans* WO-1 i.e., Rim101) which could exploit orf19.5585 (SAP5) for the degradation of host cell surface proteins. Eventually, the following pathogen proteins interact simultaneously with many proteins involving morphological transformation such as GTPase activity and the influence of microenvironment so that they can be considered as significant proteins to trigger TFs indirectly such as orf19.2087 (CAWG_03824 in *C. albicans* WO-1 i.e., SAS2), orf19.666 (CAWG_00299 in *C. albicans* WO-1), orf19.1093 (CAWG_04944 in *C. albicans* WO-1 i.e., FLO8) and orf19.939 (CAWG_04444 in *C. albicans* WO-1 i.e., NAM7). All the pathogen proteins mentioned above could be considered as potential common drug targets for therapeutic treatment of the infection of different strains of *C. albicans*.

**Figure 7:**
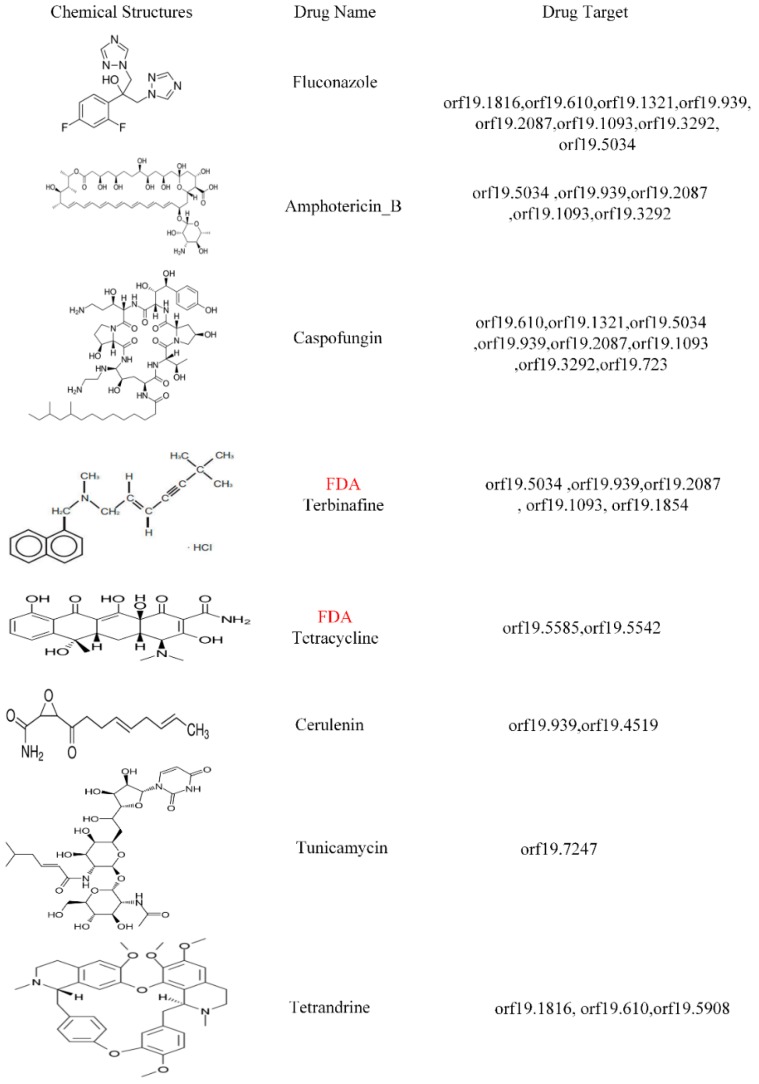
The potential common multiple-molecule drugs for the treatment of infection of different strains of *C. albicans*. At the top three drugs, Fluconazole, Amphotericin B and Caspofungin are applied to treat oral *C. albicans* infection for patients. Terbinafine can inhibit the activity of orf19.5034 (YBP1), orf19.939 (NAM7), orf19.2087 (SAS2), orf19.1093 (FLO8), and orf19.1854 (HHF22). Tetracycline can inhibit orf19.5585 (SAP5) and orf19.5542 (SAP6) of *C. albicans* from forming biofilm and releasing pathogenic factor. Cerulenin can affect the expression level of pathogen proteins orf19.939 (NAM7) and orf19.4519 (SUV3). Tunicamycin can repress pathogen orf19.7247 (RIM101) to reduce its ability of coordinating pathogen proteins for the degradation of host cell protein CDH1. Tetrandrine can inhibit orf19.1816 (ALS3), orf19.610 (EFG1) and orf19.5908 (TEC1) to reduce the ability of regulation functions of pathogen TFs. Moreover, other pathogen proteins are also applied to kinds of azole. So, these drugs are combined as multiple-molecule drugs to perish both strains of *C. albicans* simultaneously.

After suggesting these common-molecule drug targets, we explored drug databases and did literature reviews to design a multiple-molecule drug targeting different strains of *C. albicans* simultaneously. One study has demonstrated that orf19.7247 (RIM101) could induce hyphae growth and degradation of host cell receptor [49]. The other study showed that drug targets of *C. albicans* were inferred by sequence homolog between *C. albicans* and *S. cerevisiae*. Here, we can find five drugs to eliminate *C. albicans* for new potential therapy as follows. First, Tunicamycin could repress a pathogen protein orf19.7247 functioning to reduce the hyphae growth induction and coordination of pathogen proteins for the degradation of host cell protein CDH1 [50]. Second, research shows that Terbinafine can inhibit the activity of orf19.5034 and its anti-ROS ability toward the stability of pathogen TF orf19.1623 (CAP1) to eliminate pathogen initially via the ROS production [25,50]. Moreover, Terbinafine also represses the activity of orf19.1854 (HHF22) which is related to hyphae growth and biofilm formation functions. However, Terbinafine directly or indirectly influences orf19.939 (NAM7) and orf19.2087 (SAS2) so that orf19.939 and orf19.2087 could reduce the chance of triggering TFs [50]. Third, Cerulenin affects the expression levels of pathogen proteins orf19.939 and orf19.4519 (SUV3) to decrease the biofilm formation [50]. Fourth, Tetracycline could inhibit orf19.5585 (SAP5) and orf19.5542 (SAP6) so that *C. albicans* would not form biofilm to release pathogenic factor [51]. Perhaps, aspartic proteinase inhibitors could be also employed for orf19.5585 and orf19.5542 [52]. Ciclopirox olamine is also broad-spectrum antibiotics to target orf19.939, orf19.1321 (HWP1), orf19.5585, and orf19.5542 [53]. Last, Tetrandrine can play an important roles in inhibiting orf19.1816 (ALS3), orf19.610 (EFG1) and orf19.5908 (TEC1) to reduce the regulatory ability of pathogen TFs [54]. Nevertheless, previous study showed a prolonged use of broad-spectrum antibiotics could lead to an impaired immune response [55]. Therefore, we do not consider the broad-spectrum antibiotics because of an immunocompromised response causing a re-infection. Other pathogen proteins are also applied to azole compounds drug, especially Fluconazole. Nonetheless, orf19.1623 (CAP1), orf19.390 (CDC42) and orf19.578 are important human-homologs of CAPZA1, CDC42 and GRTP1, respectively. The repression of these proteins may cause unpredictable dysfunction of host cell, especially the GTPase activity and actin growth. Therefore, orf19.5034 (YBP1) is considered as a better drug target instead of orf19.1623(CAP1). Eventually, Tunicamycin, Terbinafine, Cerulenin, Tetracycline and Tetrandrine combine with the three known drugs including Fluconazole, Amphotericin B, and Caspofungin as a potential multiple-molecule drug in Figure 7. This provides an alternative way for the therapeutic treatment of two strains of *C. albicans* based on the predicted drug targets.

orf19.2614 (RSR1) and orf19.7292 (ARP2) are involved in hyphae growth and considered as important virulence factors. Moreover, orf19.666 participates in many pathogen protein interactions and DNA responses. Until now, we cannot find drugs for them. Additionally, no drug is explored for pathogen protein orf19.4884 (WOR1) regulating white-opaque switch at *C. albicans* WO-1. Therefore, we recommend that these pathogen proteins are potential drug targets for further drug design. In conclusion, our results show that orf19.2614 (RSR1), orf19.666, orf19.7292 (ARP2) and orf19.4884 (WOR1) will be significant drug targets for the design of a new common multiple-molecule drug to efficiently eliminate both *C. albicans* SC5314 and *C. albicans* WO-1.

## 4. Conclusions

The pathogenic mechanisms involved in *C. albicans* infection and resistance mechanism from host cells are complicated. For host cells, the hyphal growth and hydrolase-triggering virulence factors have been extensively investigated. However, few studies concentrate on the cross-talk mechanism between the human cell and *C. albicans*. In this study, based on big data mining, system identification, system order detection, and principal network projection methods, we investigated genetic and epigenetic interspecies networks between host OKF6/TERT-2 cells and *C. albicans* by two-sided NGS data during *C. albicans* infection. Moreover, our results distinguished the common and specific pathogenic mechanism caused by different signaling pathways to show pathogen offense and host defense in systematic viewpoint for *C. albicans* SC5314 and WO-1. The infection progression associated with essential epigenetic modifications in two strains of *C. albicans* such as ARRB2, CDH1, HSP90B1, EGFR and ERBB2, and host miRNA repressing on pathogens genes are mostly identified in core HPCNs by our systems biology approach. Furthermore, core HPCNs which obtain from applying PNP method to real GEINs are all in respect of KEGG pathways. These results indicate that epigenetic modification plays a significant role in the pathogenic mechanism for *C. albicans* SC5314 and WO-1. In the past, there are few studies discussing about the defensive mechanism in the human cell infected by *C. albicans* and the offensive mechanism of *C. albicans* in the viewpoint of pathogen epigenetic modification. A Previous study discovered epigenetic modification of *C. albicans* which did not infect the host cell [56]. Additionally, only few studies demonstrated how *C. albicans* interacts with host cell surface proteins without indicating downstream signaling proteins and target genes to generate the corresponding cellular responses. Our results show that epigenetic regulations play an important role in the common and specific pathogenic mechanism of *C. albicans*, especially *C. albicans* WO-1, transforming cell type rapidly during the infection process. This will offer a new direction for drug targets and systems drug designs. At the same time, with the recognition of core HPCNs, our understandings toward *C. albicans* infection will increase benefiting for drug discovery. In the future, the four pathogen proteins orf19.2614 (RSR1), orf19.666, orf19.7292 (ARP2), and orf19.4884 (WOR1) could be potential drug targets for the design of a new common multiple-molecule drug to eliminate both *C. albicans* SC5314 and *C. albicans* WO-1 efficiently.

## 5. Materials and Methods

### 5.1. Overview of the Construction of GEINs and Core HPCNs in OKF6/TERT-2 Cells Line during the Infection of C. albicans SC5314 and C. albicans WO-1

**Figure 8:**
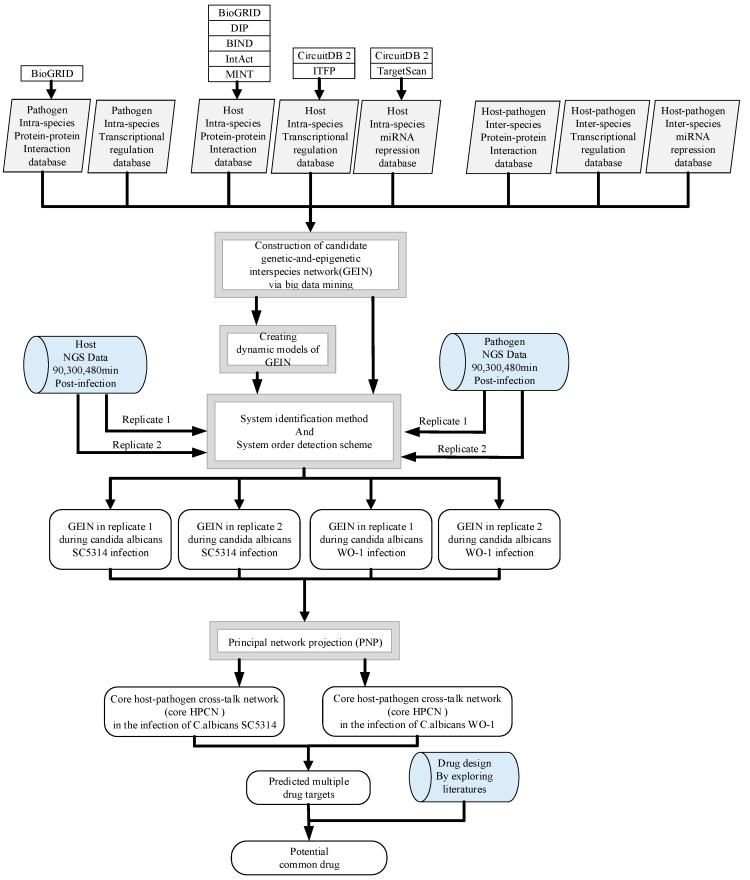
The flow chart of the systems biology method applied to construct genetic and epigenetic interspecies networks (GEINs) for extracting core HPCNs to discover the common and specific pathogenic mechanisms during the infection of *C. albicans* SC5314 and *C. albicans* WO-1 for drug targets and potential common-molecule drugs. The grey blocks indicate the big data for constructing candidate GEIN and the blue blocks represent the input information including NGS data and the surveyed literature for drug design in this study; the blocks with grey frame denote systems biology approach utilized to construct real cross-talk GEINs in the infection of *C. albicans* SC5314 and *C. albicans* WO-1, and then extract the core HPCNs of two replicates via PNP method; the white rounded rectangular blocks are our corresponding results about a common multi-molecule drug.

To investigate the common and specific pathogenic mechanisms between *C. albicans* SC5314 and *C. albicans* WO-1 during the infection of human oral epithelial OKF6/TERT-2 cells, we identified the cross-talk GEINs and extracted core HPCNs between human and *C. albicans* SC5314 as well as *C. albicans* WO-1, respectively. In Figure 8, a flowchart is given to construct core HPCNs in the state of infection by *C. albicans* SC5314 and *C. albicans* WO-1 via big data mining, dynamic model construction and network identification for investigating pathogenic mechanisms and inferring potential drug targets.

In Figure 8, we construct GEINs and HPCNs under the following steps: (1) Big data mining and data preprocessing of host/pathogen gene/miRNA expression; (2) construction of candidate GEIN, which consists of candidate host/pathogen intra-species protein–protein interaction networks (PPINs), candidate inter-species PPIN between host and pathogen, candidate host/pathogen gene/miRNA regulation networks (GRNs), candidate miRNA regulation networks of host-miRNAs on host/pathogen-genes and candidate lncRNAs regulation networks of host-lncRNAs on host-genes/pathogen-genes; (3) The network identifying process for detecting the real interspecies GEINs via system identification method and system order detection scheme, which is the Akaike’s Information Criterion (AIC) to prune false positives in the candidate interspecies GEINs by using the two-sided genome-wide NGS data of OKF6/TERT-2 cells and *C. albicans* during infection (see Sections 1.2–1.4 in Supplementary Materials); (4) The extraction of the core HPCNs by applying the principal network projection (PNP) method on the real interspecies GEINs (see Section 1.5 in Supplementary Materials). Therefore, we can project core HPCNs to KEGG pathways to obtain core cross-talk pathways and compare them to investigate the crucial common and specific pathogenic mechanisms contributing to the infection progression of *C. albicans* SC5314 and *C. albicans* WO-1, respectively. Ultimately, we infer the common network biomarkers as the potential multiple drug targets for a common multiple-drug design.

### 5.2. NGS Data Preprocessing for Human and Pathogen

To identify the cross-talk activities between host and pathogen during the infection of *C. albicans*, two strains of *C. albicans*, SC5314 and WO-1, have to infect human cell, respectively. *C. albicans* WO-1 changes its own morphology easily and can transform white cell to opaque cell. Therefore, it is difficult to control and extremely sensitive to anaerobic environment. In the previous studies, the full catalog of transcriptional changes is not completely owing to the limitations of microarrays, which lead to a limited dynamic range and poor sensitivity to analyze low-abundance transcripts. Up to now, NGS data have been obtained from the research in Reference [57], which are used to investigate the transcription profiles of both immortalized oral epithelial cells (OKF6/TERT-2 cell line) and *C. albicans*. It is the only available dataset providing sufficient information for constructing the candidate host–pathogen GEIN.

The dual NGS data studied by Liu et al. has two parts which discuss about endothelial cells and epithelial cells, respectively [57]. Here, we focus on the epithelial cells (the OKF6/TERT-2 cell line) infected by yeast-phase organisms of two different clinical isolates of *C. albicans*, SC53144 and WO-1.

The first part of NGS data includes the mRNA/miRNA expression profiles of OKF6/TERT-2 cell line at 90, 300, 480 min post-infection with *C. albicans* SC5314 and WO-1. For OKF6/TERT-2 cells, the medium was cultured in Dulbecco’s modified Eagle’s medium (DMEM) without serum at 37 °C before the cell was infected. The second part includes the mRNA expression profiles of two biological replicates of *C. albicans* SC5314 and WO-1 in OKF6/TERT-2 cells at 90, 300, 480 min post-infection (GEO accession number GSE56093; https://www.ncbi.nlm.nih.gov/geo/query/acc.cgi?acc=GSE56093). Moreover, the strain SC5314 including two replicates was originally recovered from a patient with generalized candidiasis. The other strain WO-1 including two replicates was isolated from the blood and lungs of a patient suffering from systemic candidiasis. RNA sequencing (RNA-Seq), next-generation sequencing (NGS) with llumina HiSeq 2000 platform, was used on host and two strains of *C. albicans* to provide valuable dataset for better understanding of common pathogenic mechanisms. Meanwhile, in order to avoid overfitting problem in constructing real host–pathogen GEINs which could be found in detail in Section 1.3 of the Supplementary Materials, we use cubic spline method for data interpolation within the experimental time intervals and data extrapolation after 8 h post-infection. Hence, we have two-sided NGS data from 0 min to 12 h.

### 5.3. Construction of Candidate GEINs by the Inference of Putative Interspecies and Intraspecies PPINs and GRNs for C. albicans SC5314 and WO-1

The candidate GEINs are constructed as shown in Figure 8 through big data mining from numerous databases which contain many experimental data and bioinformatics predictions. The host candidate PPIN required of protein–protein interaction (PPI) information was obtained from MINT [58,59], DIP [60], BIND [61], IntAct [59] and BioGRID [62]. The host candidate GRN required of transcription factors (TFs)/lncRNAs and their downstream-regulated genes information was obtained from CircuitDB 2 and ITFP [63,64]. The regulatory pairs of miRNAs and genes were from CircuitDB 2 and TargetScan. The candidate pathogen PPIN was obtained from BioGRID. For host–pathogen inter-species candidate PPIN and GRN and the pathogen candidate intra-species GRN, at present there is no existing database to provide sufficient information or prediction for the candidate GEIN construction. Meanwhile, the number of interactions within pathogen candidate PPIN from BioGRID is not enough to construct real GEIN. Therefore, we have to infer the putative inter-species and intra-species PPIN and GRN [65]. By utilizing the sequence homology between *C. albicans* SC5314 and *S. cerevisiae*, putative pathogen candidate intra-species PPIN could be constructed [66,67,68]. For example, in Figure S1, assume that the protein A’ and protein B’ of *S. cerevisiae* interact with each other based on databases SGD, BioGRID, String and Reactome databases [62,66,67]. Then we further identify that *C. albicans* protein A is homologous to *S. cerevisiae* protein A’; *C. albicans* protein B is homologous to *S. cerevisiae* protein B’ via sequence homology acquired from InParanoid database. Hence, we infer that protein A in *C. albicans* and protein B in *C. albicans* is a putative interspecies PPI pair. The sequence homology information for the three species, *Homo sapiens* (*H. sapiens*), *C. albicans*, *S. cerevisiae*, were from InParanoid database. Based on the same concept of sequence homology between *C. albicans* SC5314 and *H. sapiens*, host–pathogen inter-species PPIN could be constructed. In addition, we also surveyed previous studies about pathogen–pathogen intra-species PPIs [69,70,71] and host–pathogen inter-species PPIs [11,72,73,74] which could be added to increase the integrity of candidate PPIN. Similarly, utilizing InParanoid and Yeastract databases and literature survey [75,76,77,78], we could construct pathogen candidate intra-species GRN based on the concept of sequence homology between *C. albicans* and *S. cerevisiae*. On the basis of CircuitDB 2 and ITFP, we used the sequence homology between *C. albicans* SC5314 and *H. sapiens* to construct the host–pathogen candidate inter-species GRN. Finally, we utilized the sequence homology between *C. albicans* SC5314 and *H. sapiens* to construct candidate GRN of host-miRNAs targeting pathogen-genes based on databases such as TargetScan and CircuitDB 2. Moreover, the same method could be used on building candidate GEIN for *C. albicans* WO-1.

The detailed construction procedures of pathogen–pathogen candidate intra-species PPIN, host–pathogen candidate inter-species PPIN, host–pathogen candidate inter-species GRN, candidate GRN of host-miRNAs targeting pathogen-genes, pathogen-host candidate inter-species GRN, candidate GRN of pathogen-genes targeting host-miRNAs, and pathogen candidate intra-species GRN for two strains of *C. albicans* SC5314 and WO-1 are shown in Figure S1A–G. It should be noted that the putative inter-species and intra-species PPIN and GRN which we inferred were sequence homology-based method derived from various experimental conditions. In other words, false positives might exist in our candidate GEINs causing them not to reflect the actual condition of host–pathogen interaction during the individual infection on OKF6/TERT-2 cells with *C. albicans* SC5314 and WO-1. Therefore, in order to investigate the core HPCNs, system identification by solving the least-square estimation problem and system order detection method for pruning false positives (Supplementary Material Sections 1.3 and 1.4) would be applied to the candidate GEINs. After that, by using PNP method (Supplementary Material Section 1.5) on the real GEINs, the essential elements would be kept so that we could extract core HPCNs in respect of KEGG pathways to do further analysis.

## Figures and Tables

**Table 1:** Information about the numbers of nodes of candidate GEINs and real GEINs by the proposed system identification method in the infection of *C. albicans* SC5314 and *C. albicans* WO-1 of two replicates.

Nodes	Candidates_SC5314	SC5314_R1	SC5314_R2	Candidates_WO-1	WO-1_R1	WO-1_R2
HP	23,217	15,817	9979	20,694	14,682	15,621
HR	3025	1960	1141	2488	1719	1743
HT	5649	855	357	4696	384	427
HM	607	385	370	412	331	342
HL	333	166	101	258	131	129
PP	3766	2414	2195	3736	3413	3411
PT	487	254	255	483	253	280
Total nodes	37,229	21,851	14,398	32,767	20,913	21,953

SC5314_R1 and SC5314_R2 represent replicate 1 and replicate 2 of SC5314, respectively. Similarly, WO-1_R1 and WO-1_R2 denote replicate 1 and replicate 2 of WO-1, respectively. HP denotes host protein (excluding host receptor and host TF); HR, HT, HM, and HL represent host receptor, host TF, host miRNA, and host lncRNA, respectively. PP means pathogen protein excluding pathogen TF and PT represents pathogen TF.

**Table 2:** Information about the identified number of edges of candidate interspecies GEINs and real interspecies GEINs by proposed system identification method in the infection of *C. albicans* SC5314 and *C. albicans* WO-1 of two replicates.

Edges	Candidates_SC5314	SC5314R1	SC5314R2	Candidates_WO-1	WO-1_R1	WO-1_R2
HT→HG	152,491	8036	5039	141,001	7963	7638
HT→HM	1347	154	21	901	62	42
HT→HL	271	162	98	200	129	124
HL→HG	37	1	0	35	0	0
HM→HG	170,671	2401	2408	118,017	2799	3744
HM→HL	130	4	3	78	2	5
HM→HM	45	6	1	21	0	4
HT→PG	21,328	374	242	20,177	586	489
HM→PG	23,730	394	328	16,639	543	771
HL→PG	1	0	0	1	0	0
PT→HM	48	9	1	29	0	1
PT→HG	8910	208	169	8726	220	248
PT→PG	86,491	7225	5112	85,211	5723	7167
HG—HG	6,449,171	30,892	24,665	5,521,448	119,623	127,687
HG—PG	1,615,845	10,248	7540	1,453,353	45,214	47,146
PG—PG	132,600	4830	3988	131,485	33,643	34,707

→: Transcriptional or post-transcriptional regulation. —: Protein–protein interaction. HG: Host genes. PG: Pathogen genes.

**Table 3:** The specific and common host cellular functions and functional abundance analysis of related pathways of the conserved host target-genes between 2 replicates in the infection of *C. albicans* SC5314 and WO-1 on the basis of GO terms by applying the DAVID analysis.

**SC5314**		
**Category**	**Term**	***p*-Value**
GOTERM_MF_DIRECT	GO:0016712~oxidoreductase activity (0.47%)	0.00298310389230338
GOTERM_MF_DIRECT	GO:0003676~nucleic acid binding (5.5%)	0.0129970927095585
UP_SEQ_FEATURE	Metal ion-binding (0.43%)	0.0176534099242106
GOTERM_BP_DIRECT	GO:0016338~cell adhesion (0.3%)	0.0181497359081671
KEGG_PATHWAY	hsa03030:DNA replication (0.39%)	0.034840174
**WO-1**		
**Category**	**Term**	***p*-Value**
GOTERM_MF_FAT	GO:0008135~nucleic acid binding (0.89%)	0.00700213339377146
UP_SEQ_FEATURE	Metal ion-binding (0.45%)	0.00288724333819777
KEGG_PATHWAY	hsa03030:DNA replication (0.45%)	0.015170596995848
SP_PIR_KEYWORDS	cell adhesion (2.64%)	0.0332759579717729
GOTERM_MF_FAT	GO:0016651~oxidoreductase activity (0.7%)	0.0337336128643766

The *C. albicans* SC5314 infection is characterized by the transformation of host cell shape, and this will result in the activities of GTPases because pathogen proteins adhere to host cell surface. However, while *C. albicans* SC5314 executes endocytosis function, cytoskeleton and cytoplasm change cell type in the host cells so that it will produce DNA replication and nucleic acid binding to activate gene expression. Additionally, the metal ion-binding ability plays a crucial role for human and pathogen because of its function in finding metallic nutrients. By this process, host cell also produces ROS-related molecule although the ion is toxic. Applying the ROS-related molecule also leads to eliminating *C. albicans* SC5314. Similarly, the *C. albicans* WO-1 infection is characterized by the transformation of host cell shape, and this will result in the activities of GTPases because pathogen proteins adhere to host cell surface. However, while *C. albicans* WO-1 executes endocytosis function, cytoskeleton and cytoplasm change cell type in the host cells so that it will produce DNA replication and nucleic acid binding to activate gene expression. Additionally, the metal ion-binding ability plays a crucial role for human and pathogen because of its function in finding metallic nutrients. By this process, host cell also produces ROS-related molecule although the ion is toxic. Applying the ROS-related molecule also leads to eliminating *C. albicans* WO-1.

**Table 4:** The specific and common pathogen functions and functional abundance analysis of related pathways of the conserved target- pathogen genes between 2 replicates in the infection of *C. albicans* SC5314 and WO-1 on the basis of GO terms by applying the Candida Genome Database (CGD) Gene Ontology (GO) Term Finder analysis.

**SC5314**		
**GOID**	**GO_Term**	***p*-Value**
5198	structural molecule activity (6.8%)	3.18 × 10^−19^
5515	protein binding (12.2%)	8.85 × 10^−15^
16740	transferase activity (15.6%)	4.38 × 10^−10^
98772	molecular function regulator (5.7%)	2.53 × 10^−8^
16787	hydrolase activity (16.3%)	5.32 × 10^−5^
**WO-1**		
**Category**	**GO_Term**	***p*-Value**
5515	protein binding (12.2%)	2.14 × 10^−17^
16787	hydrolase activity (17.7%)	1.13 × 10^−14^
16740	transferase activity (15.8%)	6.16 × 10^−13^
98772	molecular function regulator (5.7%)	7.02 × 10^−10^
5198	structural molecule activity (5.4%)	4.98 × 10^−6^

The *C. albicans* SC5314 pathogenic function is considered by morphological transformation including structural molecule activity, molecular functional regulation, hydrolase activity and transferase activity. It is noted that *C. albicans* is influenced by transferase activity so that it will promote hyphal growth. Moreover, hydrolase activity is thought to facilitate penetration into host cells. Similar gene ontology terms can be observed in *C. albicans* WO-1.

## Supplementary Materials

The following are available online at http://www.mdpi.com/2072-6651/11/2/119/s1. Figure S1: The constructing programs of candidate GEIN. (A) *C. albicans* SC5314- *C. albicans* SC5314 candidate intra-species PPIN; (B) host-*C. albicans* SC5314 candidate inter-species PPIN; (C) candidate GRN of host-TFs targeting *C. albicans* SC5314-genes; (D) candidate GRN of host-miRNAs targeting *C. albicans* SC5314-genes; (E) candidate GRN of *C. albicans* SC5314 TFs targeting host-genes; (F) candidate GRN of *C. albicans* SC5314 TFs targeting host- miRNAs; G) candidate GRN of *C. albicans* SC5314 TFs targeting *C. albicans* SC5314-genes, Figure S2: The constructing programs of candidate GEIN. (A) *C. albicans* WO-1- *C. albicans* WO-1 candidate intra-species PPIN; (B) host-*C. albicans* WO-1 candidate inter-species PPIN; (C) candidate GRN of host-TFs targeting *C. albicans* WO-1-genes; (D) candidate GRN of host-miRNAs targeting *C. albicans* WO-1-genes; (E) candidate GRN of *C. albicans* WO-1 TFs targeting host-genes; (F) candidate GRN of *C. albicans* WO-1 TFs targeting host- miRNAs; (G) candidate GRN of *C. albicans* WO-1 TFs targeting *C. albicans* WO-1-genes, Figure S3: The real GEINs of two replicates during the infection of *C. albicans* SC5314 and *C. albicans* WO-1 with OKF6/TERT-2 cells, respectively, Figure S4: Core HPCN of OKF6/TERT-2 cells during the infection of *C. albicans* SC5314, Figure S5: Core HPCN of OKF6/TERT-2 cells during the infection of *C. albicans* WO-1.
