# Supplementary material for: Investigating Common Pathogenic Mechanisms between Homo sapiens and Different Strains of Candida albicans for Drug Design: Systems Biology Approach via Two-Sided NGS Data Identification
Source: Toxins (Basel). 2019 Feb 15;11(2):119. doi: 10.3390/toxins11020119 (PMC6409619; doi:10.3390/toxins11020119)
Supplement: Supplementary file 1 [file toxins-11-00119-s001.pdf]

toxins

Supplementary Materials: Investigating Common Pathogenic Mechanisms between *Homo sapiens* and Different Strains of *Candida albicans* for Drug Design: Systems Biology Approach via Two-sided NGS Data Identification

Shan-Ju Yeh, Chun-Chieh Yeh, Chung-Yu Lan and Bor-Sen Chen

1.1. The Construction of Candidate Genetic and Epigenetic Interspecies Networks (GEINs) for C. albicans SC5314 and WO-1

The construction procedures of candidate GEINs for two strains of *C. albicans* SC5314 and WO-1 are shown in Figure S1. In the intra-species candidate PPIN, we obtained 132,600 PPI pairs of *C. albicans* SC5314 and 6,449,171 PPI pairs of human; in the interspecies candidate PPIN, we obtained 1,615,845 PPI pairs between human and *C. albicans* SC5314. In the intra-species candidate GRN, we obtained 86,491 TF-gene pairs of *C. albicans* SC5314, 152,491 TF-gene pairs of human cells, 170,671 miRNA-gene pairs of human cells, and 37 lncRNA-gene pairs of human cells, 45 miRNA-miRNA pairs of human cells, 130 miRNA-lncRNA pairs of human cells, 271 TF-lncRNA pairs of human cells, 1,347 TF-miRNA pairs of human cells; in the inters-species candidate GRN, we obtained 8,910 pairs between *C. albicans* SC5314 TF and human gene, 48 pairs between *C. albicans* SC5314 TF and human miRNA, 21,328 pairs between human TF and *C. albicans* SC5314 gene, and 23,730 pairs between human miRNA and *C. albicans* SC5314 gene, 1 pair between human lncRNA and *C. albicans* SC5314 gene.

Likewise, in the intra-species candidate PPIN, we obtained 131,485 PPI pairs of *C. albicans* WO-1 and 5,521,448 PPI pairs of human cells; in the interspecies candidate PPIN, we obtained 1,453,353 PPI pairs between human and *C. albicans* WO-1 PPI pairs. In the intra-species candidate GRN, we obtained 85,211 TF-gene pairs of *C. albicans* WO-1, 141,001 TF-gene pairs of human cells, 118,017 miRNA-gene pairs of human cells, and 35 lncRNA-gene pairs of human cells, 21 miRNA-miRNA pairs of human cells, 78 miRNA-lncRNA pairs of human cells, 200 TF-lncRNA pairs of human cells, 901 TF-miRNA pairs of human cells. ; in the interspecies candidate GRN, we obtained 8,726 pairs between C. albicans WO-1 TF and human gene, 29 between *C. albicans* WO-1 TF and human miRNA, 20,177 pairs between human TF and *C. albicans* WO-1 gene, and 16,639 pairs between human miRNA and *C. albicans* WO-1 gene,1 pair between human lncRNA and C. albicans WO-1 gene.

In conclusion, we built two candidate GEINs composed of putative interactions and regulations pairs for two strains of *C. albicans* SC5314 and WO-1, respectively. After that, we discovered the real GEINs by pruning the false positives via the system order detection scheme and the system identification approach with the genome-wide NGS data of OKF6/TERT-2 cells, *C. albicans* SC5314 and WO-1 which would be discussed in the following section.

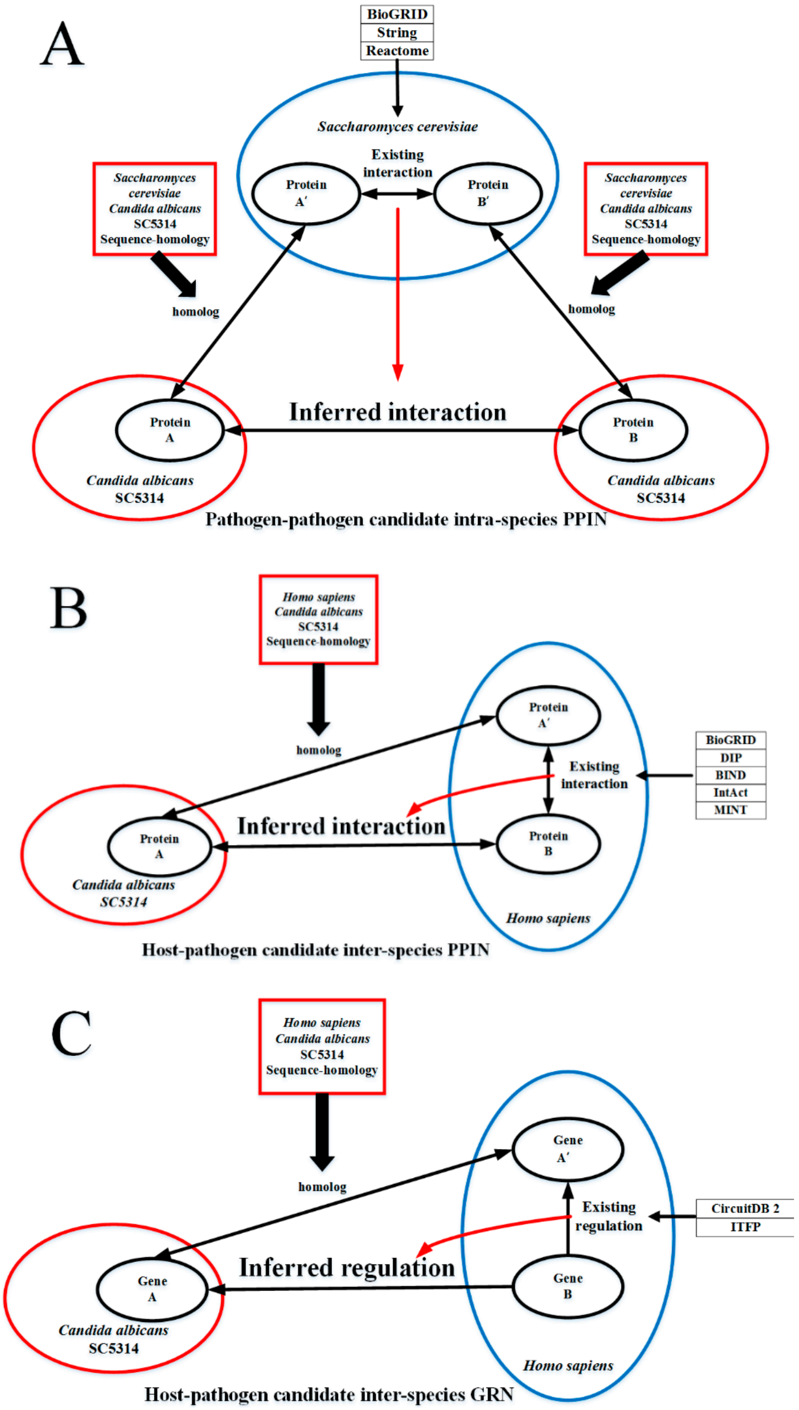
A
BioGRID
String
Reactome
Saccharomyces cerevisiae
Existing interaction
Protein A′
Protein B′
Saccharomyces cerevisiae Candida albicans SC5314 Sequence-homology
Saccharomyces cerevisiae Candida albicans SC5314 Sequence-homology
homolog
homolog
Inferred interaction
Protein A
Protein B
Candida albicans SC5314
Candida albicans SC5314
Pathogen-pathogen candidate intra-species PPIN
B
Homo sapiens Candida albicans SC5314 Sequence-homology
homolog
Protein A′
Existing interaction
BioGRID
DIP
BIND
IntAct
MINT
Inferred interaction
Protein A
Protein B
Candida albicans SC5314
Homo sapiens
Host-pathogen candidate inter-species PPIN
C
Homo sapiens Candida albicans SC5314 Sequence-homology
homolog
Gene A′
Existing regulation
CircuitDB 2
ITFP
Inferred regulation
Gene A
Gene B
Candida albicans SC5314
Homo sapiens
Host-pathogen candidate inter-species GRN

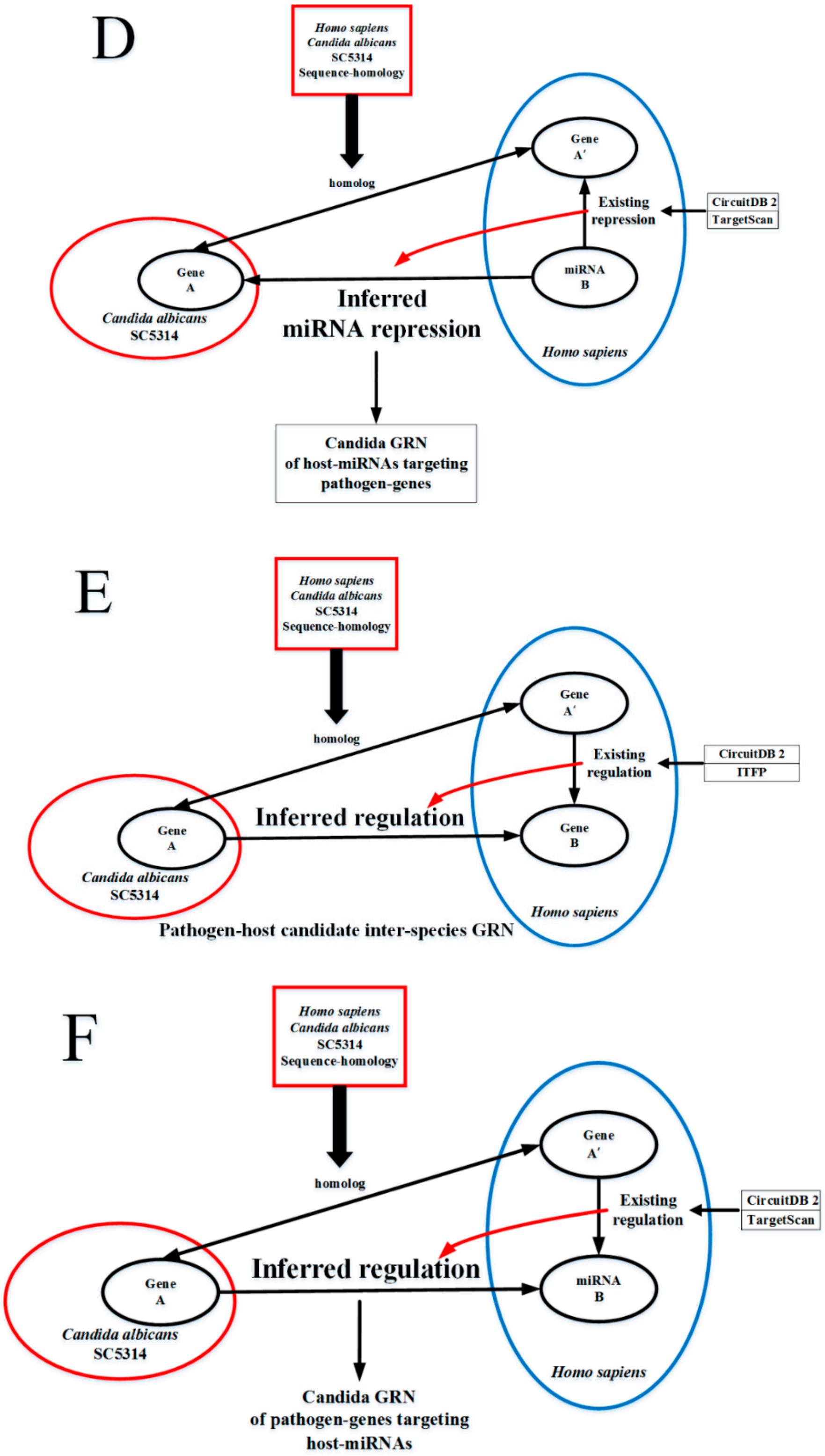
D
Homo sapiens
Candida albicans
SC5314
Sequence-homology
homolog
Gene
A′
Existing
repression
CircuitDB 2
TargetScan
Gene
A
miRNA
B
Inferred
miRNA repression
Candida albicans
SC5314
Homo sapiens
Candida GRN
of host-miRNAs targeting
pathogen-genes
E
Homo sapiens
Candida albicans
SC5314
Sequence-homology
homolog
Gene
A′
Existing
regulation
CircuitDB 2
ITFP
Inferred regulation
Gene
A
Gene
B
Candida albicans
SC5314
Homo sapiens
Pathogen-host candidate inter-species GRN
F
Homo sapiens
Candida albicans
SC5314
Sequence-homology
homolog
Gene
A′
Existing
regulation
CircuitDB 2
TargetScan
Inferred regulation
Gene
A
miRNA
B
Candida albicans
SC5314
Homo sapiens
Candida GRN
of pathogen-genes targeting
host-miRNAs

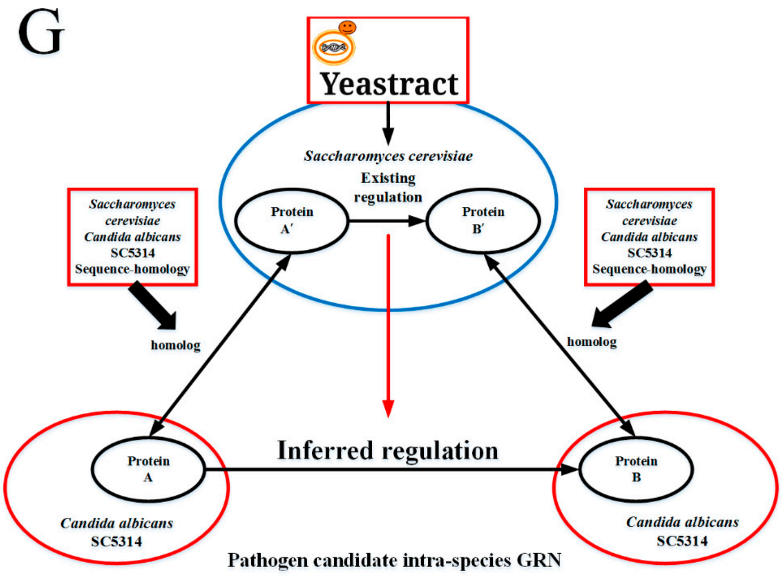

Figure 1. The constructing programs of candidate GEIN. (**A**) *C. albicans* SC5314- *C. albicans* SC5314 candidate intra-species PPIN; (**B**) host-*C. albicans* SC5314 candidate inter-species PPIN; (**C**) candidate GRN of host-TFs targeting *C. albicans* SC5314-genes; (**D**) candidate GRN of host-miRNAs targeting *C. albicans* SC5314-genes; (**E**) candidate GRN of *C. albicans* SC5314 TFs targeting host-genes; (**F**) candidate GRN of *C. albicans* SC5314 TFs targeting host- miRNAs; (**G**) candidate GRN of *C. albicans* SC5314 TFs targeting *C. albicans* SC5314-genes. The sequence-homolog pairs between *S.cerevisiae* and *C. albicans* SC5314, and *C. albicans* SC5314 with homo sapiens were acquired from the reference and met with the standard of sequence-homolog (*E*-value < 10^{-5}, Identificatinon > 30%, Overlap > 80%). With the assistance of existing *S.cerevisiae* intra-species PPIN and host intra-species PPIN, we can infer potential *C. albicans* SC5314 intra-species PPIN and human-*C. albicans* SC5314 interspecies PPIN as shown in (**A**,**B**), respectively; similarly, we can infer potential human to *C. albicans* SC5314 interspecies GRN by sequence-homolog, CircuitDB2, ITFP and TargetScan as shown in (**C**,**D**), respectively; similarly we can infer potential *C. albicans* SC5314 to human interspecies GRN by sequence-homolog, CircuitDB2, ITFP and TargetScan as shown in (**E**,**F**), respectively; finally, we can infer potential *C. albicans* SC5314 intra-species GRN by sequence-homolog, Yeastract.

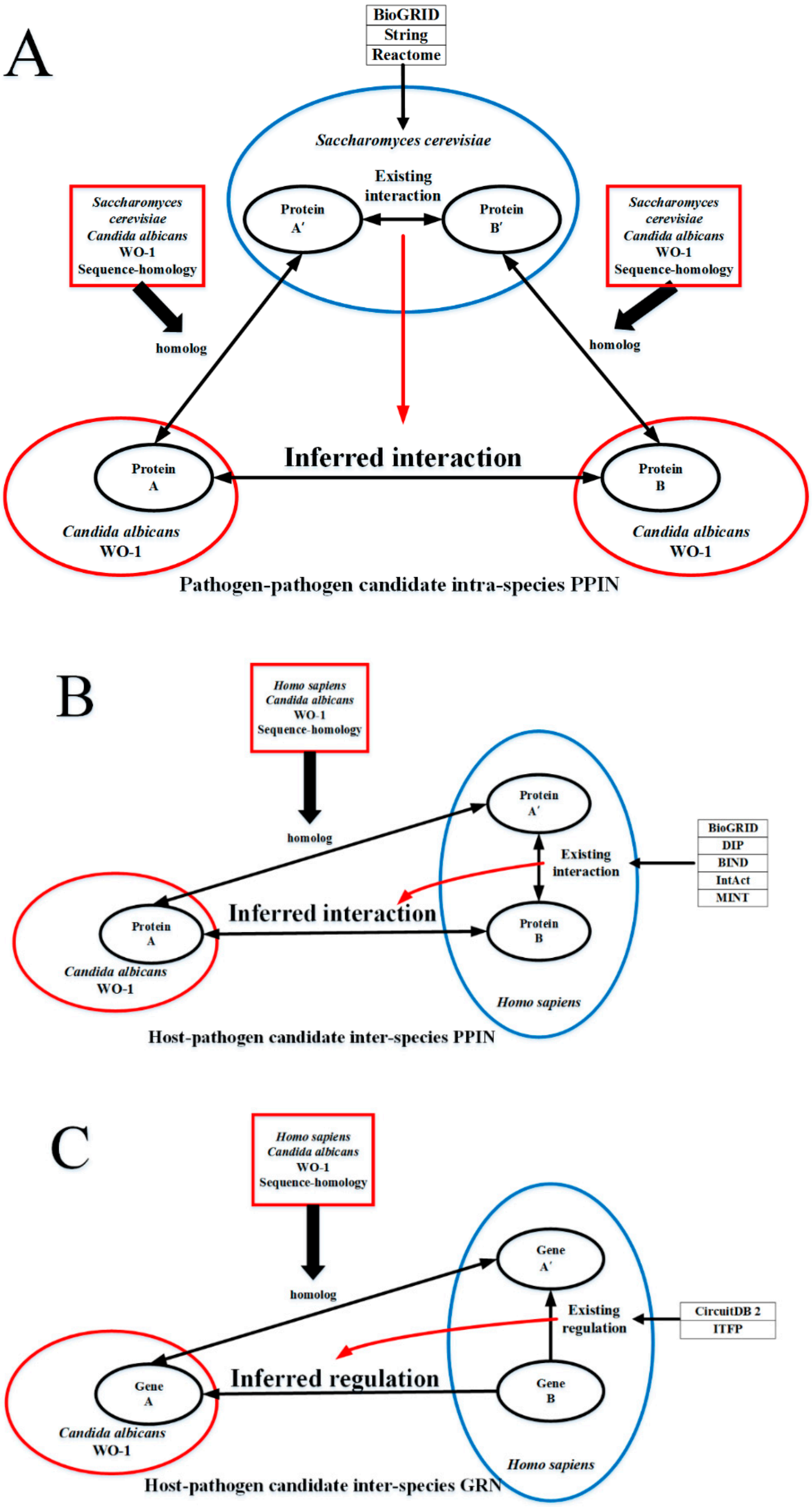
A
BioGRID
String
Reactome
Saccharomyces cerevisiae
Existing interaction
Protein A′
Protein B′
Saccharomyces cerevisiae Candida albicans WO-1 Sequence-homology
Saccharomyces cerevisiae Candida albicans WO-1 Sequence-homology
homolog
homolog
Inferred interaction
Protein A
Protein B
Candida albicans WO-1
Candida albicans WO-1
Pathogen-pathogen candidate intra-species PPIN
B
Homo sapiens Candida albicans WO-1 Sequence-homology
homolog
Protein A′
Existing interaction
Protein B
BioGRID
DIP
BIND
IntAct
MINT
Inferred interaction
Protein A
Candida albicans WO-1
Homo sapiens
Host-pathogen candidate inter-species PPIN
C
Homo sapiens Candida albicans WO-1 Sequence-homology
homolog
Gene A′
Existing regulation
CircuitDB 2
ITFP
Inferred regulation
Gene A
Gene B
Candida albicans WO-1
Homo sapiens
Host-pathogen candidate inter-species GRN

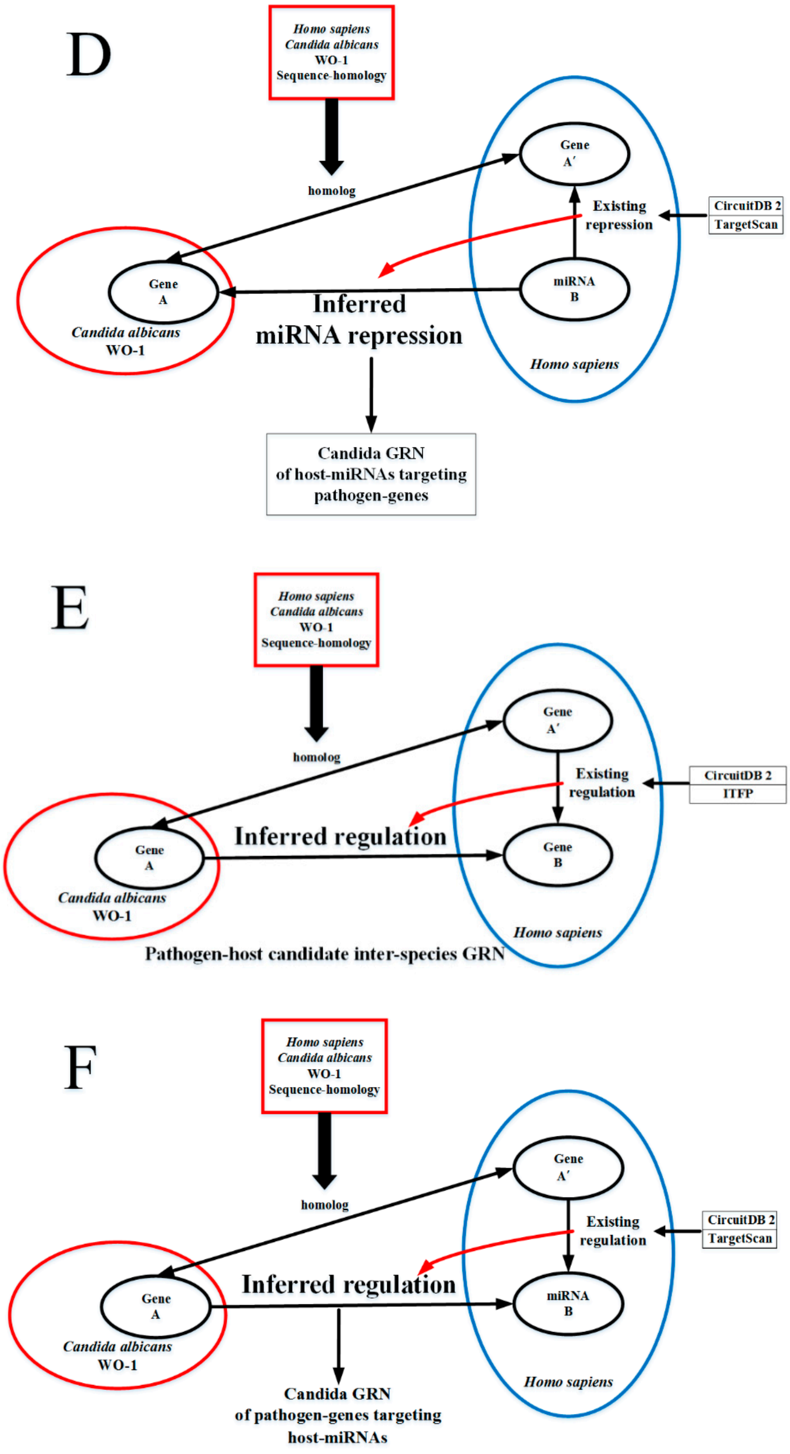
D
Homo sapiens
Candida albicans
WO-1
Sequence-homology
homolog
Gene
A′
Existing
repression
CircuitDB 2
TargetScan
Gene
A
miRNA
B
Inferred
miRNA repression
Candida albicans
WO-1
Homo sapiens
Candida GRN
of host-miRNAs targeting
pathogen-genes
E
Homo sapiens
Candida albicans
WO-1
Sequence-homology
homolog
Gene
A′
Existing
regulation
CircuitDB 2
ITFP
Inferred regulation
Gene
A
Gene
B
Candida albicans
WO-1
Homo sapiens
Pathogen-host candidate inter-species GRN
F
Homo sapiens
Candida albicans
WO-1
Sequence-homology
homolog
Gene
A′
Existing
regulation
CircuitDB 2
TargetScan
Inferred regulation
Gene
A
miRNA
B
Candida albicans
WO-1
Homo sapiens
Candida GRN
of pathogen-genes targeting
host-miRNAs

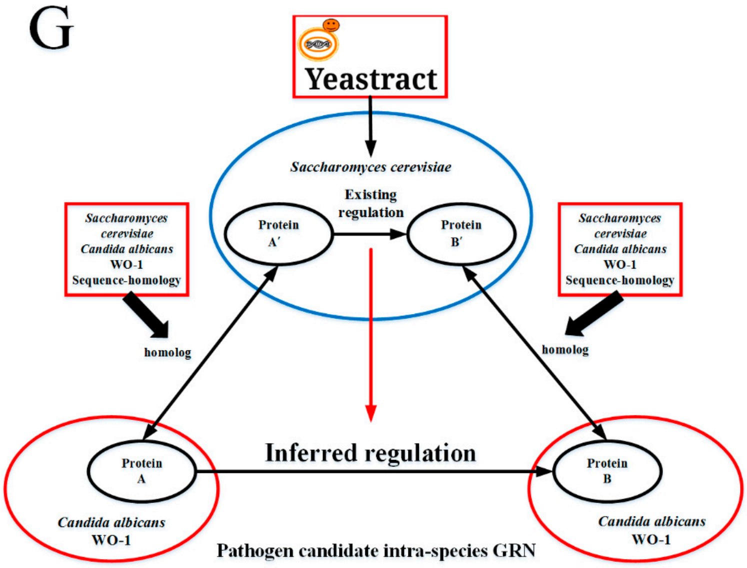

Figure 2. The constructing programs of candidate GEIN. (**A**) *C. albicans* WO-1- *C. albicans* WO-1 candidate intra-species PPIN; (**B**) host-*C. albicans* WO-1 candidate inter-species PPIN; (**C**) candidate GRN of host-TFs targeting *C. albicans* WO-1-genes; (**D**) candidate GRN of host-miRNAs targeting *C. albicans* WO-1-genes; (**E**) candidate GRN of *C. albicans* WO-1 TFs targeting host-genes; (**F**) candidate GRN of *C. albicans* WO-1 TFs targeting host- miRNAs; G) candidate GRN of *C. albicans* WO-1 TFs targeting *C. albicans* WO-1-genes. The sequence-homolog pairs between *S.cerevisiae* and *C. albicans* WO-1, and *C. albicans* WO-1 with *homo sapiens* were acquired from the reference and met with the standard of sequence-homolog (*E*-value < 10^{-5}, Identificatinon>30%, Overlap>80%).With the assistance of existing *S.cerevisiae* intra-species PPIN and host intra-species PPIN, we can infer potential *C. albicans* WO-1 intra-species PPIN and human- *C. albicans* WO-1 interspecies PPIN as shown in (**A,B**), respectively; similarly, we can infer potential human to *C. albicans* WO-1 interspecies GRN by sequence-homolog, CircuitDB2, ITFP and TargetScan as shown in (**C,D**), respectively; similarly we can infer potential *C. albicans* WO-1 to human interspecies GRN by sequence-homolog, CircuitDB2, ITFP and TargetScan as shown in (**E,F**), respectively; finally, we can infer potential *C. albicans* WO-1 intra-species GRN by sequence-homolog, Yeastract.

1.2. Dynamic Models of Candidate GEINs for OKF6/TERT-2 Cells and C. albicans during the Infection

The candidate GEIN is composed of the experimental results and computational predictions from numerous databases, experimental datasets and literatures. Therefore, the candidate GEIN contains a number of false positive regulations and interactions. To reduce the effect of these false-positive information, we built the dynamic models to characterize the molecular-mechanisms of GEINs and to prune the false-positives for OKF6/TERT-2 cells infected by *C. albicans*. We then extract the core host-pathogen cross-talk network (HPCN) by the principal network projection (PNP) scheme to characterize the principal pathogenic mechanisms in GEINs during *C. albicans* infection.

The PPIs of human-protein i in the candidate PPIN can be described as the following stochastic dynamic interactive equation,

$$p_i^H(\mathrm{t}+1) = p_i^H(\mathrm{t}) + \sum_{n=1}^{N_i} a_{in}^H p_i^H(\mathrm{t}) p_n^H(\mathrm{t}) + \sum_{j=1}^{J_i} b_{ij}^H p_i^H(\mathrm{t}) p_j^P(\mathrm{t}) + \alpha_i^H g_i^H(\mathrm{t})$$
$$- \beta_i^H p_i^H(\mathrm{t}) + \chi_i^H + \omega_i^H(\mathrm{t}),\ \text{for}\ i = 1, 2, \ldots, I,\ \alpha_i^H \geq 0\ \text{and}\ -\beta_i^H \leq 0 \quad (1)$$

where p_i^H(t), p_n^H(t), g_i^H(t) and p_j^P(t) indicate the expression levels of the *i*th host protein, the *n*th host protein, the *i*th host gene and the *j*th pathogen protein at time *t*, respectively; a_{in}^H and b_{ij}^H represent the interactive ability between the *i*th host protein and *n*th host protein and between the *i*th host protein and *j*th pathogen protein, respectively; α_i^H, $-\beta_i^H$ and χ_i^H signify the translation rate from the corresponding mRNA, the degradation rate and the expression basal level of the *i*th host protein, respectively; N_i and J_i denote the number of host proteins and pathogen proteins that interact with the *i*th host protein, respectively; ω_i^H(t) indicates the stochastic noise of the expression level of the *i*th host protein at time *t*. The biological meaning of the equation (1) is that the expression level of the *i*th host protein can be affected by various molecular mechanisms including the host intra-species PPIs by $\sum_{n=1}^{N_i} a_{in}^H p_i^H(t)\, p_n^H(t)$,the inter-species PPIs by $\sum_{j=1}^{J_i} b_{ij}^H p_i^H(t) p_j^P(t)$, the protein translation by $\alpha_i^H g_i^H$(t), the protein degradation by $-\beta_i^H p_j^P$(t), the expression basal level by χ_i^H and the stochastic noise by ω_i^H(t). Furthermore, the protein degradation rate β_i^H should be limited to be non-negative and the translation rate α_i^H should be limited to be non-negative in real PPIs.

The PPIs of pathogen-protein *j* in the candidate PPIN can be described as the following stochastic dynamic interactive equation,

$$p_j^P(\mathrm{t}+1) = p_j^P(\mathrm{t}) + \sum_{o=1}^{O_j} c_{jo}^P p_j^P(\mathrm{t}) p_o^P(\mathrm{t}) + \sum_{i=1}^{I_j} d_{ji}^P p_j^P(\mathrm{t}) p_i^H(\mathrm{t}) + \delta_j^P g_j^P(\mathrm{t}) - \varepsilon_j^P p_j^P(\mathrm{t}) + \chi_j^P + \omega_j^P(\mathrm{t}), \text{ for } j = 1,2,...,J,\ \delta_j^P \ge 0 \text{ and } -\varepsilon_j^P \le 0 \tag{2}$$

where p_j^P(t), p_o^P(t), g_j^P(t) and p_i^H(t) indicate the expression levels of the *j*th pathogen protein, the *o*th pathogen protein, the *j*th pathogen gene and the *i*th host protein at time *t*, respectively; c_{jo}^P and d_{ji}^P represent the interactive ability between the *j*th pathogen protein and *o*th pathogen protein and between the *j*th pathogen protein and *i*th host protein, respectively; δ_j^P, $-\varepsilon_j^P$ and χ_j^P signify the translation rate, the degradation rate and the expression basal level of the *j*th pathogen protein, respectively; O_j and I_j denote the number of pathogen proteins and host proteins that interact with the *j*th pathogen protein; ω_j^P (t) indicates the stochastic noise of the expression level of the *j*th pathogen protein at time *t*. The biological meaning of the equation (2) is that the expression level of the *j*th pathogen protein can be affected by various molecular mechanisms including the pathogen intra-species PPIs by $\sum_{o=1}^{O_j} c_{jo}^P p_j^P(t) p_o^P(t)$,the inter-species PPIs by $\sum_{i=1}^{I_j} d_{ji}^P p_j^P(t) p_i^H(t)$, the protein translation by $\delta_j^P g_j^P$(t), the protein degradation by $-\varepsilon_j^P p_i^H$(t), the expression basal level by χ_j^P and the stochastic noise by ω_i^H(t). Furthermore, similar to the host protein dynamic model, the protein degradation rate ε_j^P should be limited to be non-negative and the translation rate δ_j^P should be limited to be non-negative in real PPIs.

The transcriptional regulations of host-gene *k* in the candidate GRN can be described as the following stochastic dynamic interactive equation,

$$g_k^H(\mathrm{t}+1) = g_k^H(\mathrm{t}) + \sum_{i=1}^{I_k} e_{ki}^H p_i^H(\mathrm{t}) - \sum_{l=1}^{L_k} f_{kl}^H g_k^H(\mathrm{t}) m_l^H(\mathrm{t}) + \sum_{m=1}^{M_k} h_{km}^H l_m^H(\mathrm{t}) + \sum_{j=1}^{J_k} n_{kj}^H p_j^P(\mathrm{t}) - \phi_k^{\mathrm{H}} g_k^H(\mathrm{t}) + \varphi_k^H + \varpi_k^H(\mathrm{t}), \text{ for } k = 1,2,...,K,\ -f_{kl}^H \le 0 \text{ and } -\phi_k^{\mathrm{H}} \le 0 \tag{3}$$

where g_k^H(t), p_i^H(t), m_l^H(t) , l_m^H(t) and p_j^P(t) indicate the expression levels of the *k*th host gene, the *i*th host TF, the *l*th host microRNA, the *m*th host lncRNA and the *j*th pathogen TF at time *t*, respectively; e_{ki}^H, $-f_{kl}^H$, h_{km}^H and n_{kj}^H represent the the regulatory ability of the *i*th host TF, the *l*th host miRNA, the *m*th host lncRNA and the *j*th pathogen TF on the *k*th host gene, respectively; $-\phi_k^H$ and φ_k^H signify the degradation rate and the expression basal level of the *k*th host gene, respectively; In fact, the basal level in equation (3) indicates

unknown regulations other than those mentioned above, for example, DNA methylation and regulatory multiple epigenetic activities. I_k, L_k, M_k, J_k denote the number of host TF, host microRNA, host lncRNA and pathogen TF, respectively; which regulate the expression level of the *k*th host gene; ϖ_k^H(t) indicates the stochastic noise of the gene expression level of the *k*th host gene at time *t*. The biological meaning of the equation (3) is that the expression level of the *k*th host gene can be regulated by various molecular mechanisms including the host TF regulations by $\sum_{i=1}^{I_k} e_{ki}^H p_i^H$(t), the host microRNA repressions by $-\sum_{l=1}^{L_k} f_{kl}^H g_k^H(t)m_l^H$(t), the host lncRNA regulations by $\sum_{m=1}^{M_k} h_{km}^H l_m^H$(t), the pathogen TF regulations by $\sum_{j=1}^{J_k} n_{kj}^H p_j^P$(t), the mRNA degradation by $-\phi_k^H$, the expression basal level by φ_k^H and the stochastic noise by ϖ_k^H (t).In addition, similar to protein model, the host gene degradation rate $-\phi_k^H$ should be limited to be non-positive and the host miRNA regulatory ability $-f_{kl}^H$ should be limited to be non-positive.

The transcriptional regulations of host-miRNA *l* in the candidate GRN can be described as the following stochastic dynamic regulatory equation,

$$m_l^H(\mathrm{t}+1) = m_l^H(\mathrm{t}) + \sum_{i=1}^{I_l} o_{li}^H p_i^H(\mathrm{t}) - \sum_{r=1}^{R_l} q_{lr}^H m_l^H(\mathrm{t}) m_r^H(\mathrm{t}) + \sum_{j=1}^{J_l} r_{lj}^P p_j^P(\mathrm{t})$$
$$-\gamma_l^{\mathrm{H}} m_l^H(\mathrm{t}) + \kappa_l^H + \eta_l^H(\mathrm{t}), \text{ for } l = 1,2,...,L, -q_{lr}^H \le 0 \text{ and } -\gamma_l^{\mathrm{H}} \le 0 \quad (4)$$

where m_l^H(t), p_i^H(t), m_r^H(t) and p_j^P(t) indicate the expression levels of the *l*th host miRNA, the *i*th host TF, the *r*th host microRNA and the *j*th pathogen TF at time *t*, respectively; o_{li}^H, $-q_{lr}^H$ and r_{lj}^P represent the the regulatory ability of the *i*th host TF, the *r*th host miRNA, and the *j*th pathogen TF on the *l*th host miRNA, respectively; $-\gamma_l^H$ and κ_l^H signify the miRNA degradation rate and the expression basal level of the *l*th host miRNA, respectively; I_l, R_l and J_l denote the number of host TF, host miRNA and pathogen TF, respectively; which regulate the expression level of the *l*th host miRNA; η_l^H(t) indicates the stochastic noise of the *l*th host miRNA at time *t*. The biological meaning of the equation (4) is that the expression level of the *k*th host gene can be regulated by various molecular mechanisms including the host TF regulations by $\sum_{i=1}^{I_l} o_{li}^H p_i^H$(t), the host microRNA repressions by $-\sum_{r=1}^{R_l} q_{lr}^H m_l^H(t)m_r^H$(t), and the pathogen TF regulations by $\sum_{j=1}^{J_l} r_{lj}^P p_j^P$(t) ,the mRNA degradation by $-\gamma_l^H m_l^H$(t), the expression basal level by κ_l^H and the stochastic noise by η_l^H(t) . In addition, similar to host gene model, the host miRNA degradation rate $-\gamma_l^H$ should be limited to be non-positive and the miRNA regulatory ability $-q_{lr}^H$ should be limited to be non-positive.

The transcriptional regulations of host-lncRNA *m* in the candidate GRN can be described as the following stochastic dynamic regulatory equation,

$$l_m^H(\mathrm{t}+1) = l_m^H(\mathrm{t}) + \sum_{i=1}^{I_m} s_{mi}^H p_i^H(\mathrm{t}) - \sum_{l=1}^{L_m} t_{ml}^H l_m^H(\mathrm{t}) m_l^H(\mathrm{t}) - \mu_m^H l_m^H(\mathrm{t}) + \pi_m^H + \vartheta_m^H(\mathrm{t})$$
$$\text{for } m = 1,2,...,M, -t_{ml}^H \le 0 \text{ and } -\mu_m^H \le 0 \quad (5)$$

where l_m^H(t), p_i^H(t) and m_l^H(t) indicate the expression levels of the *m*th host lncRNA, the *i*th host TF and the *l*th host microRNA at time *t*, respectively; s_{mi}^H and $-t_{ml}^H$ represent the regulatory ability of the *i*th host TF and the *l*th host miRNA on the *m*th host lncRNA, respectively; $-\mu_m^H$ and π_m^H signify the degradation rate and the expression basal level of the *m*th host lncRNA, respectively; I_m and L_m denote the number of host TF and host microRNA, respectively; which regulate the expression level of the *m*th host lncRNA; ϑ_m^H(t) indicates the stochastic noise of the *m*th host gene at time *t*. The biological meaning of the equation (5) is that the expression level of the *m*th host lncRNA can be regulated by various molecular mechanisms including the host TF regulations by $\sum_{i=1}^{I_m} s_{mi}^H p_i^H$ (t), the host microRNA repressions by $-\sum_{l=1}^{L_k} t_{ml}^H l_m^H$ (t)m_l^H (t),the lncRNA degradation by $-\mu_m^H l_m^H$(t), the expression basal level by π_m^H and the stochastic noise by ϑ_m^H(t).In addition,

similar to host gene model, the host lncRNA degradation rate $-\mu_m^H$ should be limited to be non-positive and the host miRNA regulatory ability $-t_{ml}^H$ should be limited to be non-positive.

The transcriptional regulations of pathogen-gene n in the candidate GRN can be described as the following stochastic dynamic regulatory equation,

$$g_n^P(t+1) = g_n^P(t) + \sum_{i=1}^{I_n} u_{ni}^P p_i^H(t) - \sum_{l=1}^{L_n} v_{nl}^P g_n^P(t) m_l^H(t) + \sum_{m=1}^{M_n} w_{nm}^P l_m^H(t) + \sum_{j=1}^{J_n} x_{nj}^P p_j^P(t)$$
$$- \phi_n^P g_n^P(t) + \varphi_n^P + \omega_n^P(t), \text{ for } n = 1, 2, \ldots, N, -v_{nl}^P \le 0 \text{ and } -\phi_n^P \le 0 \quad (6)$$

where $g_n^P(t)$, $p_i^H(t)$, $m_l^H(t)$, $l_m^H(t)$ and $p_j^P(t)$ indicate the expression levels of the nth pathogen gene, the ith host TF, the lth host microRNA, the mth host lncRNA and the jth pathogen TF at time t, respectively; u_{ni}^P, $-v_{nl}^P$, w_{nm}^P and x_{nj}^P represent the the regulatory ability of the ith host TF, the lth host miRNA, the mth host lncRNA and the jth pathogen TF on the nth pathogen gene, respectively; $-\phi_n^P$ and φ_n^P signify the degradation rate and the expression basal level of the mth pathogen gene, respectively; I_n, L_n, M_n, J_n denote the number of host TF, host microRNA, host lncRNA and pathogen TF, respectively; which regulate the expression level of mth pathogen gene; $\omega_n^P(t)$ indicates the stochastic noise of the gene expression level of the nth pathogen gene at time t. The biological meaning of the equation (6) is that the expression level of the kth pathogen gene can be regulated by various molecular mechanisms including the host TF regulations by $\sum_{i=1}^{I_n} u_{ni}^P p_i^H(t)$, the host microRNA repressions by $-\sum_{l=1}^{L_k} v_{nl}^P g_n^P(t) m_l^H(t)$, the host lncRNA regulations by $\sum_{n=1}^{M_n} w_{nm}^P l_m^H(t)$, and the pathogen TF regulations by $\sum_{j=1}^{J_n} x_{nj}^P p_j^P(t)$,the mRNA degradation by $-\phi_n^P g_n^P(t)$, the expression basal level by φ_n^P and the stochastic noise by $\omega_n^P(t)$.In addition, similar to host gene regulatory model, the mRNA degradation rate $-\phi_n^P$ should be limited to be non-positive and the host miRNA regulatory ability $-v_{nl}^P$ should be limited to be non-positive.

Remark 1

The above dynamic models of interspecies GEIN mentioned are also employed for the infection of *Candida albicans* WO-1. Since *Candida albicans* WO-1 and *Candida albicans* SC5314 have the same candidate GEIN, the dynamic models between human and *C. albicans* WO-1 are along the interspecies GEIN of the infection of *C. albicans* SC5314. Additionally, without the regulation about lncRNAs-to-miRNA, lncRNAs-to-lncRNA, pathogen TFs-to-lncRNAs found in the candidate GEIN by big data mining, there are no corresponding regulatory terms in equations (4) and (5) in the infection of different strains of *C. albicans*.

1.3. Parameter Estimation of the Dynamic Models of Candidate GEIN by System Identification Approach

After constructing the dynamic model Equations (1)–(6) of the candidate GEINs, we should recognize the interactive parameters of PPIN in (1) and (2), and the regulatory parameters of GRN in (3)–(6) by employing the system identification approach via two-sided microarray data to obtain the real GEINs in the infection progress by pruning the false positive in the candidate GEINs. Accordingly, we rewrite the host PPIN dynamic equation as the linear regression form below [1–3],

$$p_i^H(\mathrm{t}+1) = [p_i^H p_1^H(\mathrm{t}) \cdots p_i^H p_{N_i}^H(\mathrm{t}) \; p_i^H p_1^H(\mathrm{t}) \cdots p_i^H p_{J_i}^H(\mathrm{t}) \; g_i^H(\mathrm{t}) \; p_i^H(\mathrm{t}) \; 1] \begin{bmatrix} a_{i1}^H \\ \vdots \\ a_{iN_i}^H \\ b_{i1}^H \\ \vdots \\ b_{iJ_i}^H \\ \alpha_i^H \\ 1-\beta_i^H \\ \chi_i^H \end{bmatrix} + \omega_i^H(\mathrm{t})$$
$$\square \; \psi_i^{HP}(\mathrm{t})\theta_i^{HP} + \omega_i^H(\mathrm{t}), \text{ for } i = 1,2,\ldots,I \quad (7)$$

where $\psi_i^{HP}(\mathrm{t})$ represents the regression vector that can be obtained from the microarray expression data and θ_i^{HP} is the unknown interaction parameter vector to be estimated for the *i*th host protein in host PPIN.

The expression Equation (7) of the *i*th host protein can be augmented for Y_i time points as the following form,

$$\begin{bmatrix} p_i^H(t_2) \\ p_i^H(t_3) \\ \vdots \\ p_i^H(t_{Y_i}+1) \end{bmatrix} = \begin{bmatrix} \psi_i^{HP}(t_1) \\ \psi_i^{HP}(t_2) \\ \vdots \\ \psi_i^{HP}(t_{Y_i}) \end{bmatrix} \theta_i^{HP} + \begin{bmatrix} \omega_i^H(t_1) \\ \omega_i^H(t_2) \\ \vdots \\ \omega_i^H(t_{Y_i}) \end{bmatrix}, \text{ for } i = 1,2,\ldots,I, \quad (8)$$

which could be simply represented by,

$$P_i^H = \Phi_i^{HP}\theta_i^{HP} + \Omega_i^{HP}, \text{ for } i = 1,2,\ldots,I \quad (9)$$

where $P_i^H = \begin{bmatrix} p_i^H(t_2) \\ p_i^H(t_3) \\ \vdots \\ p_i^H(t_{Y_i}+1) \end{bmatrix}$, $\Phi_i^{HP} = \begin{bmatrix} \psi_i^{HP}(t_1) \\ \psi_i^{HP}(t_2) \\ \vdots \\ \psi_i^{HP}(t_{Y_i}) \end{bmatrix}$, $\Omega_i^{HP} = \begin{bmatrix} \omega_i^H(t_1) \\ \omega_i^H(t_2) \\ \vdots \\ \omega_i^H(t_{Y_i}) \end{bmatrix}$

Thence, the interaction parameters in the vector θ_i^{HP} can be estimated by employing the following constrained least-squares estimation problem,

$$\min_{\theta_i^{HP}} \frac{1}{2}\left\| \Phi_i^{HP}\theta_i^{HP} - P_i^H \right\|_2^2$$
$$\text{subject to} \begin{bmatrix} 0 & \cdots & 0 & 0 & \cdots & 0 & -1 & 0 & 0 \\ 0 & \cdots & 0 & 0 & \cdots & 0 & 0 & 1 & 0 \end{bmatrix} \theta_i^{HP} \le \begin{bmatrix} 0 \\ 1 \end{bmatrix} \tag{10}$$

We can acquire the interaction parameters in host PPIN equation (1) by resolving the parameter estimation problem in (10) with the help of the function *lsqlin* in MATLAB optimization toolbox and simultaneously ensure the host protein translation rate α_i^H to be a non-negative value and the host protein degradation rate -β_i^H to be a non-positive value; that is to say $\alpha_i^H \ge 0$ and -$\beta_i^H \le 0$.

Similarly, we rewrite the pathogen PPIN dynamic interactive equation as the linear regression form below,

$$p_j^P(\mathrm{t}+1) = [p_j^P p_1^P(\mathrm{t}) \ \cdots \ p_j^H p_{O_j}^H(\mathrm{t}) \ \ p_j^P p_1^H(\mathrm{t})$$
$$\cdots \ p_j^H p_{Ij}^H(\mathrm{t}) \ \ \delta_j^P \ \ p_j^P(\mathrm{t}) \ \ 1] \begin{bmatrix} c_{j1}^H \\ \vdots \\ c_{jO_j}^H \\ d_{j1}^H \\ \vdots \\ d_{jI_j}^H \\ \delta_j^P \\ 1-\varepsilon_j^P \\ \chi_j^P \end{bmatrix} + \omega_j^P(\mathrm{t}) \tag{11}$$
$$\triangleq \psi_j^{PP}(\mathrm{t})\theta_j^{PP} + \omega_j^P(\mathrm{t}), \ \text{for } j = 1, 2, \ldots, J$$

where ψ_j^{PP}(t) represents the regression vector that can be obtained from the microarray expression data and θ_j^{PP} is the unknown parameter vector to be estimated for the *j*th pathogen protein in pathogen PPIN.

The equation (11) of the *i*th host protein can be augmented for Y_i time points as the following form,

$$\begin{bmatrix} p_j^P(t_2) \\ p_j^P(t_3) \\ \vdots \\ p_j^P(t_{Y_j}+1) \end{bmatrix} = \begin{bmatrix} \psi_j^{PP}(t_1) \\ \psi_j^{PP}(t_2) \\ \vdots \\ \psi_j^{PP}(t_{Y_j}) \end{bmatrix} \theta_j^{PP} + \begin{bmatrix} \omega_j^P(t_1) \\ \omega_j^P(t_2) \\ \vdots \\ \omega_j^P(t_{Y_j}) \end{bmatrix}, \ \text{for } j = 1, 2, \ldots, J, \tag{12}$$

which could be simply represented by,

$$P_j^P = \Phi_j^{PP}\theta_j^{PP} + \Omega_j^{PP},\ \text{for } j = 1,2,\ldots,J \tag{13}$$

where $P_j^P = \begin{bmatrix} p_j^P(t_2) \\ p_j^P(t_3) \\ \vdots \\ p_j^P(t_{Y_j}+1) \end{bmatrix}$, $\Phi_j^{PP} = \begin{bmatrix} \psi_j^{PP}(t_1) \\ \psi_j^{PP}(t_2) \\ \vdots \\ \psi_j^{PP}(t_{Y_j}) \end{bmatrix}$, $\Omega_j^{PP} = \begin{bmatrix} \omega_j^P(t_1) \\ \omega_j^P(t_2) \\ \vdots \\ \omega_j^P(t_{Y_j}) \end{bmatrix}$

Next, the parameters in the vector θ_j^{PP} can be estimated by employing the following constrained least-squares estimation problem,

$$\begin{aligned} &\min_{\theta_j^{PP}} \frac{1}{2}\left\| \Phi_j^{PP}\theta_j^{PP} - P_j^P \right\|_2^2 \\ &\text{subject to} \begin{bmatrix} 0 & \cdots & 0 & 0 & \cdots & 0 & -1 & 0 & 0 \\ 0 & \cdots & 0 & 0 & \cdots & 0 & 0 & 1 & 0 \end{bmatrix} \theta_j^{PP} \le \begin{bmatrix} 0 \\ 1 \end{bmatrix} \end{aligned} \tag{14}$$

We can acquire the interaction parameters in pathogen PPIN equation (2) by resolving the parameter estimation problem in (14) with the help of the function *lsqlin* in MATLAB optimization toolbox and simultaneously ensure the pathogen protein translation rate δ_j^P to be a non-negative value and the pathogen protein degradation rate -ε_j^P to be a non-positive value; that is to say $\delta_j^P \ge 0$ and -$\varepsilon_j^P \le 0$.

Similarly, we rewrite the host GRN dynamic regulatory equation in (3) as the linear regression form below,

$$g_k^H(\mathrm{t+1}) = [p_1^H(\mathrm{t}) \ \cdots \ p_{I_k}^H \ g_k^H(\mathrm{t})m_1^H(\mathrm{t}) \ g_k^H(\mathrm{t})m_2^H(\mathrm{t}) \cdots \ g_k^H(\mathrm{t})m_{L_k}^H(\mathrm{t}) \ l_1^H(\mathrm{t})$$

$$\cdots \ l_{M_k}^H(\mathrm{t}) \ p_1^P(\mathrm{t}) \ \cdots \ p_{J_k}^P(\mathrm{t}) \ g_k^H(\mathrm{t}) \ 1] \begin{bmatrix} e_{k1}^H \\ \vdots \\ e_{kI_k}^H \\ -f_{k1}^H \\ -f_{k2}^H \\ \vdots \\ -f_{kL_k}^H \\ h_{k1}^H \\ \vdots \\ h_{kM_k}^H \\ n_{k1}^H \\ \vdots \\ n_{kJ_k}^H \\ 1-\phi_k^{\mathrm{H}} \\ \varphi_k^H \end{bmatrix} + \varpi_k^H(\mathrm{t}) \qquad (15)$$

$$\square \ \psi_k^{HG}(\mathrm{t})\theta_k^{HG} + \varpi_k^H(\mathrm{t}), \ \text{for } k = 1,2,\ldots,K$$

where ψ_k^{HG}(t) represents the regression vector that can be obtained from the microarray expression data and θ_k^{HG} is the unknown parameter vector to be estimated for the *k*th pathogen protein in host GRN.

The equation (15) of the *k*th host gene can be augmented for Y_k time points as the following form,

$$\begin{bmatrix} g_k^H(t_2) \\ g_k^H(t_3) \\ \vdots \\ g_k^H(t_{Y_k}+1) \end{bmatrix} = \begin{bmatrix} \psi_k^{HG}(t_1) \\ \psi_k^{HG}(t_2) \\ \vdots \\ \psi_k^{HG}(t_{Y_k}) \end{bmatrix} \theta_k^{HG} + \begin{bmatrix} \varpi_k^H(t_1) \\ \varpi_k^H(t_2) \\ \vdots \\ \varpi_k^H(t_{Y_k}) \end{bmatrix}, \ \text{for } k = 1,2,\ldots,K, \qquad (16)$$

which could be simply represented by,

$$G_k^H = \Phi_k^{HG}\theta_k^{HG} + \Omega_k^{HG}, \ \text{for } k = 1,2,\ldots,K \qquad (17)$$

$$\text{where } G_k^H = \begin{bmatrix} g_k^H(t_2) \\ g_k^H(t_3) \\ \vdots \\ g_k^H(t_{Y_k}+1) \end{bmatrix},\ \Phi_k^{HG} = \begin{bmatrix} \psi_k^{HG}(t_1) \\ \psi_k^{HG}(t_2) \\ \vdots \\ \psi_k^{HG}(t_{Y_k}) \end{bmatrix},\ \Omega_k^{HG} = \begin{bmatrix} \varpi_k^H(t_1) \\ \varpi_k^H(t_2) \\ \vdots \\ \varpi_k^H(t_{Y_k}) \end{bmatrix}$$

Hence, the regulatory parameters in the vector θ_k^{HG} can be estimated by employing the following constrained least-squares estimation problem,

$$\min_{\theta_k^{HG}} \frac{1}{2}\left\| \Phi_k^{HG}\theta_k^{HG} - G_k^H \right\|_2^2$$

$$\text{subject to} \begin{bmatrix} 0 & \cdots & 0 & 1 & 0 & \cdots & 0 & 0 & \cdots & 0 & 0 & \cdots & 0 & 0 & 0 \\ \vdots & \ddots & \vdots & 0 & \ddots & \ddots & \vdots & \vdots & \ddots & \vdots & \vdots & \ddots & \vdots & \vdots & \vdots \\ \vdots & \ddots & \vdots & \vdots & \ddots & \ddots & 0 & \vdots & \ddots & \vdots & \vdots & \ddots & \vdots & \vdots & \vdots \\ 0 & \cdots & 0 & 0 & \cdots & 0 & 1 & 0 & \cdots & 0 & 0 & \cdots & 0 & 0 & 0 \\ 0 & \cdots & 0 & 0 & \cdots & \cdots & 0 & 0 & \cdots & 0 & 0 & \cdots & 0 & 1 & 0 \end{bmatrix} \theta_k^{HG} \le \begin{bmatrix} 0 \\ \vdots \\ \vdots \\ 0 \\ 1 \end{bmatrix} \quad (18)$$

We can acquire the regulatory parameters in host GRN equation (3) by resolving the parameter estimation problem in (18) with the help of the function *lsqlin* in MATLAB optimization toolbox and simultaneously ensure the host gene degradation rate -ϕ_k^H is guaranteed to be a non-positive value and the host miRNA repression rate -f_{kl}^H to be a non-positive value; that is to say -$f_{kl}^H \le 0$ for $k=1,\ldots, K_j$ and -$\phi_k^H \le 0$.

Similarly, we rewrite the host-miRNA dynamic regulatory equation as the linear regression form below,

$$m_l^H(\mathrm{t}+1) = [p_1^H(\mathrm{t}) \ \cdots \ p_{I_l}^H \ \ m_l^H(\mathrm{t})m_1^H(\mathrm{t}) \ m_l^H(\mathrm{t})m_2^H(\mathrm{t}) \cdots \ m_l^H(\mathrm{t})m_{R_l}^H(\mathrm{t}) \ \ p_1^P(\mathrm{t})$$

$$\cdots \ p_{J_l}^P(\mathrm{t}) \ \ m_l^H(\mathrm{t}) \ 1] \begin{bmatrix} o_{l1}^H \\ \vdots \\ o_{lI_l}^H \\ -q_{l1}^H \\ -q_{l2}^H \\ \vdots \\ -q_{lR_l}^H \\ r_{l1}^P \\ \vdots \\ r_{lJ_l}^P \\ 1-\gamma_l^{\mathrm{H}} \\ \kappa_l^H \end{bmatrix} + \eta_l^H(\mathrm{t}) \quad (19)$$

$$\square \ \psi_l^{HM}(\mathrm{t})\theta_l^{HM} + \eta_l^H(\mathrm{t}), \text{ for } l = 1,2,\ldots,L$$

where ψ_l^{HM}(t) represents the regression vector that can be obtained from the microarray expression data and θ_l^{HM} is the unknown parameter vector to be estimated for the *l*th host miRNA in host GRN.

The equation (19) of the *l*th host miRNA can be augmented for Y_l time points as the following form,

$$\begin{bmatrix} m_l^H(t_2) \\ m_l^H(t_3) \\ \vdots \\ m_l^H(t_{Y_l}+1) \end{bmatrix} = \begin{bmatrix} \psi_l^{HM}(t_1) \\ \psi_l^{HM}(t_2) \\ \vdots \\ \psi_l^{HM}(t_{Y_l}) \end{bmatrix} \theta_l^{HM} + \begin{bmatrix} \eta_l^H(t_1) \\ \eta_l^H(t_2) \\ \vdots \\ \eta_l^H(t_{Y_l}) \end{bmatrix}, \text{ for } l = 1,2,\ldots,L, \tag{20}$$

which could be simply represented by,

$$M_l^H = \Phi_l^{HM}\theta_l^{HM} + \Omega_l^{HM}, \text{ for } l = 1,2,\ldots,L \tag{21}$$

$$\text{where } M_l^H = \begin{bmatrix} m_l^H(t_2) \\ m_l^H(t_3) \\ \vdots \\ m_l^H(t_{Y_l}+1) \end{bmatrix}, \Phi_l^{HM} = \begin{bmatrix} \psi_l^{HM}(t_1) \\ \psi_l^{HM}(t_2) \\ \vdots \\ \psi_l^{HM}(t_{Y_l}) \end{bmatrix}, \Omega_l^{HM} = \begin{bmatrix} \eta_l^H(t_1) \\ \eta_l^H(t_2) \\ \vdots \\ \eta_l^H(t_{Y_l}) \end{bmatrix}$$

Next, the regulatory parameters in the vector θ_l^{HM} can be estimated by employing the following constrained least-squares estimation problem,

$$\min_{\theta_l^{HM}} \frac{1}{2}\left\| \Phi_l^{HM}\theta_l^{HM} - M_l^H \right\|_2^2$$
$$\text{subject to} \begin{bmatrix} 0 & \cdots & 0 & 1 & 0 & \cdots & 0 & 0 & \cdots & 0 & 0 & 0 \\ \vdots & \ddots & \vdots & 0 & \ddots & \ddots & \vdots & \vdots & \ddots & \vdots & \vdots & \vdots \\ \vdots & \ddots & \vdots & \vdots & \ddots & \ddots & 0 & \vdots & \ddots & \vdots & \vdots & \vdots \\ 0 & \cdots & 0 & 0 & \cdots & 0 & 1 & 0 & \cdots & 0 & 0 & 0 \\ 0 & \cdots & 0 & 0 & \cdots & \cdots & 0 & 0 & \cdots & 0 & 1 & 0 \end{bmatrix} \theta_l^{HM} \le \begin{bmatrix} 0 \\ \vdots \\ \vdots \\ 0 \\ 1 \end{bmatrix} \tag{22}$$

We can acquire the regulatory parameters in host GRN equation (4) by resolving the parameter estimation problem in (22) with the help of the function *lsqlin* in MATLAB optimization toolbox and simultaneously ensure the host miRNA degradation rate -γ_l^H is guaranteed to be a non-positive value and the host miRNA repression rate -q_{lr}^H to be a non-positive value; that is to say -q_{lr}^H≤0 for *r=1,…,* R_l and -γ_l^H ≤ 0.

Similarly, we rewrite the host-lncRNA dynamic regulatory equation as the linear regression form below,

$$
\begin{aligned}
l_m^H(\mathrm{t}+1) &= [p_1^H(\mathrm{t}) \cdots p_{I_m}^H(\mathrm{t}) \quad l_m^H(\mathrm{t})m_1^H(\mathrm{t}) \\
&\cdots l_m^H(\mathrm{t})m_{L_m}^H(\mathrm{t}) \; l_m^H(\mathrm{t}) \quad 1]
\begin{bmatrix} s_{m1}^H \\ \vdots \\ s_{mI_m}^H \\ -t_{m1}^H \\ \vdots \\ -t_{mL_m}^H \\ 1-\mu_m^H \\ \pi_m^H \end{bmatrix} + \vartheta_m^H(\mathrm{t}) \\
&\triangleq \psi_m^{HL}(\mathrm{t})\theta_m^{HL} + \vartheta_m^H(\mathrm{t}), \text{ for } m = 1,2,\ldots,M
\end{aligned}
\tag{23}
$$

where ψ_m^{HL}(t) represents the regression vector that can be obtained from the microarray expression data and θ_m^{HL} is the unknown parameter vector to be estimated for the *l*th host lncRNA in host GRN.

The equation (23) of the *m*th host lncRNA can be augmented for Y_m time points as the following form,

$$
\begin{bmatrix} l_m^H(t_2) \\ l_m^H(t_3) \\ \vdots \\ l_m^H(t_{Y_m}+1) \end{bmatrix} = \begin{bmatrix} \psi_m^{HL}(t_1) \\ \psi_m^{HL}(t_2) \\ \vdots \\ \psi_m^{HL}(t_{Y_m}) \end{bmatrix} \theta_m^{HL} + \begin{bmatrix} \vartheta_m^H(t_1) \\ \vartheta_m^H(t_2) \\ \vdots \\ \vartheta_m^H(t_{Y_m}) \end{bmatrix}, \text{ for } m = 1,2,\ldots,M, \tag{24}
$$

which could be simply represented by,

$$
L_m^H = \Phi_m^{HL}\theta_m^{HL} + \Omega_m^{HL}, \text{ for } m = 1,2,\ldots,M \tag{25}
$$

$$
\text{where } L_m^H = \begin{bmatrix} l_m^H(t_2) \\ l_m^H(t_3) \\ \vdots \\ l_m^H(t_{Y_m}+1) \end{bmatrix}, \Phi_m^{HL} = \begin{bmatrix} \psi_m^{HL}(t_1) \\ \psi_m^{HL}(t_2) \\ \vdots \\ \psi_m^{HL}(t_{Y_m}) \end{bmatrix}, \Omega_m^{HL} = \begin{bmatrix} \vartheta_m^H(t_1) \\ \vartheta_m^H(t_2) \\ \vdots \\ \vartheta_m^H(t_{Y_m}) \end{bmatrix}
$$

Thence, the regulatory parameters in the vector θ_m^{HL} can be estimated by employing the following constrained least-squares estimation problem,

$$
\begin{aligned}
&\min_{\theta_m^{HL}} \frac{1}{2}\left\|\Phi_m^{HL}\theta_m^{HL} - \mathrm{L}_m^H\right\|_2^2 \\
&\text{subject to} \begin{bmatrix} 0 & \cdots & 0 & 1 & 0 & \cdots & 0 & 0 \\ \vdots & \ddots & \vdots & 0 & \ddots & \ddots & \cdots & \vdots \\ 0 & \cdots & 0 & \vdots & \ddots & 1 & \ddots & \vdots \\ 0 & \cdots & 0 & 0 & \cdots & 0 & 1 & 0 \end{bmatrix} \theta_m^{HL} \le \begin{bmatrix} 0 \\ \vdots \\ 0 \\ 1 \end{bmatrix}
\end{aligned}
\tag{26}
$$

We can acquire the regulatory parameters in host GRN equation (5) by resolving the parameter estimation problem in (26) with the help of the function *lsqlin* in MATLAB optimization toolbox and simultaneously ensure the host lncRNA degradation rate $-\mu_m^H$ is guaranteed to be a non-positive value and the host miRNA repression rate $-t_{ml}^H$ to be a non-positive value; that is to say $-t_{ml}^H \leq 0$ for $l=1,\ldots,L_m$ and $-\mu_m^H \leq 0$.

Finaly, we rewrite the pathogen gene dynamic regulatory equation as the linear regression form below,

$$g_n^P(\mathrm{t}+1) = [p_1^H(\mathrm{t}) \cdots p_{I_n}^H \; g_n^P(\mathrm{t})m_1^H(\mathrm{t}) \; g_n^P(\mathrm{t})m_2^H(\mathrm{t}) \ldots \; g_n^P(\mathrm{t})m_{L_n}^H(\mathrm{t}) \; l_1^H(\mathrm{t}) \cdots l_{M_n}^H(\mathrm{t}) \; p_1^P(\mathrm{t}) \cdots p_{J_n}^P(\mathrm{t}) \; g_n^P(\mathrm{t}) \; 1] \begin{bmatrix} u_{n1}^P \\ \vdots \\ u_{nI_n}^P \\ -v_{n1}^P \\ -v_{n2}^P \\ \vdots \\ -v_{nL_n}^P \\ w_{n1}^P \\ \vdots \\ w_{nM_n}^P \\ x_{n1}^P \\ \vdots \\ x_{nJ_n}^P \\ 1-\phi_n^P \\ \varphi_n^P \end{bmatrix} + \varpi_n^P(\mathrm{t}) \quad (27)$$

$$\triangleq \psi_n^{PG}(\mathrm{t})\theta_n^{PG} + \varpi_n^P(\mathrm{t}), \text{ for } n = 1,2,\ldots,N$$

where $\psi_n^{PG}(\mathrm{t})$ represents the regression vector that can be obtained from the microarray expression data and θ_n^{PG} is the unknown regulatory parameter vector to be estimated for the *n*th pathogen gene in pathogen GRN.

The equation (27) of the *n*th pathogen gene can be augmented for Y_n time points as the following form,

$$\begin{bmatrix} g_n^P(t_2) \\ g_n^P(t_3) \\ \vdots \\ g_n^P(t_{Y_n}+1) \end{bmatrix} = \begin{bmatrix} \psi_n^{PG}(t_1) \\ \psi_n^{PG}(t_2) \\ \vdots \\ \psi_n^{PG}(t_{Y_n}) \end{bmatrix} \theta_n^{PG} + \begin{bmatrix} \varpi_n^P(t_1) \\ \varpi_n^P(t_2) \\ \vdots \\ \varpi_n^P(t_{Y_n}) \end{bmatrix}, \text{ for } n = 1,2,\ldots,N, \quad (28)$$

which could be simply represented by,

$$G_n^P = \Phi_n^{PG}\theta_n^{PG} + \Omega_n^{PG}, \text{ for } n = 1,2,\ldots,N \quad (29)$$

$$\text{where } G_n^P = \begin{bmatrix} g_n^P(t_2) \\ g_n^P(t_3) \\ \vdots \\ g_n^P(t_{Y_n}+1) \end{bmatrix}, \Phi_n^{PG} = \begin{bmatrix} \psi_n^{PG}(t_1) \\ \psi_n^{PG}(t_2) \\ \vdots \\ \psi_n^{PG}(t_{Y_n}) \end{bmatrix}, \Omega_n^{PG} = \begin{bmatrix} \varpi_n^P(t_1) \\ \varpi_n^P(t_2) \\ \vdots \\ \varpi_n^P(t_{Y_n}) \end{bmatrix}$$

Hence, the parameters in the vector θ_n^{HG} can be estimated by employing the following constrained least-squares estimation problem,

$$\min_{\theta_n^{PG}} \frac{1}{2}\left\| \Phi_n^{PG}\theta_n^{PG} - G_n^P \right\|_2^2$$

$$\text{subject to} \begin{bmatrix} 0 & \cdots & 0 & 1 & 0 & \cdots & 0 & 0 & \cdots & 0 & 0 & \cdots & 0 & 0 & 0 \\ \vdots & \ddots & \vdots & 0 & \ddots & \ddots & \vdots & \vdots & \ddots & \vdots & \vdots & \ddots & \vdots & \vdots & \vdots \\ \vdots & \ddots & \vdots & \vdots & \ddots & \ddots & 0 & \vdots & \ddots & \vdots & \vdots & \ddots & \vdots & \vdots & \vdots \\ 0 & \cdots & 0 & 0 & \cdots & 0 & 1 & 0 & \cdots & 0 & 0 & \cdots & 0 & 0 & 0 \\ 0 & \cdots & 0 & 0 & \cdots & \cdots & 0 & 0 & \cdots & 0 & 0 & \cdots & 0 & 1 & 0 \end{bmatrix} \theta_n^{PG} \leq \begin{bmatrix} 0 \\ \vdots \\ \vdots \\ 0 \\ 1 \end{bmatrix} \quad (30)$$

We can acquire the regulatory parameters in pathogen GRN equation (6) by resolving the parameter estimation problem in (30) with the help of the function *lsqlin* in MATLAB optimization toolbox and simultaneously ensure the pathogen gene degradation rate $-\phi_n^P$ is guaranteed to be a non-positive value and the host miRNA repression rate $-v_{nl}^P$ to be a non-positive value; that is to say $-v_{nl}^P \leq 0$ for $l=1,\ldots, L_n$ and $-\phi_n^P \leq 0$.

As the mentioned parameter estimation problem of dynamic models above, to prevent overfitting problem in the parameter identification and obtain the accurate results of the system identification method, we apply the cubic spline to interpolate some extra numbers of data points (5 times number of the parameters in the corresponding parameter vector, i.e., θ_i^{HP} in human PPIN, θ_j^{PP} in pathogen PPIN, θ_k^{HG} in human-gene GRN, θ_l^{HM} in human miRNA GRN, θ_m^{HL} in human-lncRNA GRN, θ_n^{PG} in pathogen-gene GRN to be estimated). Then, with the microarray expression data, the solutions of the constrained least-square parameter estimation problems in (10), (14), (18), (22), (26) and (30) could be obtained for accurate parameter identification in GEINs gene by gene (or protein by protein) by using the function *lsqlin* in MATLAB optimization toolbox for the optimal estimations of parameters in these estimation problems. Moreover, since the measurement technology of genome-wide protein expression of OKF6/TERT-2 cells and *C. albicans* has not been implemented yet, and about 73% variance of protein abundance can be explained by the corresponding mRNA abundance [4], that is to say, the microarray data of gene expressions can substitute protein expressions and contribute to sufficient information for resolving the above constrained least-squares parameter estimation problems in (10), (14), (18), (22), (26) and (30).

1.4. Trimming False-positives in Candidate GEINs by System Order Detection Scheme

Because candidate GEINs contain many false-positive interactions and regulations acquired from computational, experimental and homology-dependent predictions in the database mining process, we must employ system order detection scheme for host PPI model in (1), pathogen PPI model in (2), host-gene GRN model in (3), host-miRNA GRN model in (4), host -lncRNA model in (5) and pathogen-gene GRN model in (6) to prune these false-positives in the candidate GEINs. Hence, we apply Akaike information criterion (AIC) to deleting the insignificant parameters out of the system order of the candidate GEINs by the real microarray data of OKF6/TERT-2 cells during *C. albicans* SC5314 and *C. albicans* WO-1 infection, respectively.

In host PPI model in (9), AIC of the host PPIs of the *i*th host protein can be defined as the function of system interaction order as follows [1–3],

$$AIC_i^{HP}\left(N_i,J_i\right)=\log\left(\frac{1}{T_i}\left(P_i^H-\Phi_i^{HP}\hat{\theta}_i^{HP}\right)^T\left(P_i^H-\Phi_i^{HP}\hat{\theta}_i^{HP}\right)\right)+\frac{2\left(N_i+J_i\right)}{T_i} \tag{31}$$

where $\hat{\theta}_i^{HP}$ represents the estimated interactive parameters of human-protein i from the solutions of the parameter estimation problem in (10), and the covariance of estimated residual error is $(\sigma_i^{HP})^2=\frac{1}{T_i}\left(P_i^H-\Phi_i^{HP}\hat{\theta}_i^{HP}\right)^T\left(P_i^H-\Phi_i^{HP}\hat{\theta}_i^{HP}\right)$. According to system identification theory [3], the real system order $N_i^*+J_i^*$ of the real PPIs of protein i in the host PPI could minimize $AIC_i^{HP}(N_i, J_i)$. By this system order detection method, host proteins with insignificant interaction abilities out of N_i^* and pathogen proteins with insignificant interaction abilities are out of J_i^* should be considered as false positives and trimmed from the candidate PPIs of ith protein. By a similar procedure could obtain the real host PPIs one protein by one protein in GEINs.

Similarly, in pathogen PPIN model (13), AIC of pathogen-protein j could be defined as follows [3],

$$AIC_j^{PP}\left(O_j,I_j\right)=\log\left(\frac{1}{T_j}\left(P_j^P-\Phi_j^{PP}\hat{\theta}_j^{PP}\right)^T\left(P_j^P-\Phi_j^{PP}\hat{\theta}_j^{PP}\right)\right)+\frac{2\left(O_j+I_j\right)}{T_j} \tag{32}$$

where $\hat{\theta}_j^{PP}$ denotes the estimated interactive parameters of pathogen-protein j obtained from the solutions of the parameter estimation problem in (14), and the covariance of estimated residual error is $(\sigma_j^{PP})^2=\frac{1}{T_j}\left(P_j^P-\Phi_j^{PP}\hat{\theta}_j^{PP}\right)^T\left(P_j^P-\Phi_j^{PP}\hat{\theta}_j^{PP}\right)$. We could minimize AIC_j^{PP} in (32) to achieve at the real interaction numbers O_j^* and I_j^* of the real PPIs of protein j in the pathogen PPIN. Therefore, based on the real system order O_j^* and I_j^* can be used to prune the false positive interactions of candidate PPIN one protein by one protein for the real pathogen PPIN of GEINs.

By the similar procedure, in host-gene regulation model (17), AIC of host-gene k could be defined as follows [3],

$$AIC_k^{HG}\left(I_k,L_k,M_k,J_k\right)=\log\left(\frac{1}{T_k}\left(G_k^H-\Phi_k^{HG}\hat{\theta}_k^{HG}\right)^T\left(G_k^H-\Phi_k^{HG}\hat{\theta}_k^{HG}\right)\right)+\frac{2\left(I_k+L_k+M_k+J_k\right)}{T_k} \tag{33}$$

where $\hat{\theta}_k^{HG}$ denotes the estimated regulatory parameters of host-gene k obtained from the solutions of the parameter estimation problem in (18), and the covariance of estimated residual error is

$(\sigma_k^{HG})^2 = \frac{1}{T_k}\left(G_k^H - \Phi_k^{HG}\hat{\theta}_k^{HG}\right)^T \left(G_k^H - \Phi_k^{HG}\hat{\theta}_k^{HG}\right)$. We could minimize AIC_k^{HG} in (33) to achieve the real regulation numbers I_k^*, L_k^*, M_k^* and J_k^* of the real regulations of host-gene k in the host-gene GRN. Therefore, the corresponding real system order I_k^*, L_k^*, M_k^* and J_k^* can be used to prune the false positive regulations of candidate GRN one gene by one gene for the real host-gene GRN of GEINs.

By the similar procedure, in host-miRNA regulation model (21), AIC of host-miRNA l could be defined as follows [3],

$$AIC_l^{HM}\left(I_l, R_l, J_l\right) = \log\left(\frac{1}{T_l}\left(M_l^H - \Phi_l^{HM}\hat{\theta}_l^{HM}\right)^T \left(M_l^H - \Phi_l^{HM}\hat{\theta}_l^{HM}\right)\right) + \frac{2\left(I_l + R_l + J_l\right)}{T_l} \quad (34)$$

where $\hat{\theta}_l^{HM}$ denotes the estimated regulatory parameters of host-miRNA l obtained from the solutions of the parameter estimation problem in (22), and the covariance of estimated residual error is $(\sigma_l^{HM})^2 = \frac{1}{T_l}\left(M_l^H - \Phi_l^{HM}\hat{\theta}_l^{HM}\right)^T \left(M_l^H - \Phi_l^{HM}\hat{\theta}_l^{HM}\right)$. We could minimize AIC_l^{HM} in (34) to achieve the real regulation numbers I_l^*, R_l^* and J_l^* of the real regulations of host-miRNA l in the host-miRNA GRN. Therefore, the corresponding real system order I_l^*, R_l^* and J_l^* can be used to prune the false positive regulations one host-miRNA by one host-miRNA for the real host-miRNA GRN of GEINs.

By the similar procedure, in host-lncRNA regulation model (25), AIC of host-lncRNA m could be defined as follows [3],

$$AIC_m^{HL}\left(I_m, L_m\right) = \log\left(\frac{1}{T_m}\left(L_m^H - \Phi_m^{HL}\hat{\theta}_m^{HL}\right)^T \left(L_m^H - \Phi_m^{HL}\hat{\theta}_m^{HL}\right)\right) + \frac{2\left(I_m + L_m\right)}{T_m} \quad (35)$$

where $\hat{\theta}_m^{HL}$ stands for the estimated regulatory parameters of host-lncRNA m obtained from the solutions of parameter estimation problem in (26), and the covariance of estimated residual error is $(\sigma_m^{HL})^2 = \frac{1}{T_m}\left(L_m^H - \Phi_m^{HL}\hat{\theta}_m^{HL}\right)^T \left(L_m^H - \Phi_m^{HL}\hat{\theta}_m^{HL}\right)$. We could minimize AIC_m^{HL} in (35) to achieve the real regulation number I_m^* and L_m^* of the real regulations of host-lncRNA m in the host-lncRNA GRN. Therefore, the corresponding real system order I_m^* and L_m^* can be used to prune the false positive regulations one lncRNA by one lncRNA for the real host-lncRNA GRN of GEINs.

Finally, in pathogen-gene regulation model (29), AIC of pathogen-gene n could be defined as follows [3],

$$AIC_n^{PG}\left(I_n, L_n, M_n, J_n\right) = \log\left(\frac{1}{T_n}\left(G_n^P - \Phi_n^{PG}\hat{\theta}_n^{PG}\right)^T \left(G_n^P - \Phi_n^{PG}\hat{\theta}_n^{PG}\right)\right) + \frac{2\left(I_n + L_n + M_n + J_n\right)}{T_n} \quad (36)$$

where $\hat{\theta}_n^{PG}$ stands for the estimated regulatory parameters of pathogen-gene n obtained from the solutions of the parameter estimation problem in (30), and the covariance of estimated residual error is $(\sigma_n^{PG})^2 = \frac{1}{T_n}\left(G_n^P - \Phi_n^{PG}\hat{\theta}_n^{PG}\right)^T\left(G_n^P - \Phi_n^{PG}\hat{\theta}_n^{PG}\right)$. We could minimize AIC_n^{PG} in (36) to achieve the real regulation numbers I_n^*, L_n^*, M_n^* and J_n^* of the real regulations of pathogen-gene n in the pathogen-gene GRN. Therefore, the corresponding real system order I_n^*, L_n^*, M_n^* and J_n^* can be used to prune the false positive regulations one pathogen gene by pathogen gene for the real pathogen-gene GRN of GEINs.

After applying this AIC approach to identify the system order and prune the false-positives of candidate GEINs, we eventually get the real GEINs of the OKF6/TERT-2 cells under the infection of *C. albicans* SC5314 and *C. albicans* WO-1 for each replicate, respectively (Figure S3). Since the complexity of real GEINs, it is very difficult to investigate the accurate host-pathogen cross-talk mechanisms from them. We thereby introduce PNP approach to extract the core host-pathogen cross-talk networks (HPCNs) structures from real GEINs which could help us to investigate the cross-talk mechanisms and get more insights into the pathogenesis of infection by different strains of *C. albicans*. Moreover, the core host-pathogen cross-talk networks (HPCNs) structures of *C. albicans* SC5314 and *C. albicans* WO-1 are shown in Figure S4 and Figure S5 respectively.

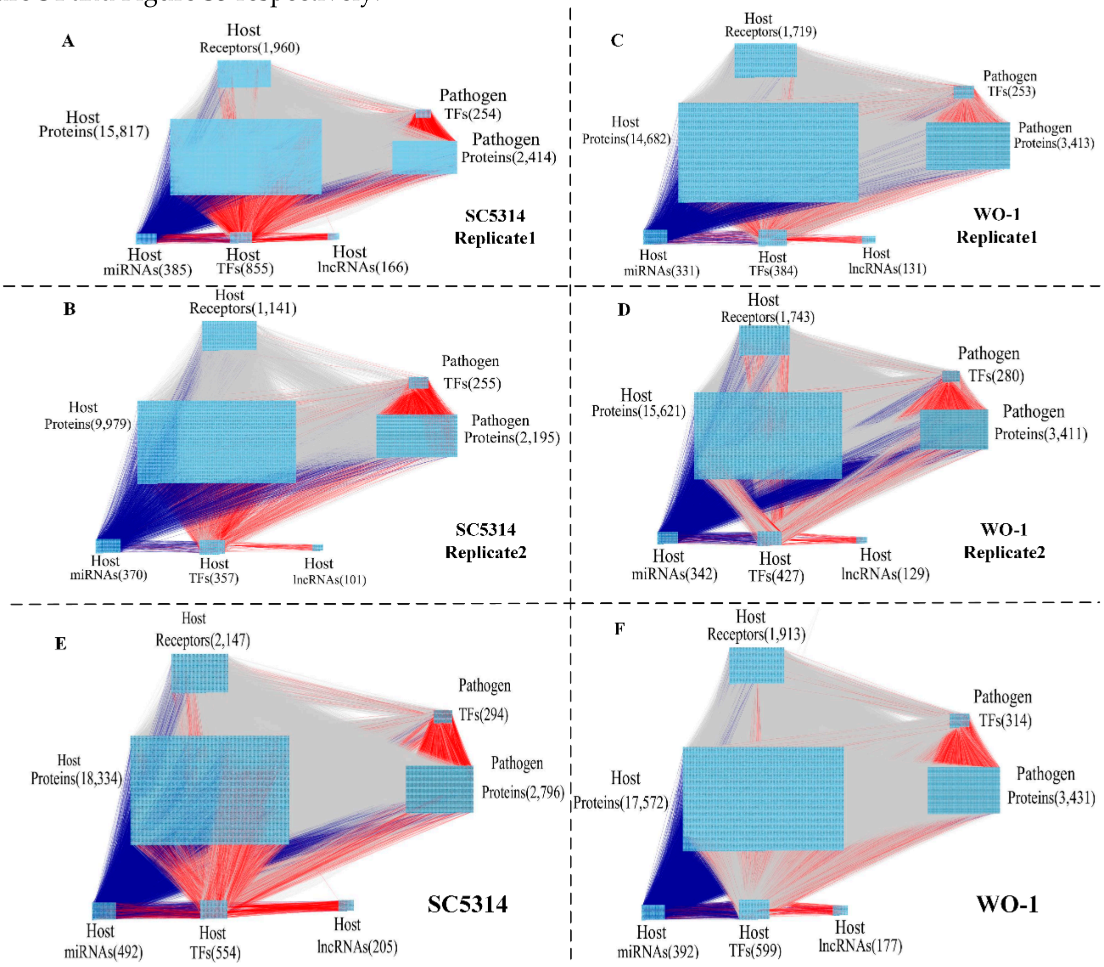

Figure 3. The real GEINs of two replicates during the infection of *C. albicans* SC5314 and *C. albicans* WO-1 with OKF6/TERT-2 cells, respectively. Figures S3(A) and S3(B) reveal the recognized real genome-wide GEINs of each replicate during *C. albicans* SC5314 infection. Figures S3(C) and S3(D) reveal the recognized real genome-wide GEINs of each replicate during *C. albicans* WO-1 infection. In Figures S3(A) and S3(B), the

real GEINs of all two replicates during the *C. albicans* SC5314 infection are integrated in Figure S3(E). In Figures S3(C) and S3(D), the real GEINs of two replicates during the *C. albicans* WO-1 infection are integrated in Figure S3(F). The grey lines indicate protein-protein interaction; the red lines represent the transcriptional regulation, and the blue lines denote miRNA repression.

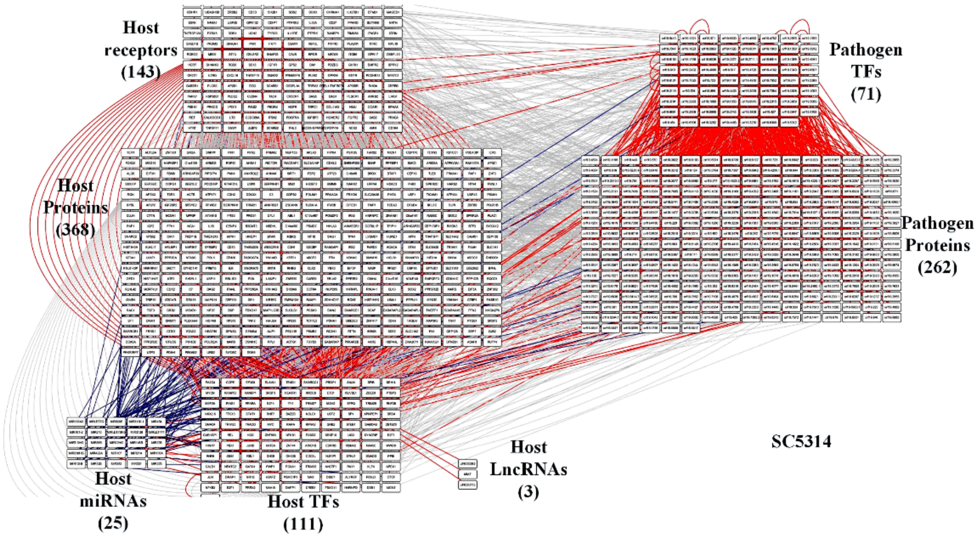

Figure 4. Core HPCN of OKF6/TERT-2 cells during the infection of C. albicans SC5314. This core HPCN was extracted from the real GEIN in Figure S3(E) via PNP method. The grey lines denote protein-protein interaction; the red lines represent the transcriptional regulation, and the blue lines indicate miRNA repression.

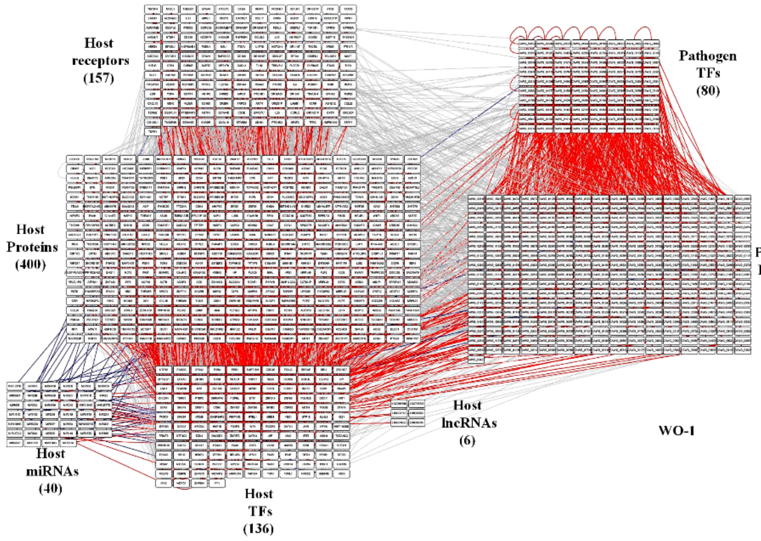

Figure 5. Core HPCN of OKF6/TERT-2 cells during the infection of *C. albicans* WO-1. This core HPCN was extracted from the real GEIN in Figure S3(F) via PNP method. The grey lines denote protein-protein interaction; the red lines represent the transcriptional regulation, and the blue lines indicate miRNA repression.

1.5. Extracting Core Host-pathogen Cross-talk Networks (HPCNs) Structures from Real GEINs by using PNP Approach

Before applying the PNP approach to extract the core host-pathogen cross-talk networks (HPCNs) from the real GEINs, it is essential to construct a combined network matrix *H* of a real GEIN. Furthermore,

the combined network matrix H includes all estimated interaction and regulation parameters in the real GEIN as follows,

$$H=\begin{bmatrix} H_{hp,hp} & H_{hp,pp} & 0 & 0 \\ H_{pp,hp} & H_{pp,pp} & 0 & 0 \\ H_{hg,hp} & H_{hg,pp} & H_{hg,hm} & H_{hg,hl} \\ H_{hm,hp} & H_{hm,pp} & H_{hm,hm} & 0 \\ H_{hl,hp} & 0 & H_{hl,hm} & 0 \\ H_{pg,hp} & H_{pg,pp} & H_{pg,hm} & H_{pg,hl} \end{bmatrix} \in \mathbb{R}^{(2I+2J+L+M)\times(I+J+L+M)}$$

where $H_{hp,hp}=\begin{bmatrix} \hat{a}_{11}^{H} & \cdots & \hat{a}_{1I}^{H} \\ \vdots & \hat{a}_{in}^{H} & \vdots \\ \hat{a}_{I1}^{H} & \cdots & \hat{a}_{II}^{H} \end{bmatrix}, H_{hp,pp}=\begin{bmatrix} \hat{b}_{11}^{H} & \cdots & \hat{b}_{1J}^{H} \\ \vdots & \hat{b}_{ij}^{H} & \vdots \\ \hat{b}_{I1}^{H} & \cdots & \hat{b}_{IJ}^{H} \end{bmatrix}, H_{pp,hp}=\begin{bmatrix} \hat{d}_{11}^{P} & \cdots & \hat{d}_{1I}^{P} \\ \vdots & \hat{d}_{ji}^{P} & \vdots \\ \hat{d}_{J1}^{P} & \cdots & \hat{d}_{JI}^{P} \end{bmatrix},$

$$H_{pp,pp}=\begin{bmatrix} \hat{c}_{11}^{P} & \cdots & \hat{c}_{1J}^{P} \\ \vdots & \hat{c}_{jo}^{P} & \vdots \\ \hat{c}_{J1}^{P} & \cdots & \hat{c}_{JJ}^{P} \end{bmatrix}, H_{hg,hp}=\begin{bmatrix} \hat{e}_{11}^{H} & \cdots & \hat{e}_{1I}^{H} \\ \vdots & \hat{e}_{ki}^{H} & \vdots \\ \hat{e}_{I1}^{H} & \cdots & \hat{e}_{II}^{H} \end{bmatrix}, H_{hg,pp}=\begin{bmatrix} \hat{n}_{11}^{H} & \cdots & \hat{n}_{1J}^{H} \\ \vdots & \hat{n}_{kj}^{H} & \vdots \\ \hat{n}_{I1}^{H} & \cdots & \hat{n}_{IJ}^{H} \end{bmatrix},$$

$$H_{hg,hm}=\begin{bmatrix} -\hat{f}_{11}^{H} & \cdots & -\hat{f}_{1L}^{H} \\ \vdots & -\hat{f}_{kl}^{H} & \vdots \\ -\hat{f}_{I1}^{H} & \cdots & -\hat{f}_{IL}^{H} \end{bmatrix}, H_{hg,hl}=\begin{bmatrix} \hat{h}_{11}^{H} & \cdots & \hat{h}_{1M}^{H} \\ \vdots & \hat{h}_{km}^{H} & \vdots \\ \hat{h}_{I1}^{H} & \cdots & \hat{h}_{IM}^{H} \end{bmatrix}, H_{hm,hp}=\begin{bmatrix} \hat{o}_{11}^{H} & \cdots & \hat{o}_{1I}^{H} \\ \vdots & \hat{o}_{li}^{H} & \vdots \\ \hat{o}_{L1}^{H} & \cdots & \hat{o}_{LI}^{H} \end{bmatrix},$$

$$H_{hm,pp}=\begin{bmatrix} \hat{r}_{11}^{P} & \cdots & \hat{r}_{1J}^{P} \\ \vdots & \hat{r}_{lj}^{P} & \vdots \\ \hat{r}_{L1}^{P} & \cdots & \hat{r}_{LJ}^{P} \end{bmatrix}, H_{hm,hm}=\begin{bmatrix} -\hat{q}_{11}^{H} & \cdots & -\hat{q}_{1L}^{H} \\ \vdots & -\hat{q}_{lr}^{H} & \vdots \\ -\hat{q}_{L1}^{H} & \cdots & -\hat{q}_{LL}^{H} \end{bmatrix}, H_{hl,hp}=\begin{bmatrix} \hat{s}_{11}^{H} & \cdots & \hat{s}_{1I}^{H} \\ \vdots & \hat{s}_{mi}^{H} & \vdots \\ \hat{s}_{M1}^{H} & \cdots & \hat{s}_{MI}^{H} \end{bmatrix},$$

$$H_{hl,hm}=\begin{bmatrix} -\hat{t}_{11}^{H} & \cdots & -\hat{t}_{1L}^{H} \\ \vdots & -\hat{t}_{ml}^{H} & \vdots \\ -\hat{t}_{M1}^{H} & \cdots & -\hat{t}_{ML}^{H} \end{bmatrix}, H_{pg,hp}=\begin{bmatrix} \hat{u}_{11}^{P} & \cdots & \hat{u}_{1I}^{P} \\ \vdots & \hat{u}_{ni}^{P} & \vdots \\ \hat{u}_{J1}^{P} & \cdots & \hat{u}_{JI}^{P} \end{bmatrix}, H_{pg,pp}=\begin{bmatrix} \hat{x}_{11}^{P} & \cdots & \hat{x}_{1J}^{P} \\ \vdots & \hat{x}_{nj}^{P} & \vdots \\ \hat{x}_{J1}^{P} & \cdots & \hat{x}_{JJ}^{P} \end{bmatrix},$$

$$H_{pg,hm}=\begin{bmatrix} -\hat{v}_{11}^{p} & \cdots & -\hat{v}_{1L}^{p} \\ \vdots & -\hat{v}_{nl}^{p} & \vdots \\ -\hat{v}_{J1}^{p} & \cdots & -\hat{v}_{JL}^{p} \end{bmatrix}, H_{pg,hl}=\begin{bmatrix} \hat{w}_{11}^{P} & \cdots & \hat{w}_{1M}^{P} \\ \vdots & \hat{w}_{nm}^{P} & \vdots \\ \hat{w}_{J1}^{P} & \cdots & \hat{w}_{JM}^{P} \end{bmatrix}$$

where $\hat{a}_{in}^{H}$ and $\hat{b}_{ij}^{H}$ could be acquired in $\hat{\theta}_{i}^{HP}$ by resolving the parameter estimation problem in (10) and pruning false positives by AIC method in (31); $\hat{d}_{ji}^{P}$, and $\hat{c}_{jo}^{H}$ could be acquired in $\hat{\theta}_{j}^{PP}$ by resolving the parameter estimation problem in (14) and pruning false positives by AIC method in (32); $\hat{e}_{ki}^{H}$, $\hat{n}_{kj}^{H}$, $-\hat{f}_{kl}^{H}$ and $\hat{h}_{km}^{H}$ could be acquired in $\hat{\theta}_{k}^{HG}$ by resolving the parameter estimation problem in (18) and trimming

false positives by AIC method in (33); $\hat{o}_{li}^{H}$, $\hat{r}_{lj}^{P}$ and $-\hat{q}_{lr}^{H}$ could be acquired in $\hat{\theta}_{l}^{HM}$ by resolving the parameter estimation problem in (22) and pruning false positives by AIC method in (34); $\hat{s}_{mi}^{H}$ and $-\hat{t}_{ml}^{H}$ could be acquired in $\hat{\theta}_{m}^{HL}$ by resolving the parameter estimation problem in (26) and pruning false positives by AIC method in (35); and $\hat{u}_{ni}^{P}$, $\hat{x}_{nj}^{P}$, $-\hat{v}_{nl}^{P}$ and $\hat{w}_{nm}^{P}$ could be acquired in $\hat{\theta}_{n}^{PG}$ by resolving the parameter estimation problem in (30) and pruning false positives by AIC method in (36). $\hat{a}_{in}^{H}$ and $\hat{c}_{jo}^{P}$ denote the interactive abilities of intra-species in host and pathogen PPINs during the pathogen infection, respectively; $\hat{b}_{ij}^{H}$ and $\hat{d}_{ji}^{P}$ represent the interactive abilities between host protein i and pathogen protein j in the inter-species PPIN; $\hat{e}_{ki}^{H}$, $\hat{o}_{li}^{H}$, $\hat{s}_{mi}^{H}$ and $\hat{u}_{ni}^{P}$ denote the regulatory abilities of human TF i to regulate human-gene k, human-miRNA l, human-lncRNA m, and pathogen-gene n, respectively, in human-gene GRN, human-miRNA GRN, human-lncRNA GRN and pathogen-gene GRN. $\hat{n}_{kj}^{H}$, $\hat{r}_{lj}^{P}$ and $\hat{x}_{nj}^{P}$ signify the regulatory abilities of pathogen TF j to regulate human-gene k, human-miRNA l and pathogen-gene n, respectively, in human-gene GRN, human-miRNA GRN and pathogen-gene GRN during the pathogen infection, respectively. $-\hat{f}_{kl}^{H}$, $-\hat{q}_{lr}^{H}$, $-\hat{t}_{ml}^{H}$ and $-\hat{v}_{nl}^{p}$ correspond to the repression abilities of human miRNA l to inhibit human-gene k, human-miRNA r, human-lncRNA m, and pathogen-gene n, respectively, in human-gene GRN, human-miRNA GRN, human-lncRNA GRN and pathogen-gene GRN. $\hat{h}_{km}^{H}$ and $\hat{w}_{nm}^{P}$ indicate the regulatory abilities of human lncRNA m to regulate human-gene k and pathogen-gene n, respectively, in human-gene GRN and pathogen-gene GRN. All of these estimated interactions and regulations compose of the combined network matrix H. Note that if connections or regulations have been removed via AIC or not been built in candidate GEIN via big data mining, the corresponding parameters in matrix H are padded with zero.

We thereby extract the core components in the real GEIN by PNP approach, which is a significant network structure projection approach on the basis of the principal singular values to reduce network dimension via deleting insignificant structures. Accordingly, the combined network matrix H can be denoted by singular value decomposition form below,

$$H = U \times D \times V^{T} \tag{37}$$

where $U \in \mathbb{R}^{(2I+2J+L+M)\times(I+J+L+M)}$; $V \in \mathbb{R}^{(I+J+L+M)\times(I+J+L+M)}$ and $D = \mathbf{diag}(d_1, \ldots, d_s, \ldots d_{I+J+L+M})$ is the diagonal matrix of $d_1, d_2, \ldots, d_s, \ldots, d_{I+J+L+M}$ which includes the $I+J+L+M$ singular values of the combined network matrix H in descending order, i.e., $d_1 \geq \ldots \geq d_s \geq \ldots \geq d_{I+J+L+M}$. Note that **diag** ($d_1$, d_2) signifies the diagonal matrix of d_1 and d_2. However, we can define the eigen expression fraction (E_s) as the normalization of singular values,

$$E_s = \frac{d_s^2}{\sum_{s=1}^{I+J+L+M} d_s^2}, \text{s} = 1, 2, \ldots, I + J + L + M \tag{38}$$

From the viewpoint of energy, we need to keep the main system energy of the whole network structure in the PNP projection. Therefore, we choose the minimum Z such that $\sum_{s=1}^{Z} E_s \geq 0.85$, that is, the top Z singular vectors of network matrix H containing 85% network structure of GEIN which is composed of these top Z principal components from the viewpoint of energy. Next, we define the projection of H to the top Z singular vectors of U and V, respectively, as follows,

$$V\left(w_R, s\right) = h_{w_R,:} \times v_{:,s}^T$$
$$w_R = 1, \ldots, 2I + 2J + L + M \text{ and } s = 1, \ldots, Z \quad (39)$$

where $h_{w_R,:}$ and $v_{:,s}^T$ denote the the w_Rth row of H and the sth row of V, respectively. Eventually, we define and apply the 2-norm projection value of each node, including gene, miRNA, lncRNA and protein in the real GEIN to the top Z right-singular vectors and left-singular vectors in the following,

$$D\ (w_R) = \left[\sum_{s=1}^{Z}\left[V_R\left(w_R, s\right)\right]^2\right]^{1/2},$$
$$w_R = 1, \ldots, 2I + 2J + L + M \quad (40)$$

The meaning of (40) is that the closer projection value D (w_R) approaches zero, the much more unimportant to corresponding w_R node within the core network composed of the top Z singular vectors. In other words, the larger the projection value is, the more contribution made by the node to the core network. Consequently, we can extract core HPCNs from the real GEIN of the *C. albicans* SC5314 and *C. albicans* WO-1 infection, respectively, by evaluating the projection value of each node in (40). Since the aim of this study is considered to identify the common and specific pathogenic mechanisms of infection progression of *C. albicans* SC5314 and WO-1, we target the core host/pathogen proteins with the highest projection value, and their connecting TF/miRNA/lncRNA to form the core cross-talk pathways in respect of KEGG pathways so that we can systematically investigate pathogenic mechanisms for drug design.

References

1. Chen, B.S.; Wu.C.C. *Systems Biology*; Nova Science: New York, NY, USA, 2014.
2. Chen, B.S.; li.C.W. *Big mechanisms in systems biology*. Academic Press: New York, NY, USA, 2017.
3. Johansson, R. *System modeling and identification*. prentice hall: englewood cliffs, NJ, USA, 1993.
4. Lu, P.; Vogel C Fau - Wang, R.; Wang R Fau - Yao, X.; Yao X Fau - Marcotte, E.M.; Marcotte, E.M. Absolute protein expression profiling estimates the relative contributions of transcriptional and translational regulation. *Nat Biotechnol.* **2007**, *25*, 117–124.

© 2019 by the authors. Licensee MDPI, Basel, Switzerland. This article is an open access article distributed under the terms and conditions of the Creative Commons Attribution (CC BY) license (http://creativecommons.org/licenses/by/4.0/).
